# Discovering in vivo cytokine-eQTL interactions from a lupus clinical trial

**DOI:** 10.1186/s13059-018-1560-8

**Published:** 2018-10-19

**Authors:** Emma E. Davenport, Tiffany Amariuta, Maria Gutierrez-Arcelus, Kamil Slowikowski, Harm-Jan Westra, Yang Luo, Ciyue Shen, Deepak A. Rao, Ying Zhang, Stephen Pearson, David von Schack, Jean S. Beebe, Nan Bing, Sally John, Michael S. Vincent, Baohong Zhang, Soumya Raychaudhuri

**Affiliations:** 1 Center for Data Sciences, Brigham and Women’s Hospital, Boston, MA 02115 USA; 2 Divisions of Genetics and Rheumatology, Department of Medicine, Brigham and Women’s Hospital, Harvard Medical School, Boston, MA 02115 USA; 3 Partners Center for Personalized Genetic Medicine, Boston, MA 02115 USA; 4 Program in Medical and Population Genetics, Broad Institute of MIT and Harvard, Cambridge, MA 02142 USA; 5 Department of Biomedical Informatics, Harvard Medical School, Boston, MA 02115 USA; 6 Department of Cell Biology, Harvard Medical School, Boston, MA 02115 USA; 7 Division of Rheumatology, Allergy, Immunology, Brigham and Women’s Hospital, Harvard Medical School, Boston, MA 02115 USA; 8 Pfizer Inc., Cambridge, MA 02139 USA; 9 Pfizer New Haven Clinical Research Unit, New Haven, CT 06511 USA; 10 Biogen, Cambridge, MA 02142 USA; 11 Faculty of Medical and Human Sciences, University of Manchester, M13 9PL, Manchester, UK; 12 Harvard New Research Building, 77 Avenue Louis Pasteur, Suite 250D, Boston, MA 02446 USA

**Keywords:** eQTL, Interactions, Clinical trials, Cytokines

## Abstract

**Background:**

Cytokines are critical to human disease and are attractive therapeutic targets given their widespread influence on gene regulation and transcription. Defining the downstream regulatory mechanisms influenced by cytokines is central to defining drug and disease mechanisms. One promising strategy is to use interactions between expression quantitative trait loci (eQTLs) and cytokine levels to define target genes and mechanisms.

**Results:**

In a clinical trial for anti-IL-6 in patients with systemic lupus erythematosus, we measure interferon (IFN) status, anti-IL-6 drug exposure, and whole blood genome-wide gene expression at three time points. We show that repeat transcriptomic measurements increases the number of *cis* eQTLs identified compared to using a single time point. We observe a statistically significant enrichment of in vivo eQTL interactions with IFN status and anti-IL-6 drug exposure and find many novel interactions that have not been previously described. Finally, we find transcription factor binding motifs interrupted by eQTL interaction SNPs, which point to key regulatory mediators of these environmental stimuli and therefore potential therapeutic targets for autoimmune diseases. In particular, genes with IFN interactions are enriched for ISRE binding site motifs, while those with anti-IL-6 interactions are enriched for IRF4 motifs.

**Conclusions:**

This study highlights the potential to exploit clinical trial data to discover in vivo eQTL interactions with therapeutically relevant environmental variables.

**Electronic supplementary material:**

The online version of this article (10.1186/s13059-018-1560-8) contains supplementary material, which is available to authorized users.

## Background

Cytokines are critical signals used by the immune system to coordinate inflammatory responses. These factors bind to specific receptors to induce widespread transcriptional effects. Cytokines and their receptors are not only genetically associated with susceptibility to a range of human diseases; they have also emerged as effective therapeutic targets [1]. Blockade of tumor necrosis factor (TNF) was the first cytokine-directed therapy to achieve widespread use and is now used broadly to treat multiple inflammatory diseases including rheumatoid arthritis (RA), psoriasis, and inflammatory bowel disease [2]. More recently, IL-6 has emerged as a compelling therapeutic target. IL-6 levels are elevated in autoimmune diseases such as systemic lupus erythematosus (SLE) and RA. The IL-6 receptor has been successfully targeted with tocilizumab in RA [3] and giant cell arteritis [4], while IL-6 has been targeted directly with siltuximab for successful treatment of Castleman’s disease [5]. In SLE, IL-6 is thought to play a role in the observed B cell hyperactivity and autoantibody production [6]. Targeting IL-6-R in SLE has shown promise in phase I trials [7], and this has led to the development of other biologics targeting IL-6 such as PF-04236921 [8]. Interferon (IFN)-α, produced primarily by plasmacytoid dendritic cells, has pleiotropic effects on the immune system. It has been implicated as a key mechanism in SLE development and pathogenesis and is being investigated as a therapeutic target [9]. Agents targeting other inflammatory cytokines, including interleukin-1 (IL-1), IL-12, IL-17A, and IL-23 are also in clinical use to treat autoimmune conditions. Interestingly, IL-1 blockade with canakinumab has also been recently reported to reduce risk of heart attacks, stroke, and cardiovascular disease [10]. Therefore, defining the regulatory consequences of physiologic perturbations of cytokine levels will inform our understanding of both disease and drug mechanisms.

A *cis* expression quantitative trait locus (eQTL) contains a genetic variant that alters expression of a nearby gene. *Cis* eQTLs are ubiquitous across the genome [11], and while most are stable across tissues and conditions, environmental variables can alter the effects of some of them [12–18]. If an environmental change leads to disruption of regulators upstream of a gene, then it could magnify or dampen an eQTL effect, resulting in a genotype-by-environment interaction (Additional file 1: Figure S1). Therefore, observing a set of eQTL interactions due to a perturbagen, such as a cytokine, can identify shared upstream regulatory mechanisms, such as transcription factors and key pathways. Alternatively, a set of shared eQTL interactions may be the consequence of a cellular subpopulation whose frequency is being altered by the perturbagen. Even a single eQTL interaction where we can define mechanism can lead to insights about the action of the perturbagen.

However, *cis* eQTL interactions with physiologic environmental factors in humans have been challenging to discover in vivo [19–23] even with large cohorts [11, 17]. Success at finding *cis* eQTL interactions has largely been found in studies using model organisms [24, 25] or treating cells in vitro with non-physiologic conditions [26]. Thus far, these studies might be limited in power since they often map eQTLs separately across conditions and fail to exploit the power of repeat measurements [27]. In other instances, they test for genetic variants associated with differential expression and miss information about the magnitude of the eQTL effect in a specific condition [28].

We predicted that if the transcriptome is assayed at multiple time points under different exposure states, then the repeat measurements could lead to an increase in power to detect eQTLs and their interactions with environmental perturbations. If the same individual is assessed at multiple times, then the noise in transcriptomic measurements is reduced. Furthermore, repeat measurements from the same individuals when they are both unexposed and exposed to an environmental perturbagen allow for more accurate modeling of the effect of the perturbagen within those subjects.

Clinical trials, with their structured study design, may be the ideal setting to detect eQTL interactions with therapeutically important variables. In clinical trials, it is becoming increasingly common to collect transcriptional and genetic data alongside clinical and physiological data [29]. This extensive phenotyping of therapeutically important variables and biomarkers within the same individual at multiple time points provides a unique opportunity to identify in vivo eQTL interactions.

Here, we examined the modulation of eQTL effects by environmental factors that alter cytokine levels using data from a phase II clinical trial to evaluate the safety and efficacy of a neutralizing IL-6 monoclonal antibody (PF-04236921) in 157 SLE patients [8] (“Study design”). Many patients with SLE exhibit high levels of genes induced by type I IFN; these genes, known as the IFN signature, are a marker of disease severity [30, 31] and a pathogenic feature of SLE. This feature of the disease, together with exposure to anti-IL-6 leads to cytokine fluctuations in this cohort yielding opportunities to assess the impact of cytokine levels on eQTL effects. While this drug was not significantly different from placebo for the primary efficacy endpoint (proportion of patients achieving the SLE Responder Index (SRI-4) at week 24), biologically it effectively reduced free IL-6 protein levels (Additional file 1: Figure S2). Given the key role of IL-6 and IFN in a range of diseases, the downstream regulatory effects of these cytokines are of great interest to study.

In this study, we leverage the power of repeat transcriptional and environmental measurements from a lupus clinical trial to identify in vivo eQTL interactions with IFN status and anti-IL-6 exposure. In the process, we define novel eQTL interactions for both IFN and IL-6.

## Results

We conducted whole blood high-depth RNA-seq profiling at 0, 12, and 24 weeks in anti-IL-6 exposed and unexposed individuals with the Illumina TruSeq protocol. We quantified 20,253 gene features and examined 1,595,793 genotyped and imputed common variants genome-wide (“RNA sequencing”, “Genotyping”, “Imputation”). Along with each RNA-seq assay, we documented anti-IL-6 exposure and quantified IFN signature status with real-time PCR.

### Mapping eQTL in SLE patients

We first mapped *cis* eQTLs and then tested them for interactions with IFN status and anti-IL-6 exposure. eQTL interactions can be explored using our interactive visualization tool (http://baohongz.github.io/Lupus_eQTL [32], Additional file 1**:** Figure S3).

**Fig. 1:**
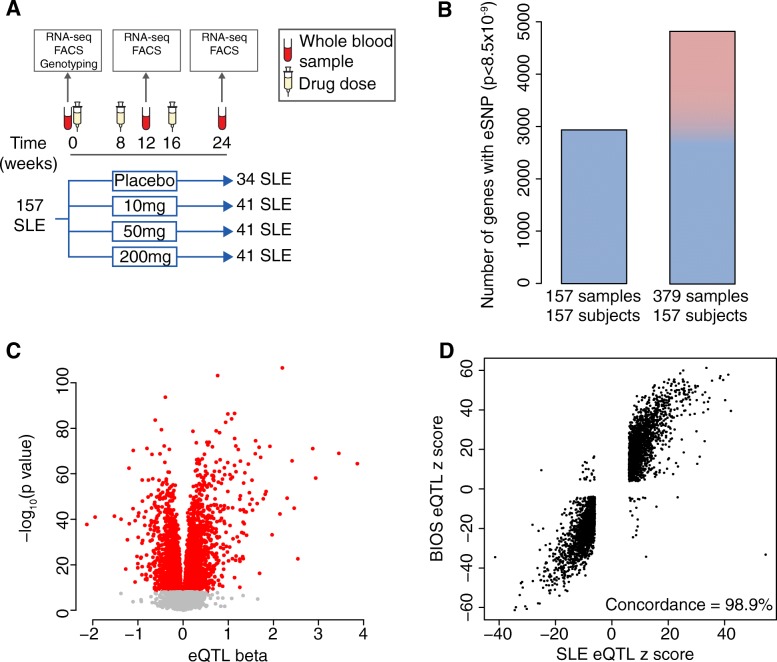
Identifying eQTLs in SLE patients. **a** Clinical trial structure and sampling strategy for the individuals used for eQTL analysis. The samples available are summarized in Table 1. **b** Number of eQTL genes identified using a linear model (left) and a linear mixed model (right). For the linear model, we used the first available time point for each individual (week 0 sample for *n* = 152, week 12 sample for *n* = 5). **c** Volcano plot of eQTL effects for the most significantly associated SNP for each gene (red color indicates *p* < 8.5 × 10^− 9^). **d** Concordance of SLE eQTL effects (*p* < 8.5 × 10^− 9^) with eQTLs observed in the BIOS cohort [11] of healthy individuals (FDR < 0.05). Each point represents the most significant SNP-gene pair for the SLE eQTL

To identify *cis* eQTLs, we examined the association between gene expression and SNPs within 250 kb upstream of the transcription start site and 250 kb downstream of the transcription end site. In order to account for repeat measurements, with up to three RNA-seq assays per patient (Fig. 1a, 379 samples from 157 patients, “eQTL and interaction analysis”), we used a linear mixed model. We included 25 gene expression principal components to maximize the number of eQTL detected and 5 genotyping principal components to account for the heterogeneity in ethnicity in our cohort (“eQTL and interaction analysis”). We observed that the multi-ethnic nature of our study did not confound our results, consistent with Stranger et al. [33] (Additional file 1**:** Figure S4).

To ensure we only tested for interactions in a set of highly confident eQTLs, we applied a stringent correction for the total number of hypotheses tested. We recognized that this approach might arguably be overly stringent for eQTL discovery, but we wanted to be certain that we were only testing eQTLs for interactions that had a convincing main effect. Since we tested a total of 5,872,001 SNP-gene pairs genomewide, we set a significance threshold of *p*_eqtl_ < 8.5 × 10^− 9^ (0.05/5,872,001 tests). We identified 4818 *cis* eQTL genes (Fig. 1b, c, Additional file 2: Table S1). The summary statistics for all the gene SNP pairs tested are available through figshare [34].

To confirm the validity of our eQTLs, we compared them to a larger dataset. In the BIOS cohort, consisting of 2166 healthy individuals [11], we observed that 85.4% of our SLE eQTL SNP-gene pairs are reported as eQTLs (FDR < 0.05). Of these, 98.9% showed consistent direction of effect (*p* < 5 × 10^− 16^, binomial test, Fig. 1d), suggesting that our results were highly concordant with those in this substantially larger study.

### Repeat measurements increase power to detect eQTL

Under reasonable assumptions, we would expect repeat samples to increase our power. Supporting that expectation, we detected 64% more *cis* eQTLs compared to the 2934 genes from using a single sample (first available time point) per individual (Fig. 1b). An alternative might have been to identify eQTLs separately from each of the three time points; however, this approach identified only a total of 3050 eQTL genes (Additional file 1: Figure S5). Modeling all three time points together results in 58% more *cis* eQTLs than modeling each time point separately.

We speculated that while repeat measures did increase power over single measures, that given a fixed number of samples, independent samples would lead to more power. To this end, we conducted an analysis fixing the number of samples at 157 and using 53 individuals with repeat measures (with two missing samples). Unsurprisingly, we found fewer eQTLs (2215 genes) with the repeat measures alone compared to an analysis with the same number of independent samples (2934 genes).

### IFN status eQTL interactions

**Fig. 2:**
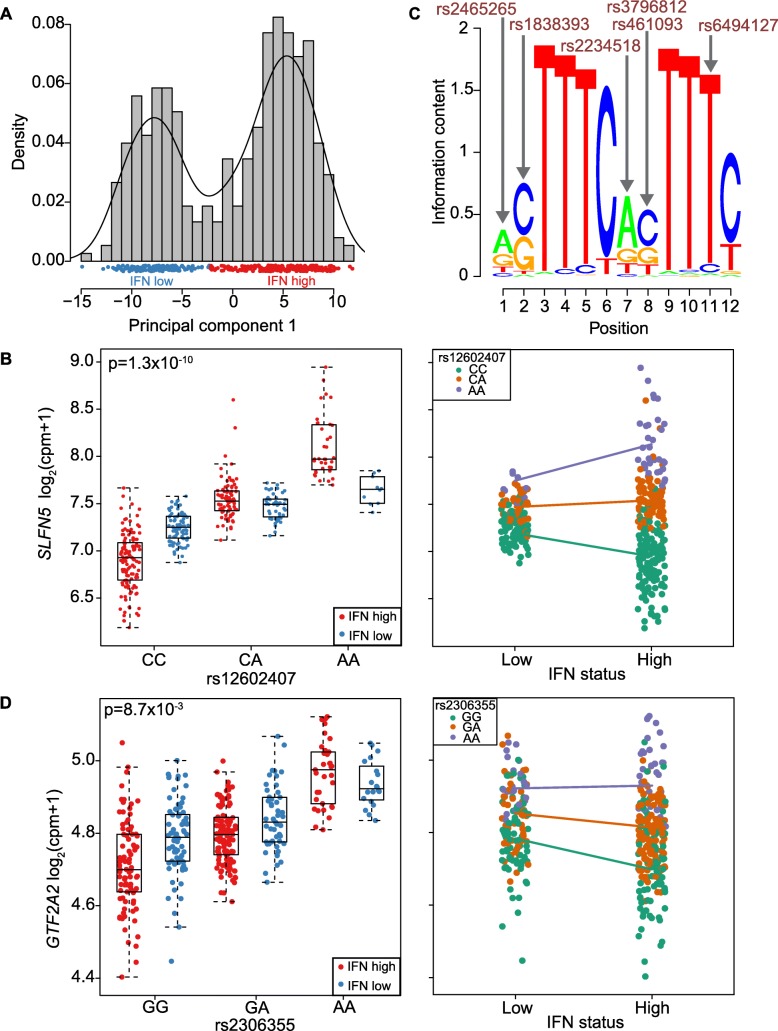
eQTL interactions with IFN status. **a** Designation of IFN status for each sample from the real-time PCR expression of 11 genes (first principal component). **b** IFN status interaction with the *SLFN5* eQTL plotted with respect to rs12602407 genotype (left) and IFN status of the sample (right). **c** The ISRE motif enriched among eQTLs magnified in IFN high samples. Arrows indicate positions of the motif interrupted by interaction SNPs (or SNPs in strong LD). Red indicates these SNPs correspond to magnified eQTLs. **d** IFN status interaction with the *GTF2A2* eQTL plotted with respect to rs2306355 genotype (left) and IFN status of the sample (right)

For each of the 4818 *cis* eQTL genes, we tested the most significantly associated SNP for environmental interactions with our linear mixed model framework. We first explored the influence of type I IFN on gene regulation after determining the IFN status of every patient at each time point. We classified each sample as either IFN high or IFN low using real-time PCR of 11 IFN-inducible genes [35] (“Interferon status”, Fig. 2a).

We first wanted to assess whether our results were indeed enriched for interactions. To do this, we identified those eQTLs with nominally significant interaction effects at *p*_interact_ < 0.01. We would expect ~ 48 out of 4818 from chance alone. Surprisingly, we observed 182 IFN-eQTL interactions (Additional file 2: Table S1) that were nominally significant at *p*_interact_ < 0.01 suggesting that there was evidence of enrichment for eQTL interactions. We conducted permutations to ensure that these results were not the consequence of potentially inflated statistics, which might be the result for example of low-frequency alleles, genes violating normality assumptions, or other technical artifacts. In each of 1000 stringent permutations, we simply reassigned IFN status across samples and retested for eQTL interactions. This permutation preserves the main eQTL effect, since it maintains genotypes of the individuals with the associated expression data, but disrupts any real interactions that might be present in the data. In 0 out of 1000 instance did we observe 182 or more interactions at *p*_interact_ < 0.01 suggesting that the number of observed interactions is enriched and highly unlikely to have happened by chance (Additional file 1: Figure S6, *p*_permute_ ~ 0/1000 = < 0.001).

We then went on to identify those specific IFN-eQTL interactions of greatest interest by calculating a false discovery rate or *q* value for each interaction using the *q* value package [36] (“eQTL and interaction analysis”). We observed a total of 210 interactions with an FDR < 0.2 threshold (111 with FDR < 0.1 and 67 with FDR < 0.05, Additional file 2**:** Table S1). We note that 11 of these genes have already been described as having an interaction with a proxy gene for type I IFN signaling in the much larger BIOS study [11]. For example, *SLFN5* expression is influenced by the rs12602407 SNP (*p*_interact_ = 1.3 × 10^− 10^, FDR < 9.9 × 10^− 8^, Fig 2b), and this effect is magnified in IFN high samples. Of these 210 IFN-eQTL interactions, 99 were not reported in the BIOS study [11]. Indeed, applying a more stringent cut off of FDR < 0.01, 27/34 of our interactions are not previously reported and therefore are almost certainly novel IFN-eQTL interactions with high confidence (Additional file 1: Figure S7).

We speculated that groups of eQTL interactions might be driven by the same common regulatory factor. We divided interactions into magnifiers, where the environmental exposure increases the size of the eQTL effect, and dampeners where the environmental exposure decreases the eQTL effect (Additional file 1: Figure S8). We hypothesized that the transcription factors driving the response to type I IFN may be different for the eQTL interactions defined as magnifiers (*n* = 127, FDR < 0.2) and dampeners (*n* = 83, FDR < 0.2).

We applied HOMER [37] to assess overlap between transcription factor binding motifs and the eQTL interaction SNPs (and SNPs in high linkage disequilibrium (LD, *r*^2^ > 0.8) in the *cis* window, “eQTL and interaction analysis”). To determine enrichment, we compared the transcription factor motifs found in a set of sequences (containing the interaction SNPs) from one category of interactions relative to the other. We conducted two separate analyses: the proportion of magnifying eQTL interaction sequences with a motif compared to the proportion of dampening interaction sequences with a motif and vice versa. We found enrichment of motifs for key transcription factors involved in IFN signaling including a statistically significant enrichment for the ISRE motif (HOMER *p* = 1 × 10^− 4^, Additional file 2: Table S2). The ISRE motif disruption occurred for 11 genes with an eQTL magnified in IFN high samples but for only one gene with an eQTL dampened (permutation *p* < 0.019, “Magnifiers and dampeners”, Fig. 2c). An example is the *GTF2A2* rs2306355 eQTL (*p*_interact_ = 8.7 × 10^− 3^, FDR < 0.15, Fig. 2d); rs2306355 is in tight LD (*r*^2^ = 0.83 in Europeans) with rs6494127, which interrupts the TTCNNTTT core of the ISRE motif (Fig. 2c). This SNP likely disrupts IRF9 and STAT2 binding in the ISGF3 complex [38], which binds to the ISRE motif. We observe greater expression of *GTF2A2* in individuals with the rs2306355 A allele compared to G; this difference is magnified in IFN high individuals (Fig. 2d).

We included principal components as covariates in our model to account for confounding sources of gene expression variation that are not limited to those that have been measured in the study. Additional file 2: Table S3 summarizes the correlation between the principal components and potential known confounders such as age, sex, and site of recruitment. As no single principal component strongly correlates with these known confounders, we re-ran the interaction analysis including age and sex as fixed effects and site as a random effect. The interaction betas are very highly correlated (*r*_s_ = 0.99) with the original effects suggesting the principal components are capturing these known confounders (Additional file 1**:** Figure S9).

We considered that the principal components included as covariates in our model might be mitigating power. For example, the 4th principal component of gene expression is correlated with the IFN signature status of the sample (*r*_s_ = − 0.7, Additional file 2: Table S3), so we repeated the IFN interaction analysis without correcting for principal component 4. For all the eQTLs tested for an IFN interaction, we observed very similar results with highly correlated z-scores (*r*_s_ = 0.94, Additional file 1: Figure S10). To further explore this, we also repeated the IFN interaction analysis without correcting for any expression principal components. While we find the betas for the interaction term are highly correlated (*r*_s_ = 0.88, Additional file 1: Figure S11a), only 23/210 of our IFN eQTL interactions remain significant with an FDR < 0.2 without correcting for any expression principal components. This reduction in significant interactions is likely due to the larger standard errors of the interaction estimate that are observed when principal components are not corrected for (Additional file 1: Figure S11b). Furthermore, 107/210 of these interactions no longer have a main eQTL effect (passing our Bonferroni corrected *p* value threshold) without principal component correction, further reducing our power to detect significant interactions.

### Discovery of eQTL interactions with anti-IL-6 drug exposure

We then examined whether IL-6 blockade alters the relationship between genomic variation and gene expression and induces drug-eQTL interactions. We wanted to first test if there was evidence of such interactions in our data set. Again, using a threshold of *p*_interact_ < 0.01 for nominal significance for interactions, we observed 121 drug-eQTL interactions with anti-IL-6 out of 4818 eQTLs tested (Additional file 2: Table S1); similar to IFN interactions, this is far in excess of the ~ 48 we would expect by chance. As above, to ensure that these results were not the consequence of statistical artifact, we applied the same stringent permutation strategy, reassigning which samples were exposed or not to anti-IL-6. After 1000 permutations, we never observed as many as 121 drug-eQTL interactions with *p*_interact_ < 0.01 (Additional file 1: Figure S12), suggesting that our eQTLs were indeed highly enriched for those interacting with anti-IL-6 (*p*_permute_ ~ 0/1000 < 0.001).

We analyzed drug and IFN-eQTL interactions independently because anti-IL-6 exposure and IFN status are not associated (Fisher’s exact test *p* = 0.6). However, to further ensure that these variables are independent, we repeated the interaction analysis with a full model including the drug, drug interaction, IFN, and IFN interaction terms. We find that the interaction betas are highly concordant (*r*_s_ = 0.99) with the original analysis (Additional file 1**:** Figure S13) for both IFN and drug-eQTL interactions providing further evidence that IFN status and drug exposure are independent.

**Fig. 3:**
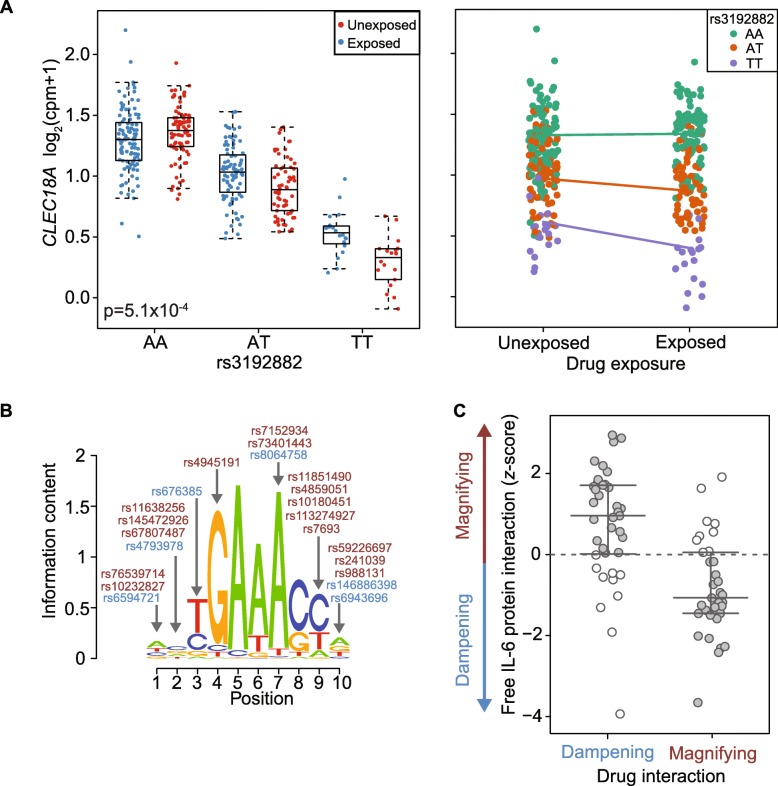
eQTL interactions with drug exposure. **a** Drug exposure interaction with the *CLEC18A* eQTL plotted with respect to rs3192882 genotype (left) and drug exposure (right). **b** The IRF4 motif enriched among eQTLs magnified following drug treatment. Arrows indicate positions of the motif interrupted by interaction SNPs (or SNPs in strong LD). Red and blue indicate SNPs corresponding to magnified and dampened eQTLs respectively. **c** Concordance of free IL-6 protein interaction effects with drug exposure interaction effects (gray indicates consistent direction)

To identify specific eQTL events that interact with anti-IL-6, we again calculated a false discovery rate. We observed that 72 of these interactions have an FDR < 0.2 (7 with FDR < 0.1 and 1 with FDR < 0.05, Additional file 2: Table S1). Only eight of these drug-eQTL interactions overlap with the interactions observed for IFN status (Additional file 2: Table S1). We note biologically relevant drug-eQTL interactions for *IL10* (*p*_interact_ = 2.6 × 10^− 3^, FDR < 0.19, Additional file 1: Figure S14), an anti-inflammatory cytokine, *CLEC4C* (*p*_interact_ = 2.9 × 10^− 3^, FDR < 0.19) which has previously been associated in *trans* with an SLE risk allele [39] and *CLEC18A* (*p*_interact_ = 5.1 × 10^− 4^, FDR < 0.14, Fig. 3a) another member of the C-type lectin domain family.

Similar to the IFN-eQTL interactions, we divided the drug-eQTL interactions into magnifiers (*n* = 33, FDR < 0.2) and dampeners (*n* = 39, FDR < 0.2) (Additional file 1: Figure S15) and used the approach as described above to define transcription factors potentially driving the response to IL-6 blockade (Additional file 2**:** Table S4). One of the motifs enriched for eQTLs magnified after drug treatment (compared to dampeners) was IRF4 (HOMER *p* = 1 × 10^− 3^). The IRF4 motif disruption occurred for nine genes, including *CLEC18A*, with an eQTL magnified after drug treatment compared to four genes with an eQTL dampened (Fig. 3b, “Magnifiers and dampeners”). We permuted the magnifying and dampening genes and found this ratio for enrichment is interesting at the gene level but not significant (*p* = 0.058) and therefore additional eQTL interactions will be necessary to confirm.

### Comparing differential expression to eQTL interactions

A more common strategy to determine the effect of an environmental variable is to use differential gene expression. For IFN status, we identified 1850 differentially expressed genes (FDR < 0.05 and fold change > 1.2, Additional file 1: Figure S16, Additional file 2: Table S5). Only 42/210 IFN-eQTL interaction genes also show evidence of differential expression. For differential expression following anti-IL-6 treatment, we identified 394 genes (FDR < 0.05 and fold change > 1.2, Additional file 1: Figure S16, Additional file 2: Table S6). Only 2/72 drug-eQTL interaction genes show evidence of differential gene expression. This suggests that eQTL interactions offer independent information from differential expression, which might contribute to defining mechanisms.

### Concordance of drug-eQTL interactions with protein level interactions

We hypothesized that interactions due to drug exposure are likely driven by free IL-6 cytokine levels (our key clinical biomarker of interest). If this is the case, for eQTLs dampened by drug exposure, an increase in free IL-6 should elicit an opposite interaction effect and result in eQTL magnification. We assessed whether eQTL interactions with free IL-6 protein levels measured in the patient serum samples were consistent with those following IL-6 blockade. We observed enrichment in the overlap between cytokine interactions and drug interactions (53/72 interactions in expected opposite direction, Fig. 3c, *p* = 3.8 × 10^− 5^, binomial test).

### Contribution of cell proportions to eQTL interactions

Given that we have conducted our study on whole blood, the observed eQTL interactions could be the consequence of a cellular subpopulation whose frequency is being altered by the environmental perturbagen or variability in cell type proportions between individuals. To explore this, we first determined B and T cell abundance from FACS data (“Cell counts”). We find that IFN and exposure to anti-IL-6 are not correlated with either B or T cell abundance (Additional file 1: Figure S17) suggesting that the proportions of these particular cell types are not being altered by the shifts in cytokine levels.

We then went on to explore the effect of correcting for cell proportions on our eQTL interaction effects. As our FACS data do not cover all relevant cell types, we also inferred the relative proportions of nine hematopoietic populations from the RNA-seq data using CIBERSORT [40]. For IFN, the interaction betas remain highly correlated (*r*_s_ = 0.99, *r*_s_ = 0.998, Additional file 1: Figure S18) after correcting for either B and T cell proportions from the FACS data or the nine hematopoietic proportions from CIBERSORT. Furthermore, the majority of the interactions (162/210 and 189/210) remain significant (FDR < 0.2) after these respective corrections. We observe a similar pattern for the drug-eQTL interactions (Additional file 1: Figure S19) where the interaction betas are again highly correlated (*r*_s_ = 0.98 and *r*_s_ = 0.993 for FACS and CIBERSORT proportion correction respectively). However only 15/72 and 54/72 drug-eQTL interactions remain significant after these corrections. This reduction in significant interactions suggests that some of these interactions may be related to changes in cell proportions. However, given that the interaction betas are so highly correlated and the relatively small effect size of the drug interactions, this could also be the result of reduced power to detect interactions following the inclusion of additional covariates in the model.

## Discussion

In this study we mapped eQTLs in a clinical trial of SLE patients and discovered interactions with IFN and IL-6, two clinically important cytokines. Our study had dramatic variation in IL-6 that was therapeutically induced, and variation in IFN due to the disease status of the SLE patients. This, together with the structured study design with repeat measurements of gene expression across different conditions in the same individual, allowed us to identify in vivo eQTL interactions.

eQTL interactions with drug interventions or other therapeutically relevant physiologic variables are important to identify as they can point to regulatory mechanisms, such as transcription factors or subclasses of enhancers, acting downstream of the environmental condition of interest and driving groups of eQTL interactions. The IFN status eQTL interactions we identified provide support for this approach. By making use of the direction of effect for the eQTL interaction, we were able to identify an enrichment of magnifying eQTL interaction SNPs interrupting the binding sites of transcription factors known to be important in the response to IFN, such as ISGF3 (the STAT1, STAT2, and IRF9 complex), which binds ISRE. Once we are able to recognize the downstream drivers of therapeutically relevant clinical variables, then it may become possible to define more mechanisms of action for drugs and more precise drug targets.

As a powerful example, we note enrichment of magnifying anti-IL-6-eQTL interaction SNPs interrupting the binding site of IRF4. It has been suggested that IRF4 works downstream of IL-6 by binding BATF and coordinately regulating the production of IL10 and other genes [41]. Consistent with this, we observed that the *IL10* eQTL does indeed interact with presence of anti-IL-6 (Additional file 1: Figure S14). Previous studies have highlighted a role for IRF4 in the pathogenesis of autoimmune diseases in mouse and humans. For example in a murine model of SLE, *IRF4* knockout mice did not develop lupus nephritis [42]. In humans, IRF4 is associated with RA [43], a disease in which anti-IL-6 treatment has been successful [3]. Our findings provide further support that IRF4 could be a potential therapeutic target for autoimmune diseases such as RA where anti-IL-6 is effective [44].

The ability to focus on interactions with specific patient phenotypes might point to key targets for disease intervention. For example, IFN is a key immunophenotype in SLE patients, and elevated in SLE compared to healthy controls [30, 31]. The IFN status immunophenotype is already itself driving interest in therapeutic targets. A recent phase II clinical trial has shown that an antagonist to the type I IFN receptor, acting upstream of ISRE, reduced severity of symptoms in SLE. Interestingly, the antagonist was more effective in the patients with a high baseline IFN status [45]. This example provides a compelling case study for how understanding master regulators of key disease phenotypes might lead to promising new therapeutic strategies. We speculate that this provides a mechanism for stratified medicine for future studies, which may be applicable to other diseases.

We recognized that computing eQTL interactions requires a robust statistical model that accounts for genotype, environmental factor, RNA expression levels, repeat measurements, and technical covariates. We were sensitive to the possibility that pre-processing and normalization of these factors could potentially have an impact on our results. For this reason, we used stringent filtering and examined only variants that were common and where the minor allele was present for each of the exposure groups. Next, to confirm enrichment of eQTL interactions, we used a stringent permutation-based strategy that preserved the distribution of genotypes and corresponding expression values. Finally, we also utilized a standard normal transformation [46] (“eQTL and interaction analysis”) and observed that this had little effect on the primary eQTL analysis (*r*_s_ = 0.99 for *z* scores, Additional file 1: Figure S20) and interaction analyses (IFN *r*_s_ = 0.84, drug *r*_s_ = 0.76 for z scores, Additional file 1: Figure S21), or the observed enrichment over the null in our stringent permutation analysis (Additional file 1: Figure S22).

We acknowledge that our approach for eQTL discovery using a stringent Bonferroni corrected *p* value threshold is conservative and could reduce our ability to detect eQTLs with a modest effect in one group and therefore reduce the number of interactions we observe. However, given the challenge of identifying interactions, we wanted to ensure that we were confident in the eQTL effect before testing that effect for an interaction. Furthermore, as demonstrated by the *SLFN5* IFN-eQTL interaction, we still observe interaction examples where an eQTL effect is very modest in one group, in this case, samples designated as IFN low (Fig. 2b).

While we find that the majority of the eQTL interactions that we identify are independent of differentially expressed genes, we have used the common strategy for identifying differential expression, which does not take into account the genotype of the individuals. The differential expression presented here therefore represents the average change in expression across all genotypes, regardless of any eQTL or interaction effect. Furthermore, like most differential expression approaches, we have employed a fold change cut-off. Using statistical evidence alone, 121/210 IFN-eQTL interaction genes show evidence of differential expression (FDR< 0.05) and 45/72 drug-eQTL interaction genes. This approach highlights that many interactions are being driven by changes in variance of gene expression across the environmental variables rather than necessarily changes in mean expression and therefore eQTL interactions can offer additional information to what is identified through traditional differential expression analysis.

We note that as we have conducted our study on whole blood, some of our observed interactions could be driven by variability in cell type proportions between individuals or as a consequence of cellular subpopulation frequencies being altered by the environmental perturbagen. A limitation of this study is that we lack the complete blood counts to explore this thoroughly. However, we determined B and T cell abundance from FACS data and used CIBERSORT [40] to deconvolute the relative proportions of nine hematopoietic populations from the RNA-seq data to explore this (“Cell counts”). While the number of significant eQTL interactions is reduced after correcting for cell populations, particularly for drug-eQTL interactions after correcting for the FACS proportions, the interaction effects remain very highly correlated suggesting that the majority of these effects are not being altered by these cell compositions. However, further studies will be required to determine if cytokine shifts are altering cellular populations that were not detected by these actual or inferred cell counts, or the principal components that we included in our analyses. For future studies, it will be informative to quantify a broader range of relative cell types and data from single-cell technologies may be particularly powerful for determining cell type-specific eQTLs [47] and their interactions.

We speculate that drug-eQTL interactions might offer an alternative pharmacogenetic strategy to assess drug response. For many biologic medications, predictive pharmacogenetics through typical association studies has been challenging; for example, studies trying to define genetic or transcriptomic biomarkers of anti-TNF response have not been successful [48, 49]. An eQTL interaction approach can be used to define a genotype-aware score reflecting the biological activity that a medication is having upon an individual, given their allelic combination of multiple genetic markers. For example, we can define a simple anti-IL-6 exposure score based on 7 anti-IL-6 eQTL interactions with a more stringent FDR (FDR < 0.1). The rationale for this drug exposure score is that the expression of a drug-eQTL interaction gene will reflect the effectiveness of the drug in the individual but will be dependent on the genotype of the eQTL interaction SNP. The score is therefore based on assessing whether the expression of the eQTL target gene was more consistent with the drug exposed or the unexposed state for the corresponding interaction SNP genotype. Unsurprisingly, we found a difference in drug exposure score between the unexposed and exposed samples (Additional file 1**:** Figure S23) (*r*_s_ = 0.40, *p* = 2.1 × 10^− 16^); these differences reflect the fact that the eQTLs were themselves identified by examining samples with and without drug exposure. However, while we did not utilize the administered drug dose to identify drug-eQTL interactions, we observed a significant correlation between drug dose (10, 50, or 200 mg) and drug exposure score (*r*_s_ = 0.16, *p* = 0.02) in the drug-exposed samples (Additional file 1: Figure S24). A simple eQTL interaction score may therefore have the potential to stratify individuals when assessing response to a medication, for example, those with a higher drug exposure score may have a better response to treatment. Similarly, this score could be correlated with adverse effects to capture informative gene expression signatures.

We do not find an association between anti-IL-6 exposure and IFN status and only eight of the cytokine eQTL interactions overlap. Arguably an anti-cytokine therapeutic that is truly effective in SLE might be expected to reduce IFN levels, given how central IFN is to SLE pathogenesis [50]. However, we note a limitation of this study is that the drug itself did not achieve its primary efficacy endpoint of improving SLE outcomes. Hence, while the drug exposure score for this study tracked with the biological effect of the drug (reducing free IL-6 protein levels), it might not be useful for SLE specifically. However, such a scoring system could be implemented easily in most phase III trials for a broad range of therapeutics, where the numbers of samples are far in excess of this phase II trial, ensuring better powered and more accurate eQTL-interaction mapping.

## Conclusions

We devised a framework for identifying in vivo eQTL interactions with therapeutically relevant variables, exploiting repeat measurements from a clinical trial. We have applied this approach to demonstrate how downstream regulatory effects of cytokine biology can be elucidated. This same approach can be applied to a wide range of other clinically important cytokines, their antagonists, or indeed other targeted biologic therapies. We speculate that this approach might even be applied to the presence or absence of disease, or disease activity. However, given the multifaceted nature of disease effects, interpreting an eQTL interaction in that context might be more challenging. Modern clinical cohorts and clinical trial data sets with RNA-seq data that has been collected will make this approach easily applicable on a wide scale.

## Methods

### Study design

The objectives of this study were to map eQTLs in a cohort of lupus patients and identify eQTL interactions with environmental perturbations such as drug treatment to shed light on drug and disease mechanisms. SLE patients were recruited to a phase II clinical trial to test the efficacy and safety of an IL-6 monoclonal antibody (PF-04236921). The patient population recruited to this trial have been detailed extensively by Wallace et al. [8]. One hundred eighty-three patients (forming a multi-ethnic cohort) were randomized to receive three doses of drug (10, 50, or 200 mg) or placebo at three time points during the trial (weeks 0, 8, and 16). Table 1 summarizes the number of patients and samples available.

**Table 1:** Summary of patients and samples available for each data type. Where relevant, the number of patients/samples remaining after quality control (QC) is displayed in brackets

Data	Patients (post-QC)	Samples (post-QC)
Study design	183	549
RNA sequencing	180 (180)	468 (464)
Genotyping	160 (159)	
eQTL analysis	157	379
IFN status	157	376
Free IL-6 protein levels	145	311
T and B cell counts	152	320

### RNA sequencing

We collected peripheral venous blood samples in PAXgene Blood RNA tubes (PreAnalytiX GmbH, BD Biosciences) for high-depth RNA-seq profiling at 0, 12, and 24 weeks. We extracted total RNA from blood samples using the PAXgene Blood RNA kit (Qiagen) at a contract lab using a customized automation method. We assessed the yield and quality of the isolated RNA using Quant-iT™ RiboGreen® RNA Assay Kit (Thermo Fisher Scientific) and Agilent 2100 Bioanalyzer (Agilent Technologies), respectively. Following quality assessment, we processed an aliquot of 500–1000 ng of each RNA with a GlobinClear-Human kit (Thermo Fisher Scientific) to remove globin mRNA. We then converted RNA samples to cDNA libraries using TruSeq RNA Sample Prep Kit v2 (Illumina) and sequenced using Illumina HiSeq 2000 sequencers. We generated an average of 40 M 100 bp pair-end reads per sample for downstream analysis.

We successfully obtained 468 RNA-seq profiles from 180 patients. We aligned reads to the reference genome (GENCODE [51] release 19) and quantified gene expression using Subread [52] and featureCounts [53] respectively. We included genes with at least 10 reads (CPM > 0.38) in at least 32 samples (minimum number of patients with both unexposed and exposed RNA-seq assays in a drug group) prior to normalization. Following QC, we removed four samples as outliers. We then normalized 20,253 transcripts using the trimmed mean of *M*-values method and the edgeR R package [54]. Expression levels are presented as log_2_(cpm + 1) and available through figshare [34].

### Genotyping

We genotyped 160 individuals across 964,193 variants genome-wide with the Illumina HumanOmniExpressExome- 8v1.2 beadchip. We removed SNPs if they deviated from Hardy-Weinberg Equilibrium (HWE) (*p* < 1 × 10^− 7^), had a minor allele frequency < 5%, missingness > 2%, or a heterozygosity rate greater than 3 standard deviations from the mean (PLINK [55, 56]). For mapping eQTLs, we removed SNPs on the Y chromosome. Following QC, we used 608,017 variants for further analysis. We removed one sample with high missingness and outlying heterozygosity rate from further analysis.

### Imputation

We pre-phased the genotypes with SHAPEIT v2 [57]. We imputed missing genotypes and untyped SNPs using Impute2 [58] in 5 Mb chunks against the 1000 Genomes Phase 3 [59] reference panel. To ensure only high-quality genotypes, and to avoid artifacts that can be induced by imputation uncertainty, we removed SNPs with an info score < 1, MAF < 0.05, or HWE *p* < 1 × 10^− 7^ leaving 1,595,793 SNPs for further analysis.

### Interferon status

We classified the interferon (IFN) status of each sample at each time point from the expression of 11 IFN response genes (*HERC5*, *IFI27*, *IRF7*, *ISG15*, *LY6E*, *MX1*, *OAS2*, *OAS3*, *RSAD2*, *USP18*, *GBP5*) using TaqMan Low Density Arrays. These 11 genes were selected by identifying transcripts for which there was both a measureable response to IFN treatment in vitro, as well as differential expression (reduction in expression level) between baseline and visits with clinical improvement in the BOLD study [35]. There is no consensus set of genes to determine the IFN status of SLE patients but these 11 genes do overlap with other published gene sets. For example, 4/11 genes are also used in the 7-gene set defined by McBride et al. [60] and 9/11 genes overlap with the 21-gene set defined by Yao et al. [61].

The first principal component of the expression of the 11-gene set captured 91.7% of the variation (Additional file 1: Figure S25). The distribution of this first principal component is nearly bimodal with good separation (Fig. 2a) and we classified samples as high or low IFN based on this first principal component score. In our dataset, we see excellent correlations (*r*_s_ = 0.86–0.98) between the real-time PCR expression and the RNA-seq expression for these 11 genes (Additional file 1: Figure S26). The first PC of the IFN signature of RNA-seq data is also strongly correlated with the first PC of the IFN signature of real-time PCR (*r*_s_ = 0.96, Additional file 1: Figure S27). IFN status was available for 376 samples from 157 subjects.

### Drug exposure

Samples were assigned as unexposed (placebo or week 0 samples) or drug exposed (week 12 and week 24 samples in the drug groups).

### Free IL-6 protein levels

We determined free IL-6 protein levels from serum using a commercial sandwich ELISA selected for binding only free IL-6. The assay was validated according to FDA biomarker and fit-for purpose guidelines. Free IL-6 protein levels were available for 311 samples from 145 subjects. Since the distribution of IL-6 levels was highly skewed, we ranked samples in order of IL-6 protein levels and included in the model to identify drug-eQTL interactions.

### Statistical analysis

#### eQTL and interaction analysis

In total, 157 patients (with 379 RNA-seq samples) had good quality gene expression and genotyping data for eQTL analysis. All statistical analyses were carried out in R [62].

We defined a *cis* eQTL as the SNP within 250 kb upstream of the GENCODE [51] transcription start site of the gene or 250 kb downstream of the transcription end site. We first applied a linear model for the first available time point (week 0 sample for *n* = 152, week 12 sample for *n* = 5) to identify each eQTL using the first 25 principal components of gene expression and the first 5 principal components of genotyping as covariates.

To select the number of gene expression principal components to include, we counted the number of eQTL genes identified after incrementally increasing the number of principal components accounted for in the model from 0 to 50 by increments of five (Additional file 1: Figure S28). We selected 25 principal components of gene expression to maximize the number of eQTL genes detected while minimizing the number of principal components we corrected for. We included 5 principal components of genotyping to account for the heterogeneity in ethnicity in our cohort (Additional file 1: Figure S29).

SNPs were encoded as 0, 1, and 2 with respect to the number of copies of the minor allele. To adjust for multiple testing during eQTL discovery, we used a stringent Bonferroni corrected *p* value threshold of 8.5 × 10^− 9^ (0.05/5,872,001 tests). The Bonferroni adjustment assumes independence among the tests, and we therefore note that it is a conservative multiple comparisons adjustment.

To map eQTLs using multiple samples for each individual, we applied a random intercept linear mixed model using the first 25 principal components of gene expression and the first 5 principal components of genotyping as covariates and patient as a random effect:

$$ {E}_{i,j}=\theta +{\beta}_{\mathrm{geno}}\bullet {g}_j+\left({\kappa}_i|j\right)+\sum \limits_{l=1}^{25}{\phi}_l\bullet {pc}_{i,l}+\sum \limits_{m=1}^5{\gamma}_m\bullet {pc}_{j,m} $$

where *E*_*i,j*_ is gene expression for the *i*th sample from the *j*th subject, *θ* is the intercept, *β*_geno_ is the effect (eQTL) of the genotype for individual *j* (*g*_*j*_), *(κ*_*i*_*|j)* is the random effect for the *i*th sample from the *j*th subject, *ϕ*_*l*_ is the effect of principal component *l* of gene expression for sample *i (pc*_*i,l*_), and *γ*_*m*_ is the effect of principal component *m* of genotyping for subject *j* (*pc*_*j,m*_).

We fitted the linear mixed models using the lme4 R package [63]. We assumed covariance between samples from the same individual, but did not assume any structure in this covariance.

We used the most significant SNP (with *p* < 8.5 × 10^− 9^) from the 4818 identified eQTL genes to explore eQTL interactions. For each environmental interaction analysis, we further filtered these eQTLs to include only those with at least two individuals homozygous for the minor allele of the SNP being tested in each of the environmental factor groups. For example, we required two of these individuals in each of the drug exposed and drug unexposed groups. To identify eQTL interactions, we added an additional covariate to the model for example drug exposure, and an interaction term between this covariate and the genotype of the SNP:

$$ {E}_{i,j}=\theta +{\beta}_{\mathrm{geno}}\bullet {g}_j+\left({\kappa}_i|j\right)+\sum \limits_{l=1}^{25}{\phi}_l\bullet {pc}_{i,l}+\sum \limits_{m=1}^5{\gamma}_m\bullet {pc}_{j,m}+{\beta}_{\mathrm{drug}}\bullet {d}_i+{\beta}_x\bullet {d}_i\bullet {g}_j $$

where *E*_*i,j*_ is gene expression for the *i*th sample from the *j*th subject, *θ* is the intercept, *β*_geno_ is the effect (eQTL) of the genotype for individual *j* (*g*_*j*_), *(κ*_*i*_*|j)* is the random effect for the *i*th sample from the *j*th subject, *ϕ*_*l*_ is the effect of principal component *l* of gene expression for sample *i* (*pc*_*i,l*_*)*, *γ*_*m*_ is the effect of principal component *m* of genotyping for subject *j* (*pc*_*j,m*_), *β*_drug_ is the effect (differential gene expression) of drug for sample *i* (*d*_*i*_), and *β*_*x*_ is the effect of the drug genotype interaction (*d*_*i*_ *• g*_*j*_).

We determined the significance of the interaction term with a likelihood ratio test.

To rigorously confirm the relative enrichment of eQTL interactions, we shuffled the interaction covariate (for example drug exposure) 1000 times and calculated the number of significant interactions observed in each permutation. Our primary goal for the permutation analysis was to retain the main eQTL effect while examining only the effect of the environmental factor on the interaction. In this study, the main purpose of the covariates included in the model is to ensure the main eQTL effect is found. For IFN high/low status, we shuffled across all samples. For drug interaction permutation analysis, we maintained the number of individuals in the drug group and the number of samples with exposure to drug. We calculated a *q* value for each interaction using the *q* value package [36]. Additional file 1: Figure S30 shows the observed versus the expected *p* values for the interaction analyses.

The expression of the majority of genes followed a normal distribution (Additional file 1: Figure S31) but to assess whether non-normality could be causing an inflation of our test statistic, we repeated the identification of eQTLs and eQTL interactions following the standard normal transformation. We transformed the expression values of each gene to their respective quantiles of a normal distribution using the qqnorm function in R, breaking any ties (for example expression levels of zero in some individuals) randomly.

#### Concordance with an eQTL study in healthy individuals

In the SLE cohort, we classified 4818 *cis* eQTL genes (*p* < 8.5 × 10^− 9^). The *z-*score for the most associated SNP for each of these genes was compared to the *z-*score from a previously published eQTL dataset from whole blood from 2166 healthy individuals [11]. 4113/4818 SNP-gene pairs (85.4%) were also reported in the BIOS dataset (FDR < 0.05). After removing 301 SNPs, which could not be mapped to a strand, 3770/3812 (98.9%) had a *z-*score (eQTL effect) in a consistent direction.

#### Magnifiers and dampeners

An eQTL interaction can either magnify or dampen the original eQTL effect. We multiplied the interaction *z*-score by the sign of the original eQTL effect (genotype beta) and defined magnifiers as interactions with an adjusted *z*-score > 0 and dampeners as interactions with an adjusted *z-*score < 0.

#### Differential gene expression analysis

To identify differentially expressed genes following drug exposure (unexposed or exposed), we applied a random intercept linear mixed model with patient as a random effect. We calculated a *q* value using the *q* value package [36].

#### Drug exposure score

We assigned a drug exposure score to each sample. We calculated a score for each gene (see equation below) and then averaged across the seven drug-eQTL genes (FDR < 0.1) to give the final drug exposure score.

$$ \mathrm{Drug}\ \mathrm{exposure}\ \mathrm{score}\ \mathrm{for}\ \mathrm{gene}=\frac{1}{2}{\left(\frac{G-{G}_{\mathrm{Unexp}}}{\mathrm{SE}}\right)}^2-\frac{1}{2}{\left(\frac{G-{G}_{\mathrm{Exp}}}{SE}\right)}^2 $$

where *G* is gene expression for a given sample, *G*_Unexp_ is predicted mean gene expression for unexposed samples of the relevant SNP genotype, *G*_Exp_ is predicted mean gene expression for exposed samples of the relevant SNP genotype, and SE is standard error for the intercept term of the model (unexposed expression for genotype 0).

#### HOMER analysis for transcription factor binding motif enrichment

We used the HOMER software suite [37] to look for enrichment of transcription factor binding motifs in the 210 IFN-eQTL interactions (FDR < 0.2) and the 72 drug-eQTL interactions (FDR < 0.2). Each eQTL interaction was identified using the most highly associated SNP for that eQTL. However, as this SNP is not necessarily the functional SNP, we additionally considered all those with an *r*^2^ ≥ 0.8 in the 1000 Genomes European population [59] within the *cis* eQTL window. We defined our motif search window as 20 bp on either side of each SNP (i.e., 41 bp wide).

For each environmental factor, we divided the eQTL interactions into magnifiers or dampeners and conducted two separate HOMER analyses: the proportion of magnifying eQTL interaction sequences with a motif compared to the proportion of dampening interaction sequences with a motif and vice versa. HOMER reported the transcription factor motifs that were significantly enriched in one category of interactions relative to the other. Motifs were plotted using the SeqLogo R library [64].

We determined permutation *p* values for enrichment of the ISRE and IRF4 transcription factor binding sites as follows. For ISRE, the motif is interrupted by interaction SNPs (or SNPs in LD) corresponding to 11 magnifying genes and 1 dampening gene. We permuted which genes were labeled as magnifiers or dampeners 100,000 times and counted the number of genes in each category with an ISRE motif interrupted. We found 1855 occurrences from 100,000 trials with at least 11 magnifying genes (*p* < 0.019). For IRF4, the motif is interrupted by SNPs corresponding to 9 magnifying genes and 4 dampening genes. Using the same permutation approach, we found 5801 occurrences from 100,000 trials with at least 9 magnifying genes (*p* < 0.058).

#### Cell counts

We collected 4 ml whole blood in sodium heparin vacutainers for cytometry analysis at weeks 0, 12, and 24. Samples were subjected to flow cytometry for T cell and B cell immunophenotyping (Additional file 1: Figure S32). We counted T (CD3+) and B (CD19+) cells as a percentage of lymphocytes (CD45+, SSC-small) because of the abnormal distribution of lymphocytes observed in SLE [65]. These counts are therefore inversely correlated (*r*_s_ = − 0.65, Additional file 1: Figure S33). FACS data were available for 320 samples from 152 subjects.

We used CIBERSORT [40] to deconvolute proportions of cell types from the RNA-seq data. We used the LM22 database from CIBERSORT which contains cell signatures for 22 cell types and grouped these into nine representative cell types (eosinophils, neutrophils, B cells, T cells, natural killer cells, macrophages, dendritic cells, mast cells, and monocytes).

## Additional files

Additional file 1: Supplementary Figure S1-S33. (PDF 8033 kb)

Additional file 2: Supplementary Tables S1-S6. (XLSX 6624 kb)

Additional file 3: Review history. (DOCX 841 kb)

Additional file 4: Supplementary Table S7. (PDF 148 kb)
