# Supplementary material for: Discovering in vivo cytokine-eQTL interactions from a lupus clinical trial
Source: Genome Biol. 2018 Oct 19;19:168. doi: 10.1186/s13059-018-1560-8 (PMC6195724; doi:10.1186/s13059-018-1560-8)
Supplement: Supplementary file 1 — Supplementary Figure S1-S33. (PDF 8033 kb) [file 13059_2018_1560_MOESM1_ESM.pdf]

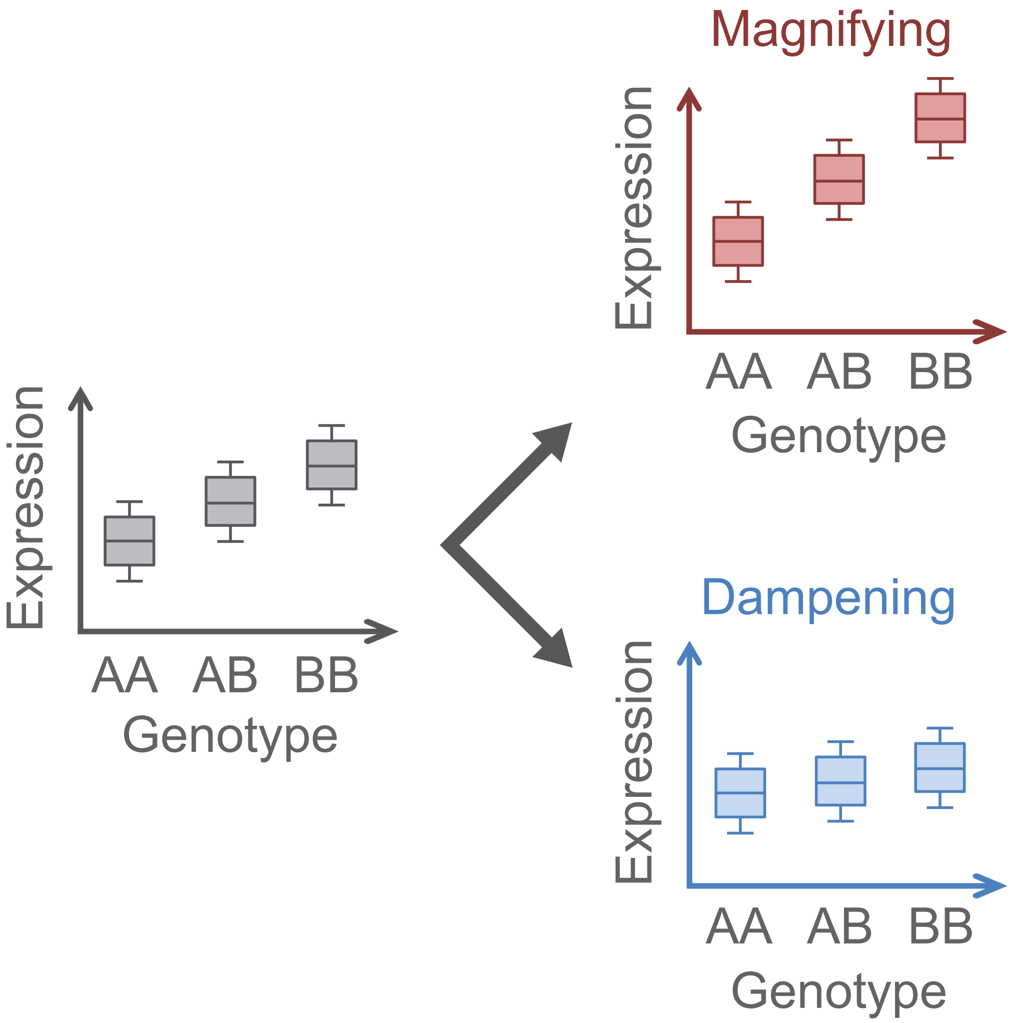


**Figure S1: Effect of the interaction on the original eQTL effect.** eQTL interactions can be divided into magnifiers, where environmental exposure increases the size of the eQTL effect, and dampeners where environmental exposure decreases the eQTL effect.

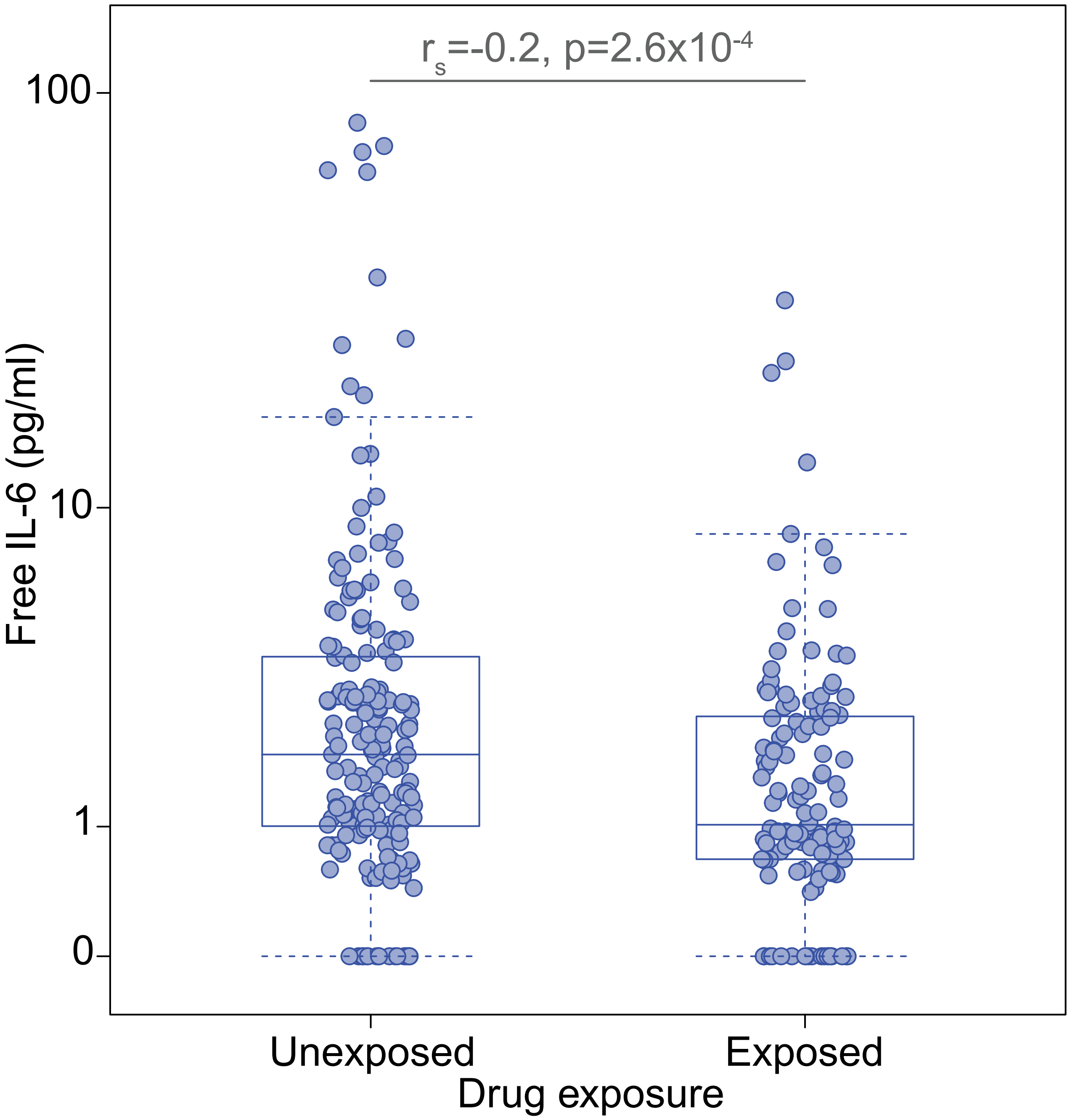


**Figure S2: Relationship between free IL-6 protein levels and drug exposure.** Free IL-6 protein levels plotted with respect to the drug exposure of the sample.

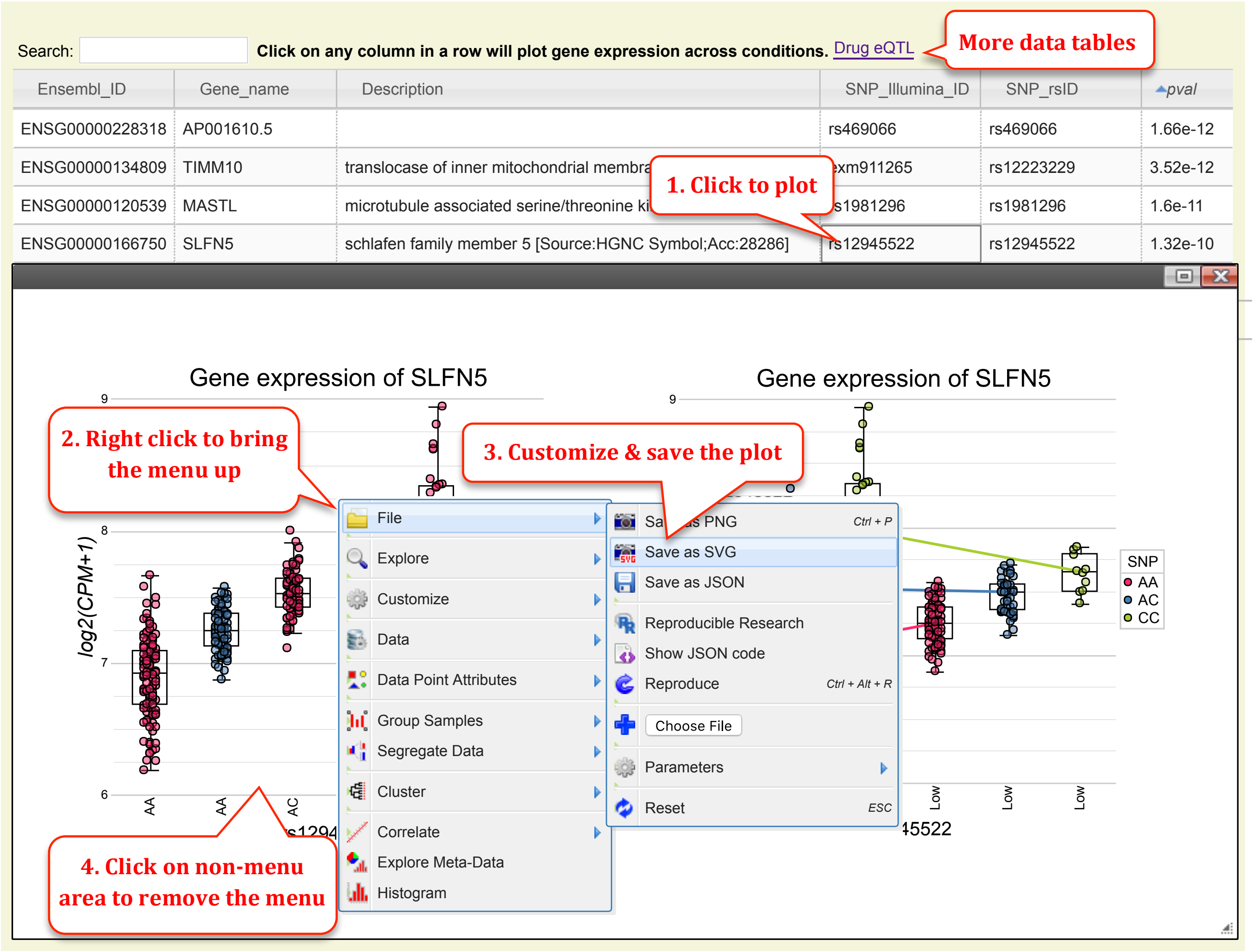


**Figure S3: Interactive eQTL interaction visualisation tool.** Step-by-step user guide for exploring eQTL interactions through the interactive visualisation tool (`http://baohongz.github.io/Lupus_eQTL`).

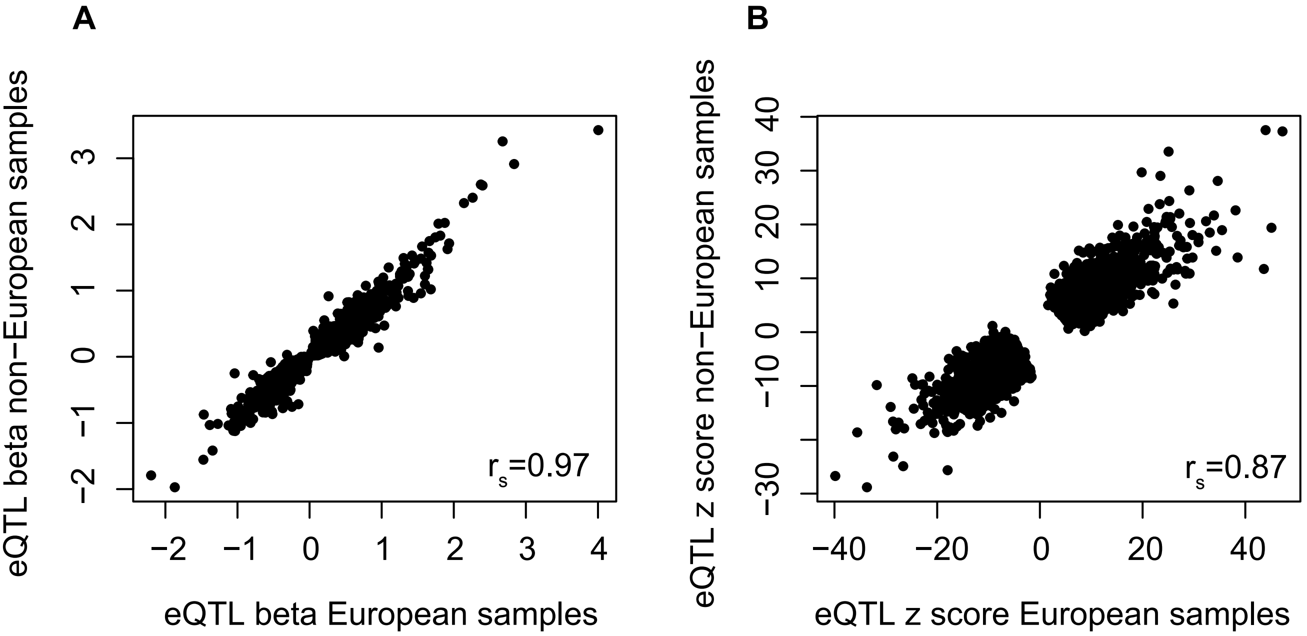


**Figure S4: Comparison of eQTL effects between ethnicities.** Correlations of A) eQTL betas and B) eQTL z scores between European and non-European samples. Restricted to the 4,680 gene SNP pairs with a significant eQTL ($p<8.5x10^{-9}$) across the whole cohort and a minor allele frequency >0.05 in both the European (90 individuals, 226 samples) and non-European (67 individuals, 153 samples) samples.

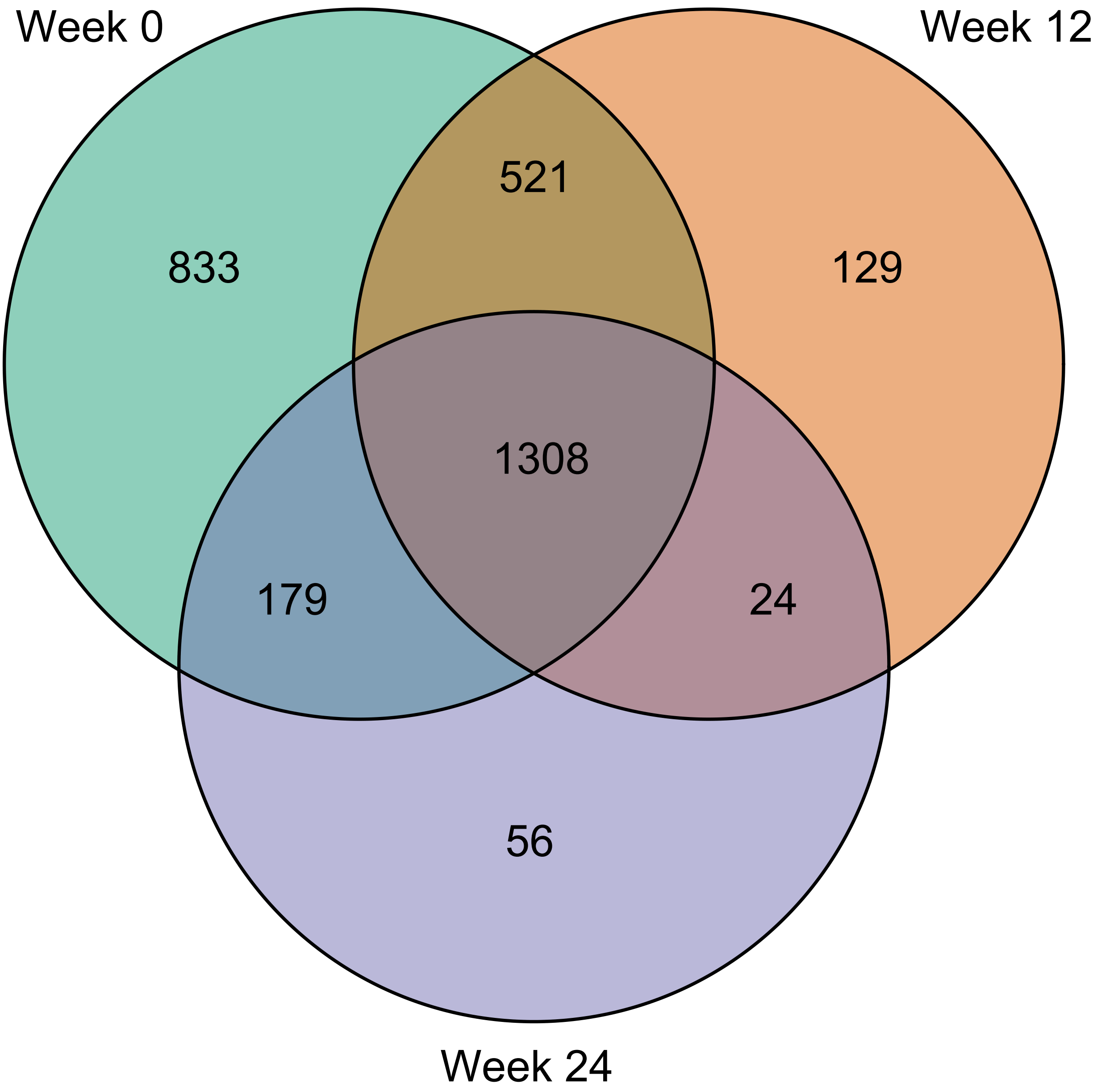


**Figure S5: Overlap of eQTL genes mapped from separate RNA-seq time points.** Genes with a *cis* eQTL ($p<8.5\times10^{-9}$) identified by mapping eQTL separately for each time point (week 0 n=152, week 12 n=121, week 24 n=106) using a linear model.

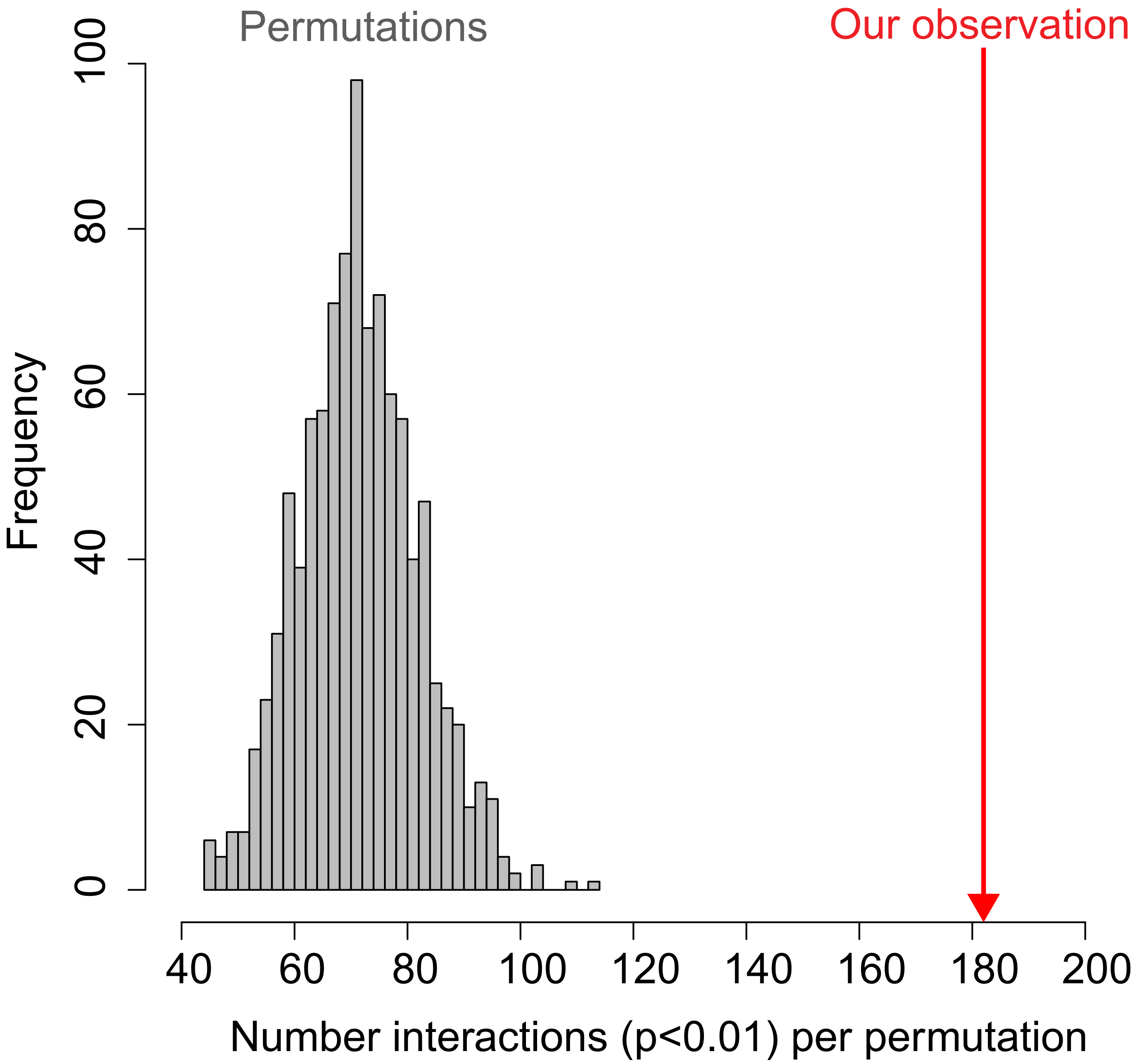


**Figure S6: Distribution of IFN-eQTL interactions from permutations.** The number of significant IFN-eQTL interactions (p<0.01) from each of 1,000 permutations of IFN status.

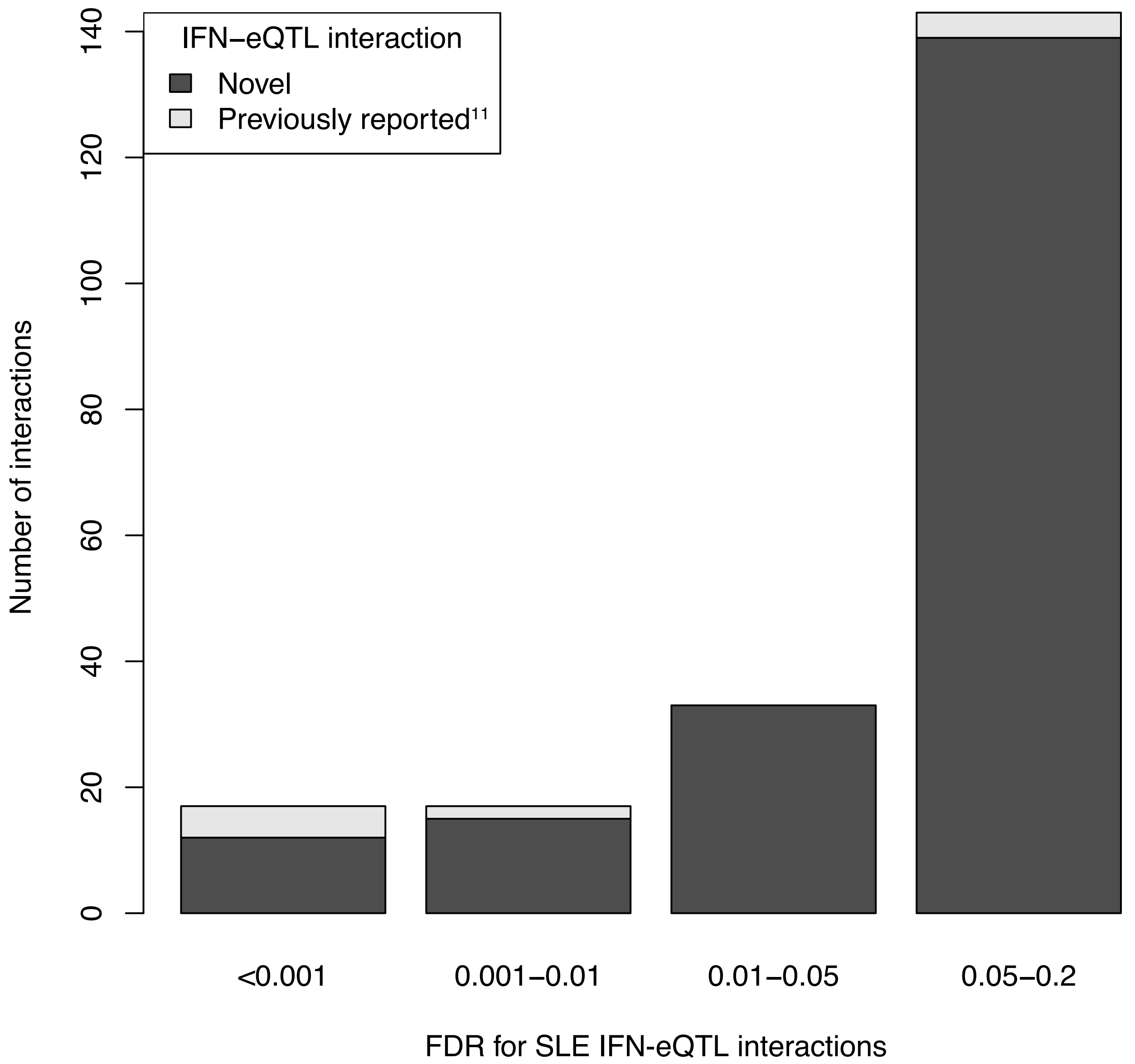


**Figure S7: Number of novel IFN-eQTL interactions at different FDR thresholds.** The 210 IFN-eQTL interactions with FDR<0.2 are binned by FDR. For each bin the number of IFN-eQTL interactions that have been previously reported in the BIOS cohort[11] and those that are novel are shown.

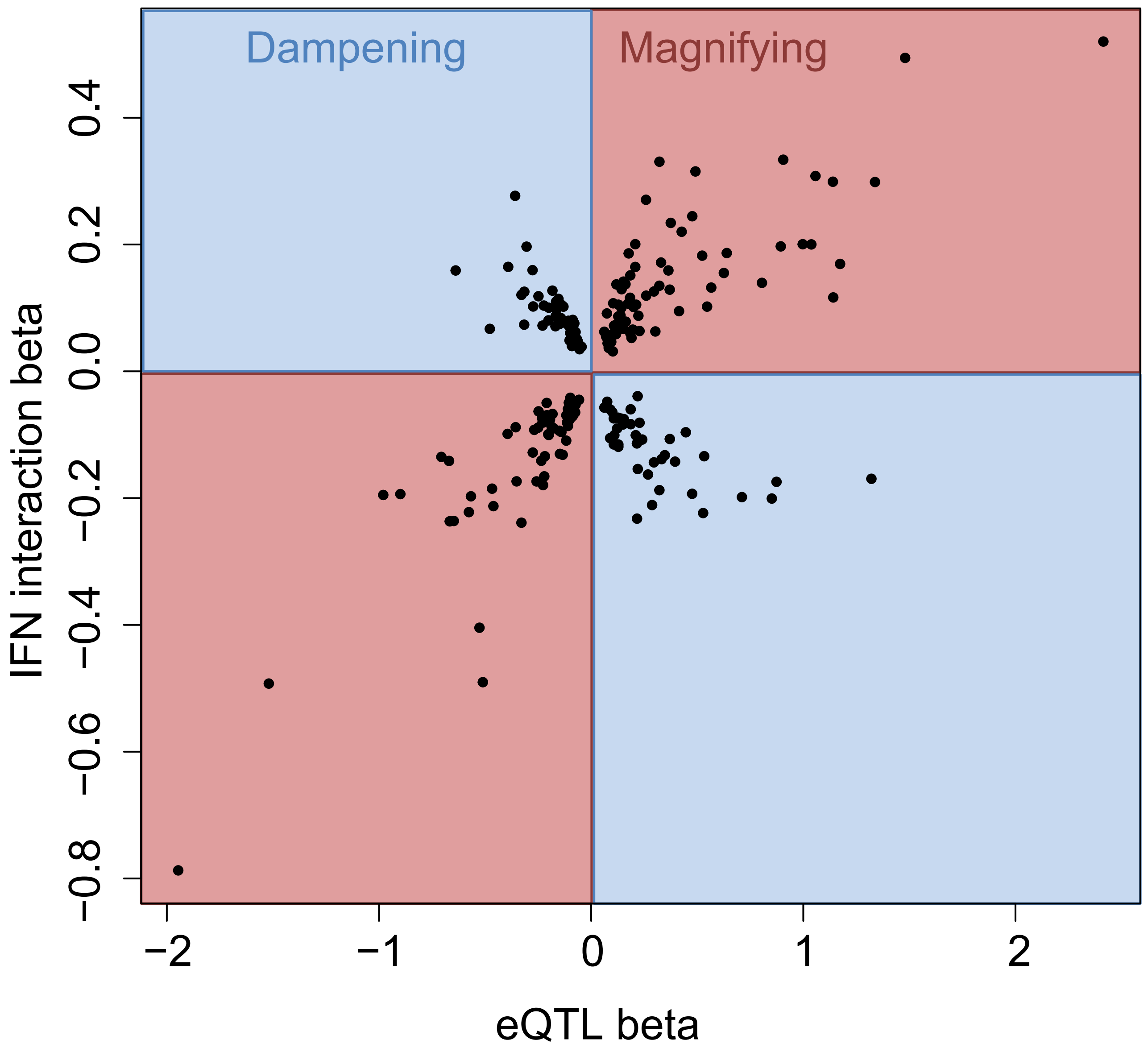


**Figure S8: Effect of the IFN interaction on the original eQTL effect.** For each of the 210 IFN-eQTL interactions with FDR<0.2, the IFN interaction effect is plotted against the original eQTL effect. IFN-eQTL interactions are divided into magnifying or dampening depending on the direction of the original eQTL effect and the direction of the interaction.

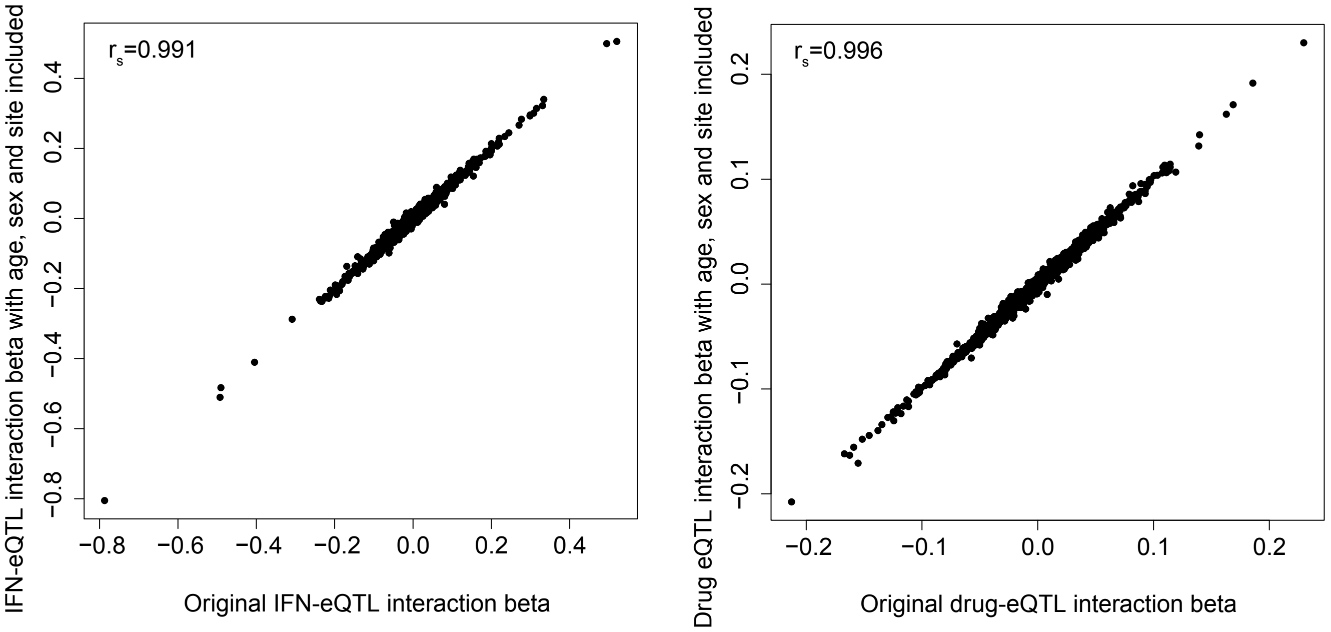


**Figure S9: Effect of age, sex and recruitment site on IFN-eQTL interactions.** Correlation between the IFN and drug-eQTL interaction effects with and without age, sex and recruitment site included in the model

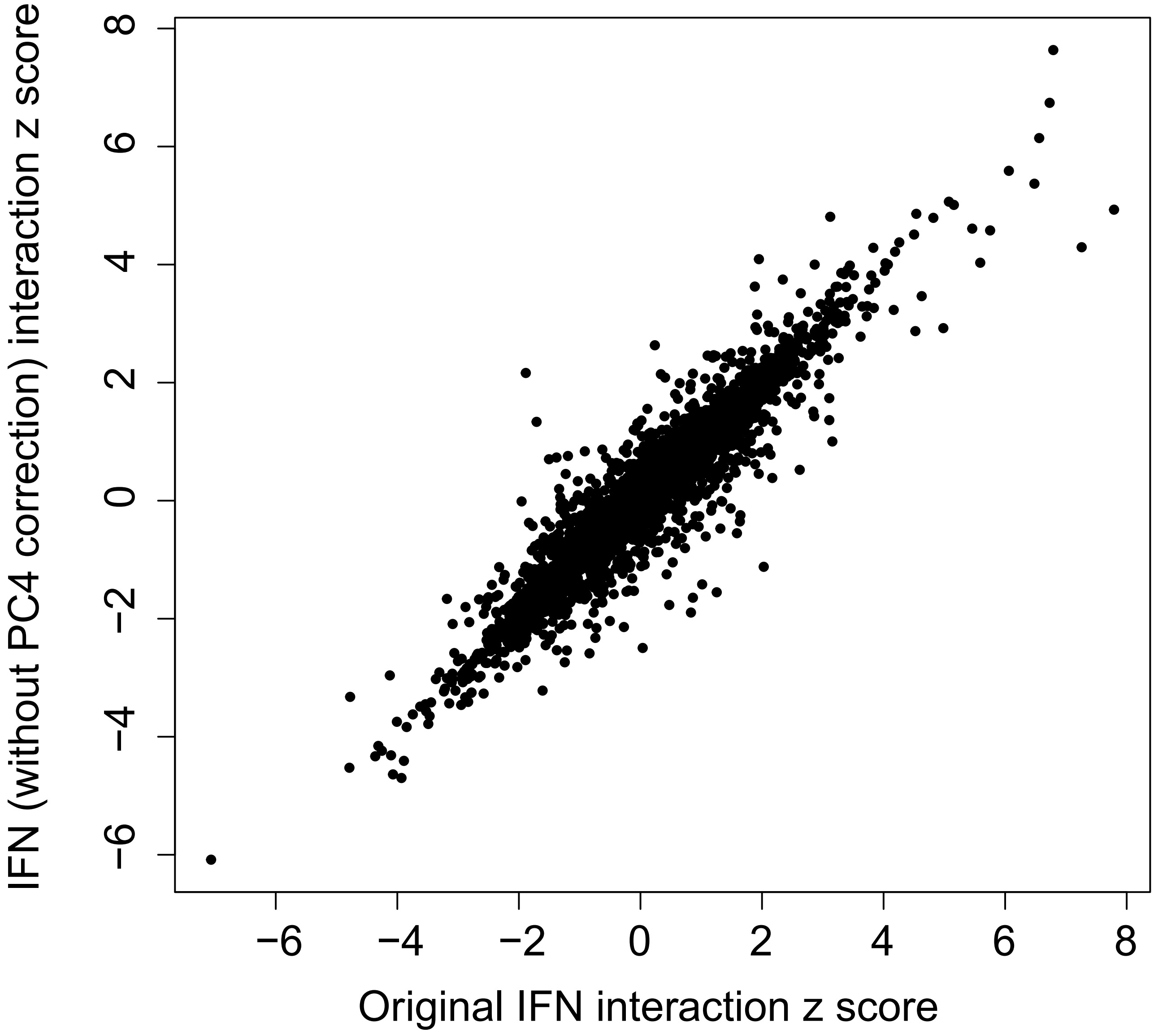


**Figure S10: Effect of 4 $^{th}$ principal component adjustment on IFN-eQTL interactions.** The fourth principal component of gene expression is correlated with IFN status ($r_s$=-0.7). Using all 4,818 eQTLs tested for an eQTL interaction with IFN, the original z scores (with correction for principal component 4) are highly correlated with the z scores without correcting for principal component 4 ($r_s$=0.94).

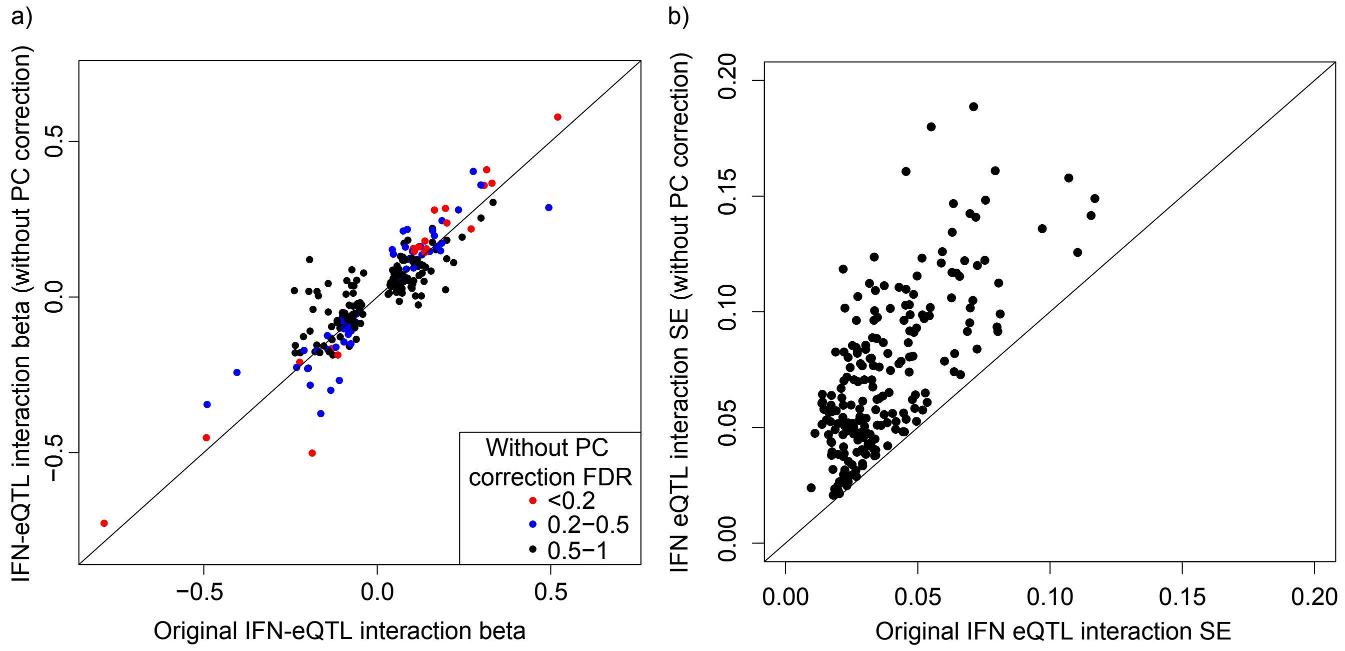


**Figure S11: Effect of principal component adjustment on IFN-eQTL interactions.** a) The IFN-eQTL interaction effects are highly correlated with and without correcting for gene expression principal components ($r_s$=0.88). The color represents the FDR of IFN-eQTL interaction without correcting for gene expression principal components. b) Correction for gene expression principal components reduces the standard error of the IFN-eQTL interactions.

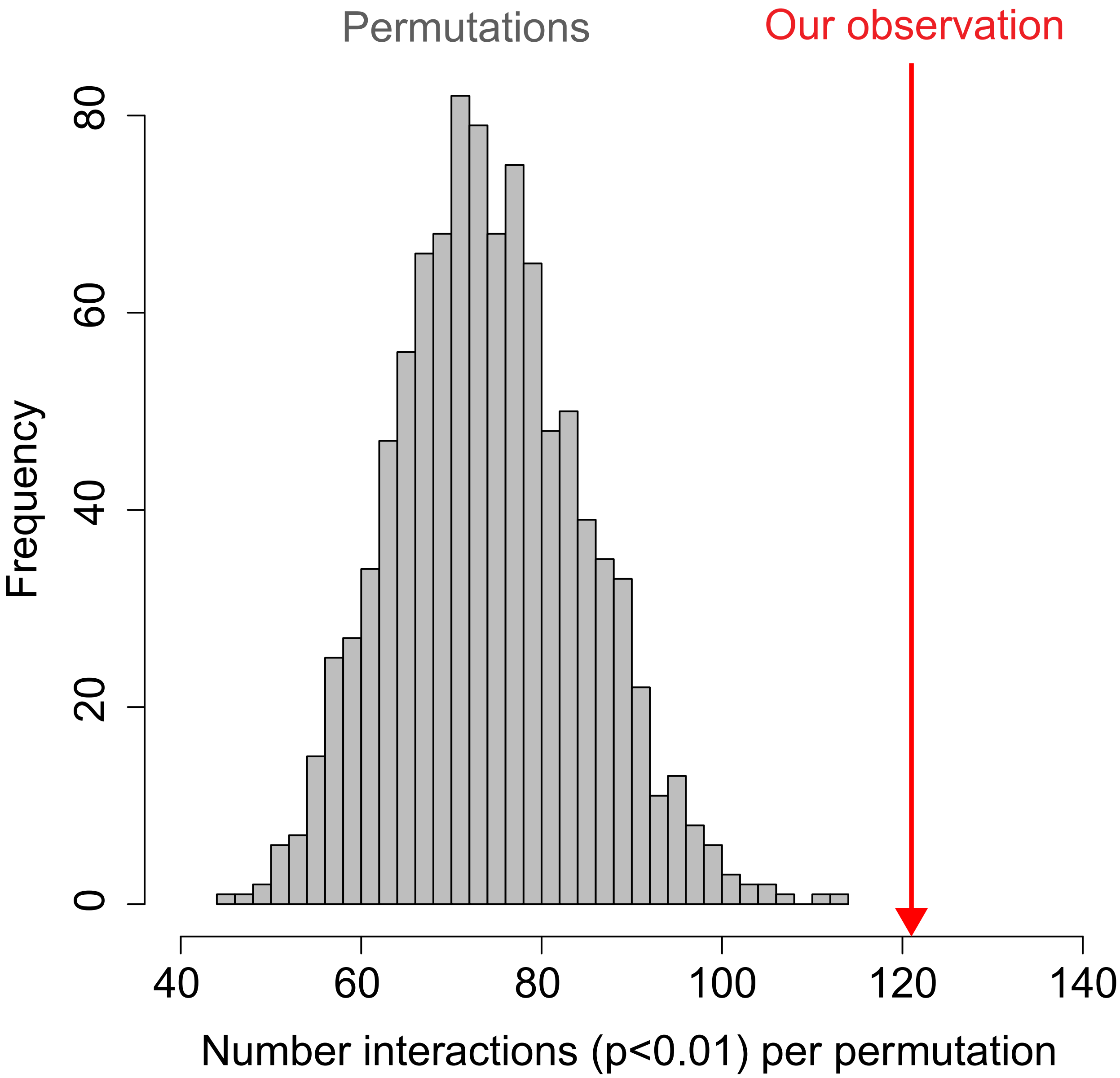


**Figure S12: Distribution of drug-eQTL interactions from permutations.** The number of significant drug-eQTL interactions (p<0.01) from each of 1,000 permutations of drug exposure status.

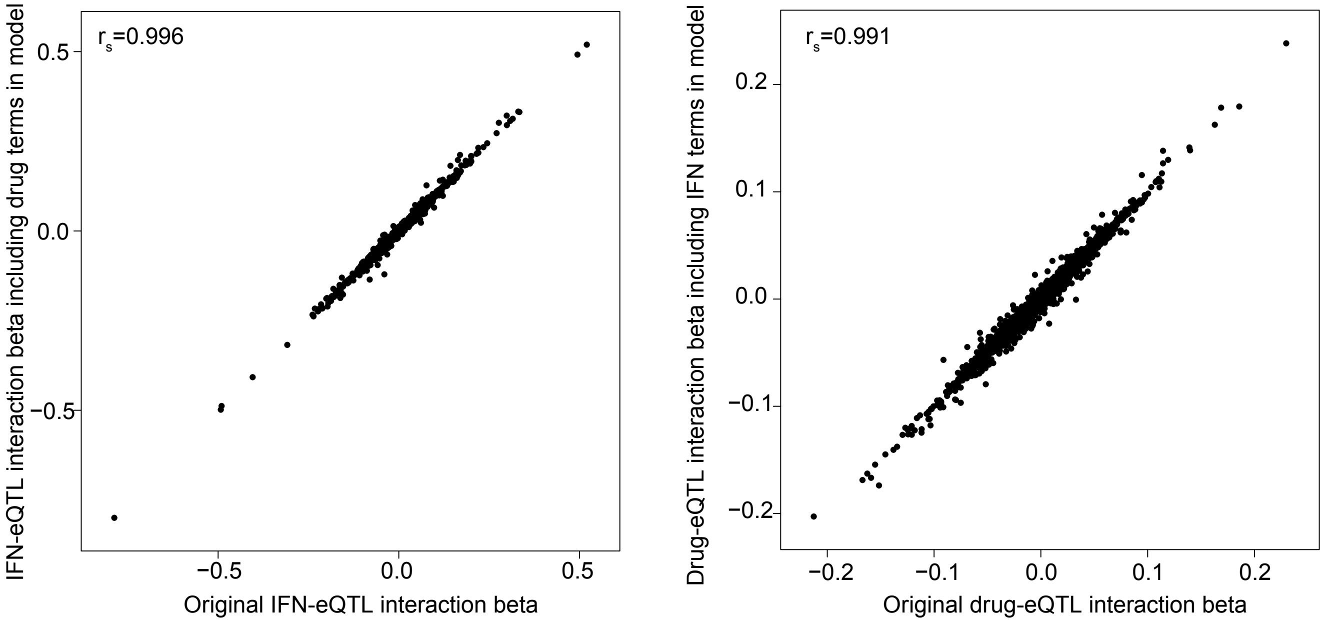


**Figure S13: Effect of including additional differential and interaction terms in the model.** For both the IFN and drug-eQTL interactions, the analysis was repeated using a full model including IFN, IFN interaction, drug and drug interaction terms. The interaction betas from the full model are highly concordant with the original analysis.

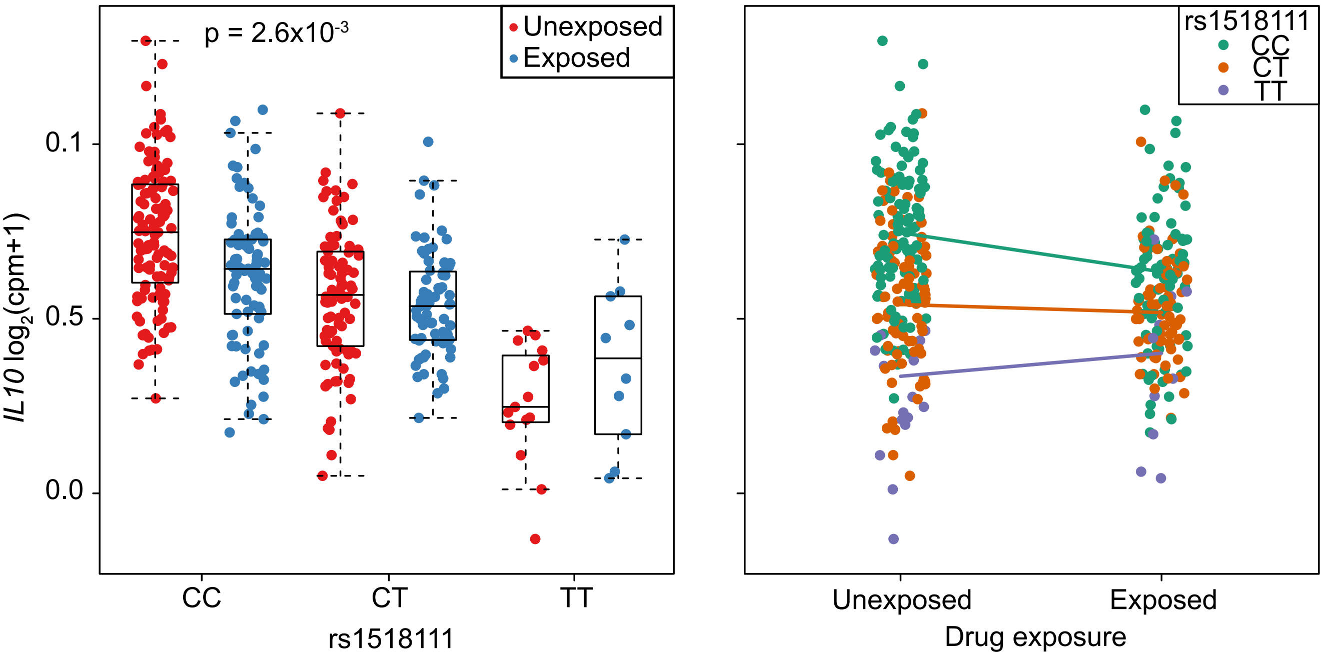


**Figure S14: Drug-eQTL interaction for *IL10*.** Drug exposure interaction with the *IL10* eQTL plotted with respect to rs1518111 genotype (left) and drug exposure (right). The eQTL is dampened in drug exposed samples.

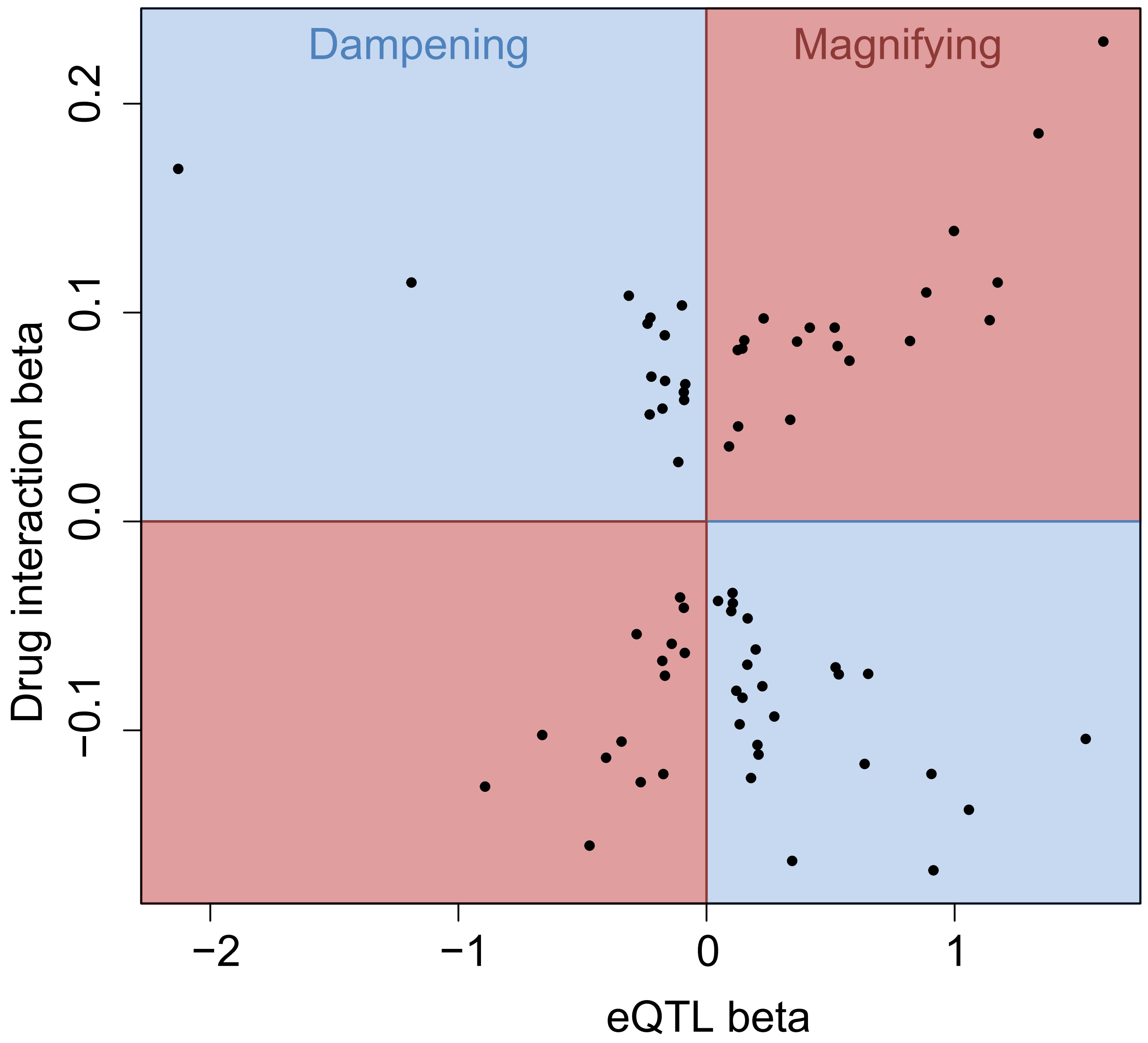


**Figure S15: Effect of the drug interaction on the original eQTL effect.** For each of the 72 drug-eQTL interactions with FDR<0.2, the drug interaction effect is plotted against the original eQTL effect. Drug-eQTL interactions are divided into magnifying or dampening depending on the direction of the original eQTL effect and the direction of the interaction.

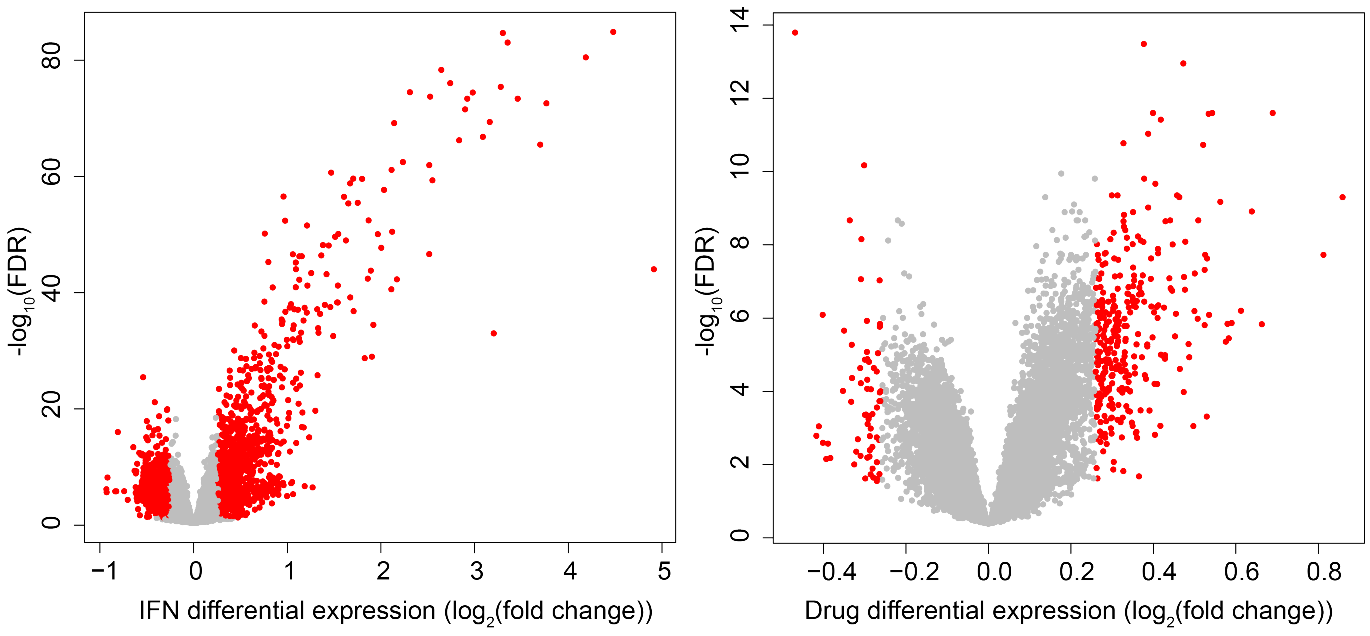


**Figure S16: Differential expression.** Each point represents one of the 20,253 transcripts tested for differential expression. Red points represent transcripts with FDR<0.05 and fold change>1.2.

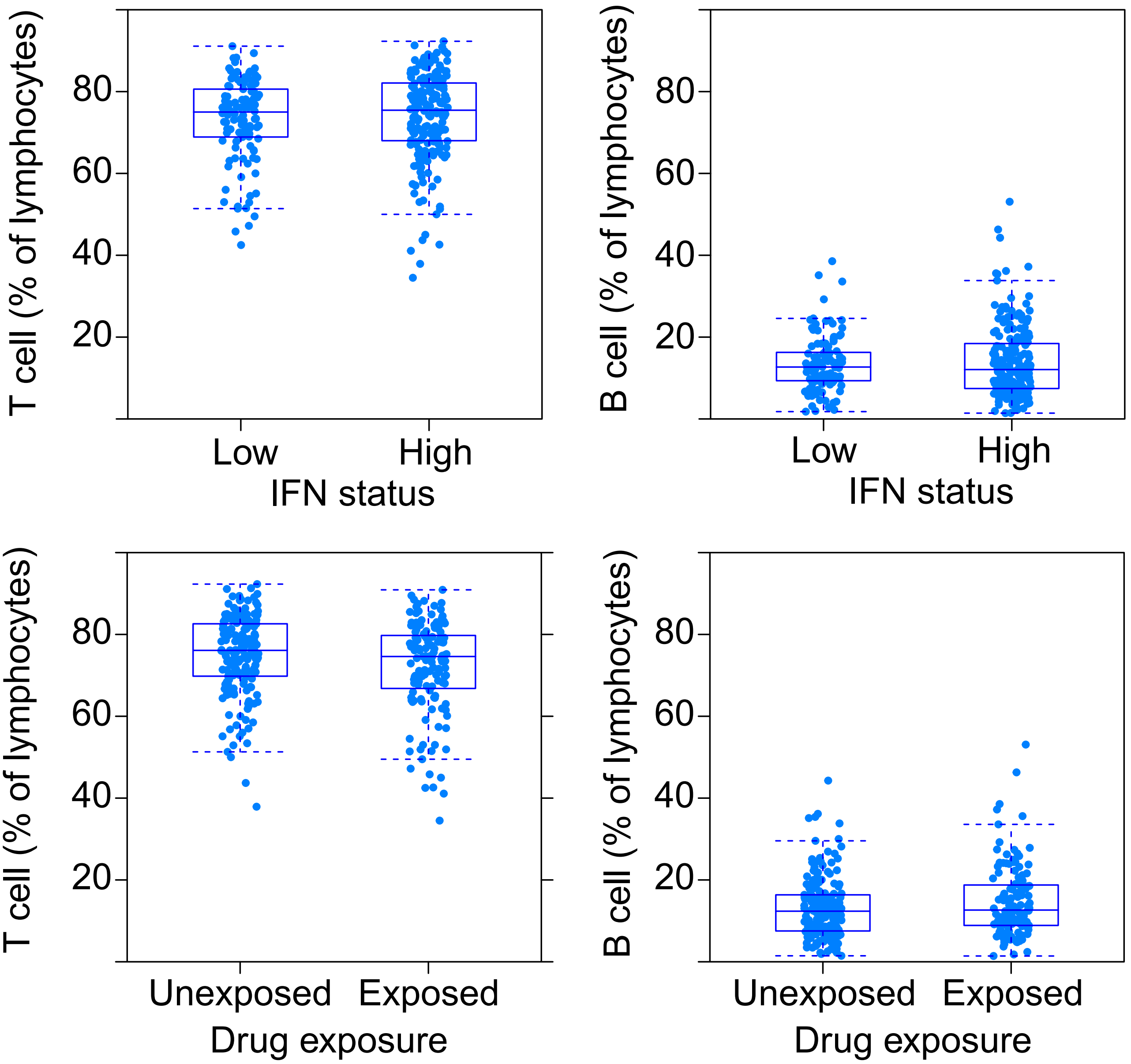


**Figure S17: T and B cell proportions across IFN status and drug exposure.** T and B cell proportions do not correlate with either IFN status or drug exposure.

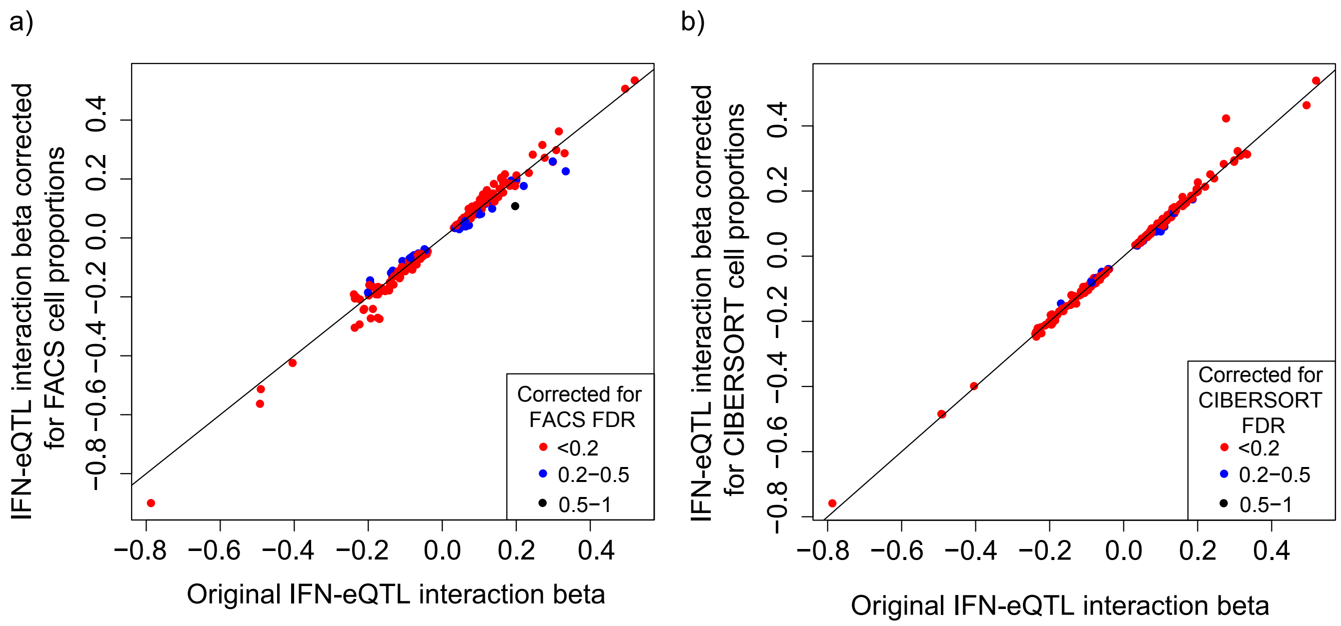


**Figure S18: Effect of cell proportion correction on IFN-eQTL interactions.** The IFN-eQTL interaction effects are highly correlated with and without correcting for cell proportions. a) Correlation after correcting for T and B cell proportions from FACS data ($r_s$=0.99). b) Correlation after correcting for 9 hematopoietic populations inferred from RNA-seq data using CIBERSORT ($r_s$=0.998). The color represents the FDR of the IFN-eQTL interaction after the relevant cell proportion correction.

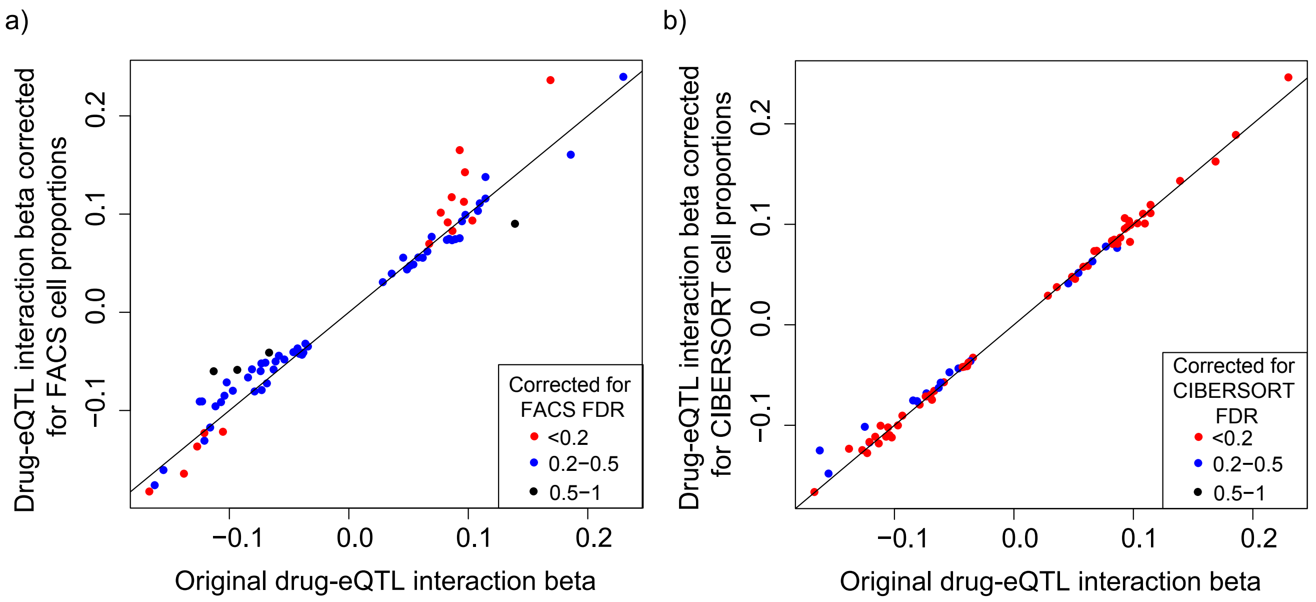


**Figure S19: Effect of cell proportion correction on drug-eQTL interactions.** The drug-eQTL interaction effects are highly correlated with and without correcting for cell proportions. a) Correlation after correcting for T and B cell proportions from FACS data ($r_s$=0.98). b) Correlation after correcting for 9 hematopoietic populations inferred from RNA-seq data using CIBERSORT ($r_s$=0.993). The color represents the FDR of the drug-eQTL interaction after the relevant cell proportion correction.

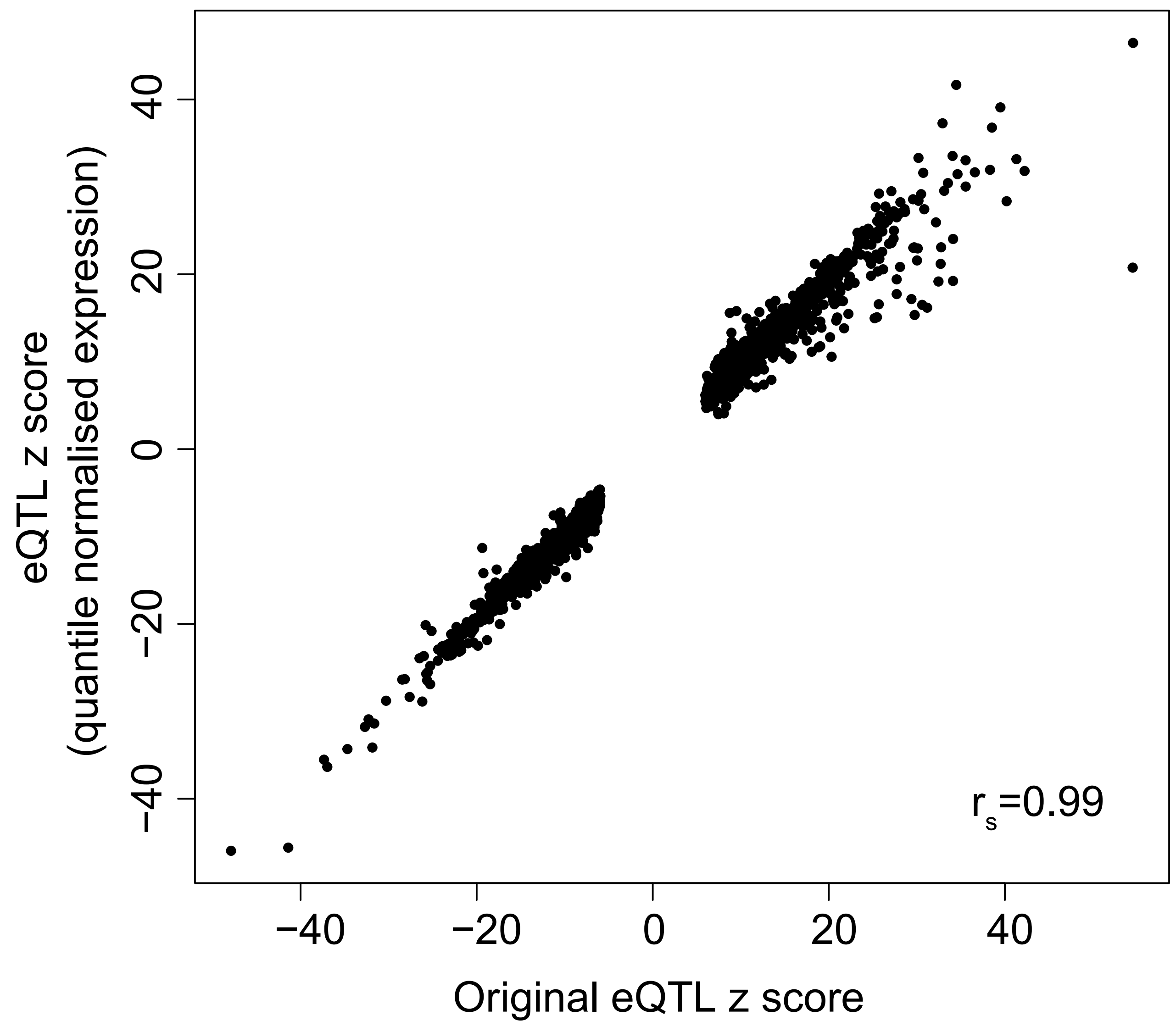


**Figure S20: Comparison of eQTL effects after standard normal transformation.** Correlation between the z score of the original eQTL and the z score when gene expression is quantile normalised. Restricted to the 4,818 genes with a significant eQTL in the original analysis.

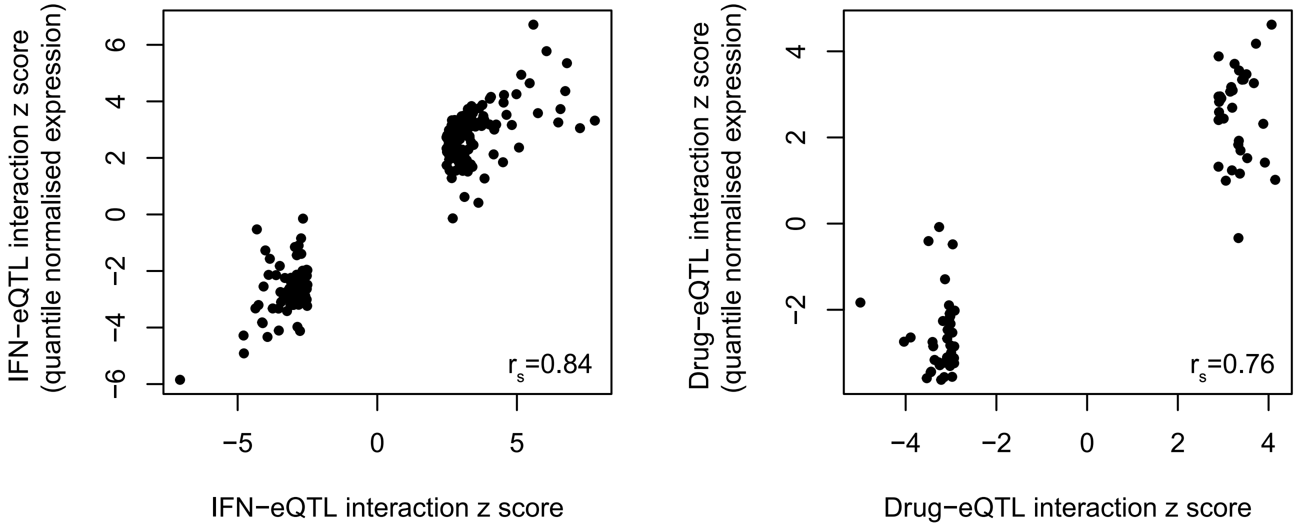


**Figure S21: Comparison of eQTL interaction effects after standard normal transformation.** Correlation between the z score of the original eQTL interaction and the z score when gene expression is quantile normalised. Restricted to the genes with a significant eQTL interaction in the original analysis (FDR<0.2). IFN-eQTL interactions n=210. Drug-eQTL interactions n=72.

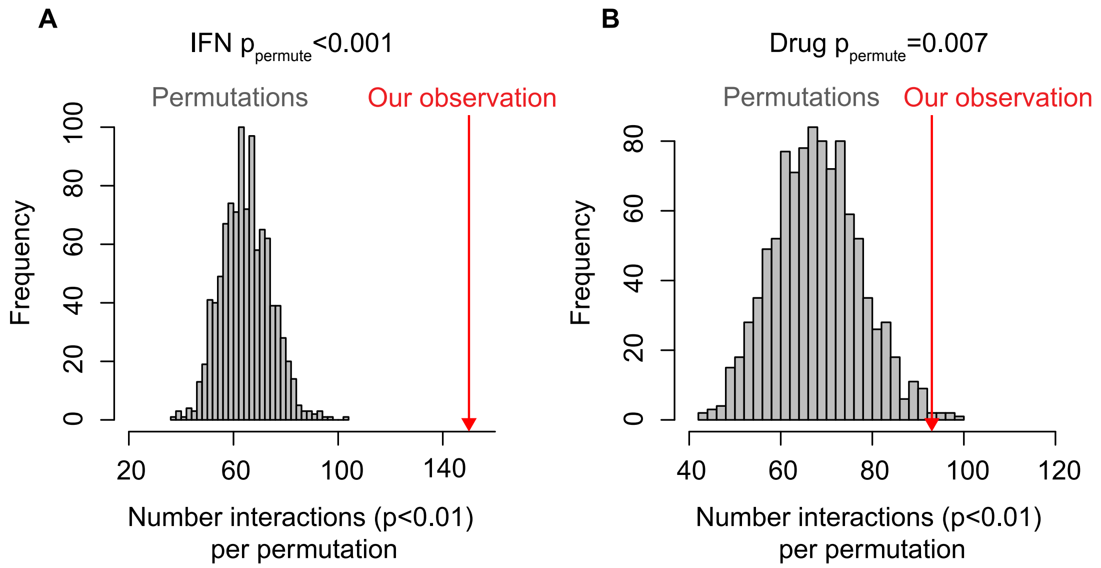


**Figure S22: Distribution of eQTL interactions following standard normal transformation.** Number of significant interactions ($p<0.01$) from 1,000 permutations when gene expression is quantile normalised. A) There were no instances of at least 150 significant IFN-eQTL interactions in our permutations. B) There were 7 instances of at least 93 significant drug-eQTL interactions in our permutations.

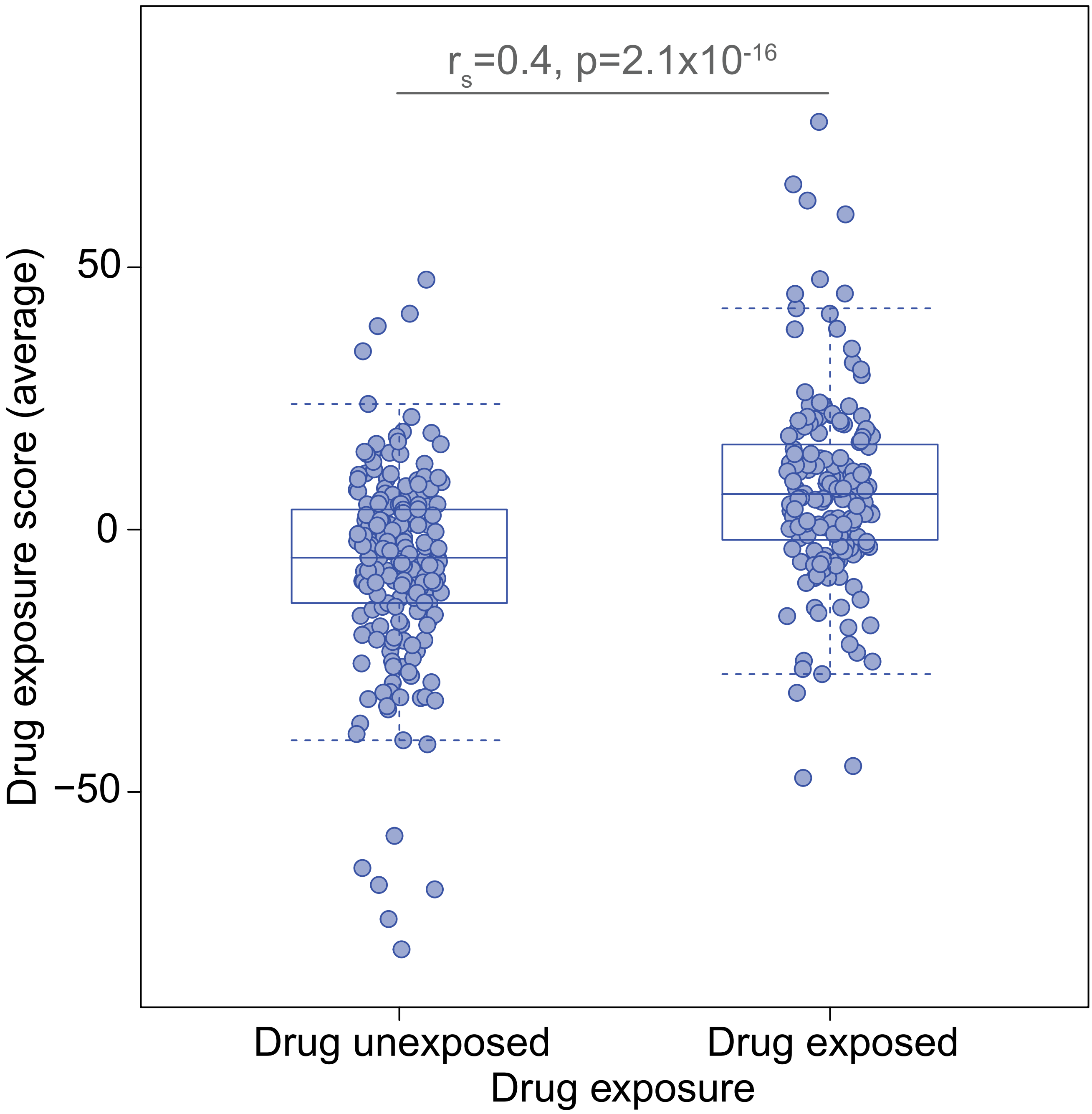


**Figure S23: Relationship between drug exposure and drug exposure score.** Drug exposure score calculated from 7 drug-eQTL interactions with FDR<0.1 plotted with respect to the drug exposure of the sample.

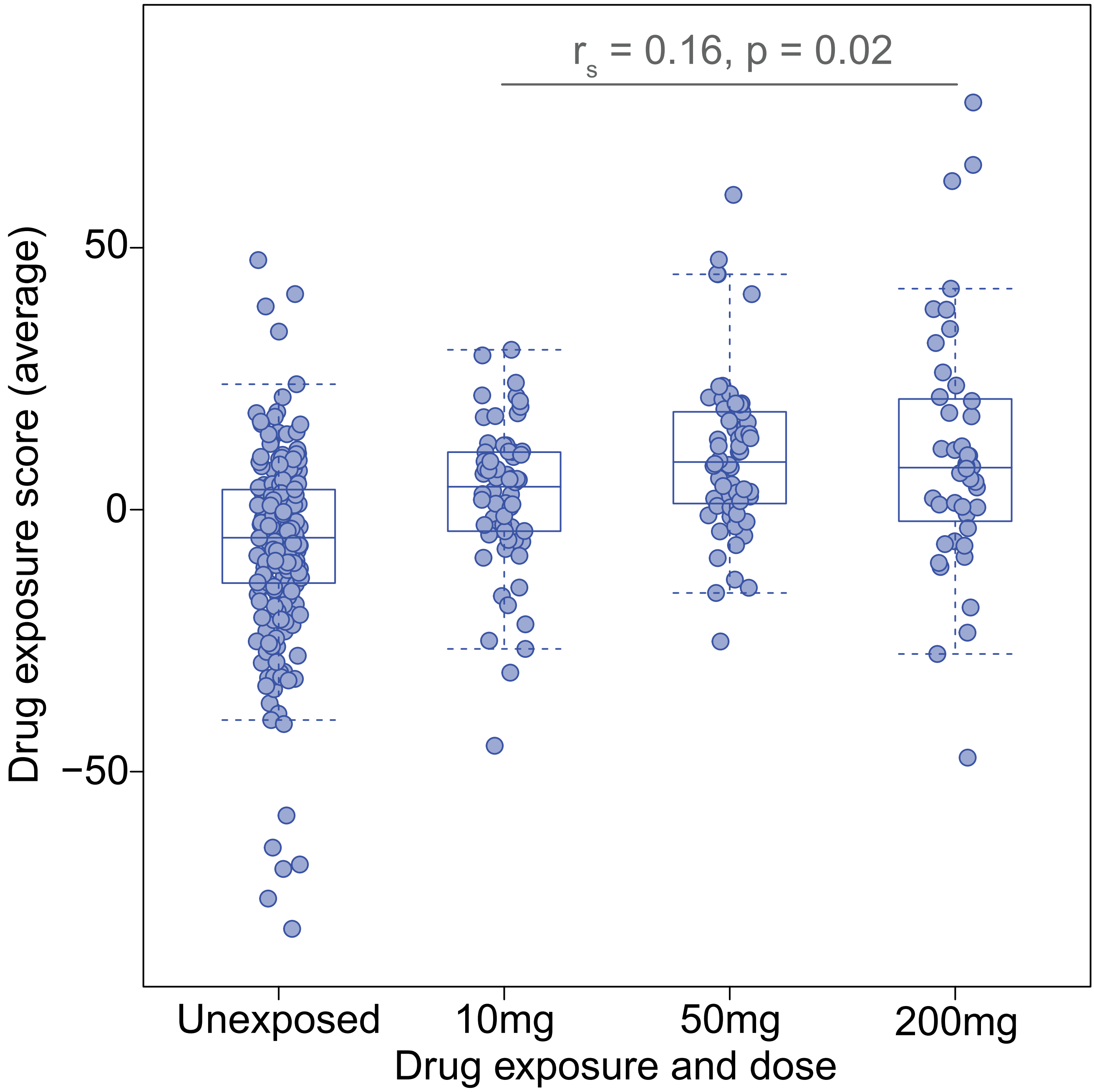


**Figure S24: Relationship between drug dose exposure and drug exposure score.** Drug exposure score calculated from 7 drug-eQTL interactions with FDR<0.1 plotted with respect to the drug dose exposure of the sample.

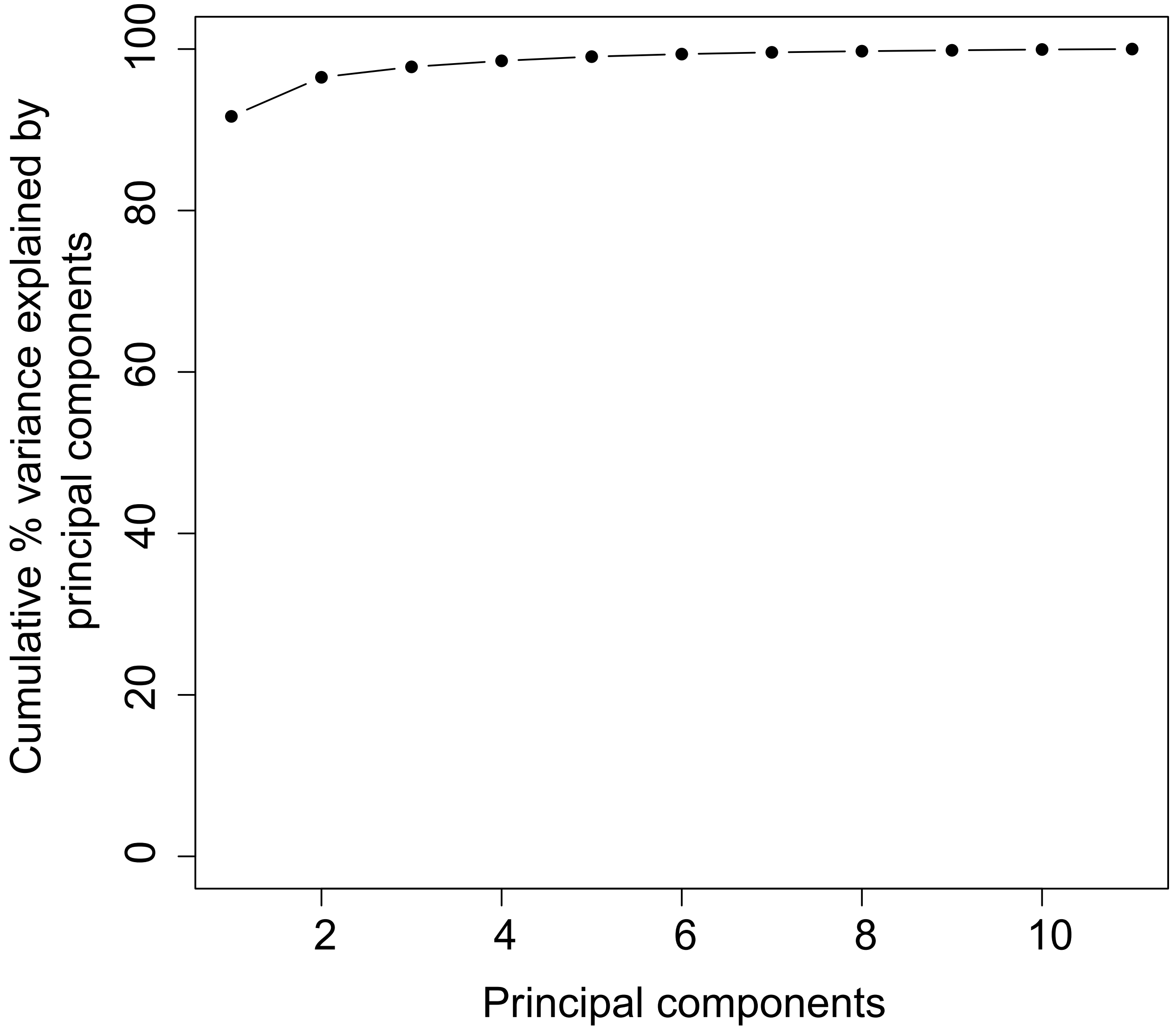


**Figure S25: Cumulative variance explained by principal components of IFN status gene-set.** The IFN status of each sample was determined using the real-time PCR expression of 11 IFN response genes (*HERC5, IFI27, IRF7, ISG15, LY6E, MX1, OAS2, OAS3, RSAD2, USP18, GBP5*). The cumulative percentage variance explained by the principal components of these 11 genes is plotted. The first principal component captures 91.7% of the variation.

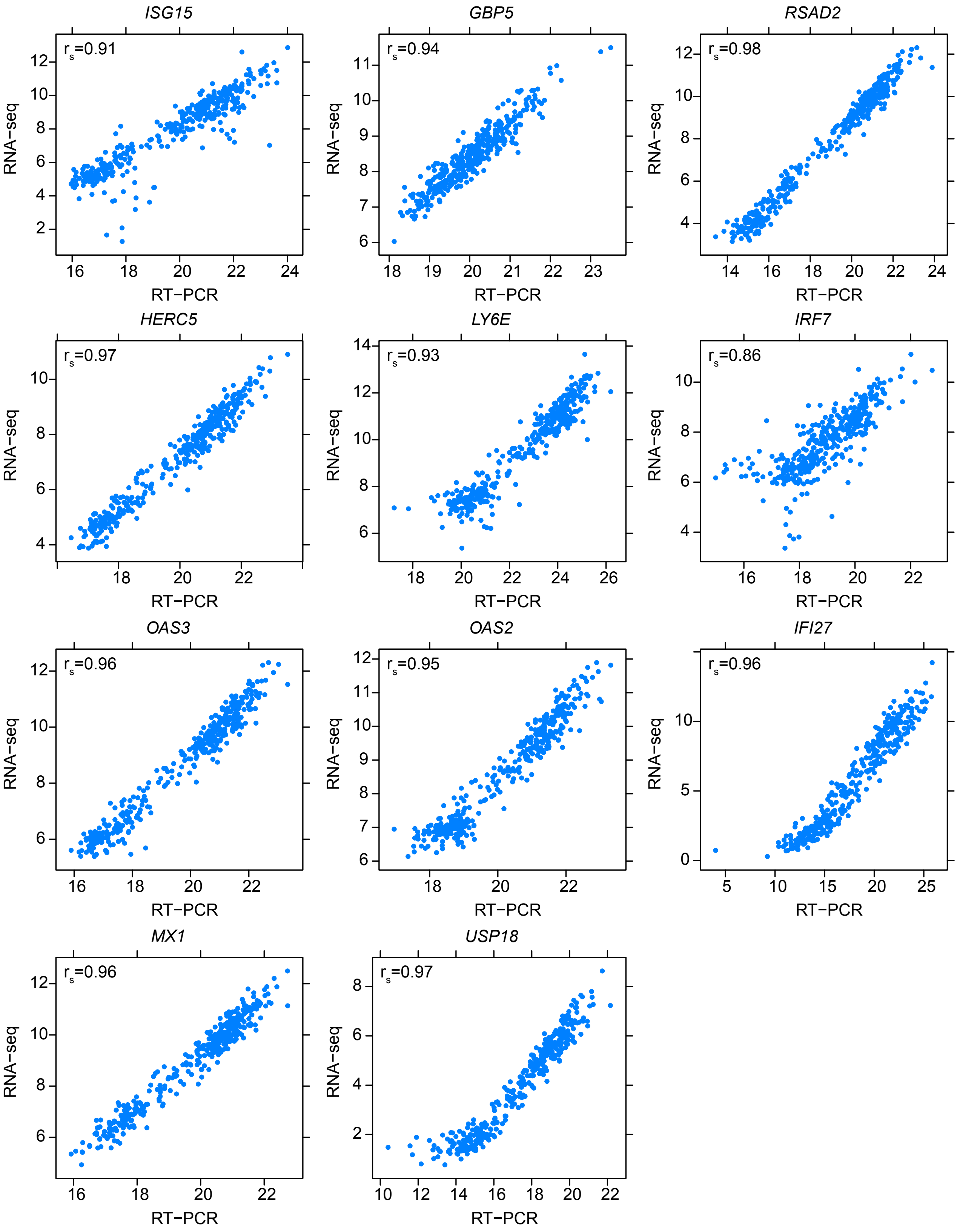


**Figure S26: Correlation of IFN status 11 gene-set between real-time PCR and RNA-seq.**

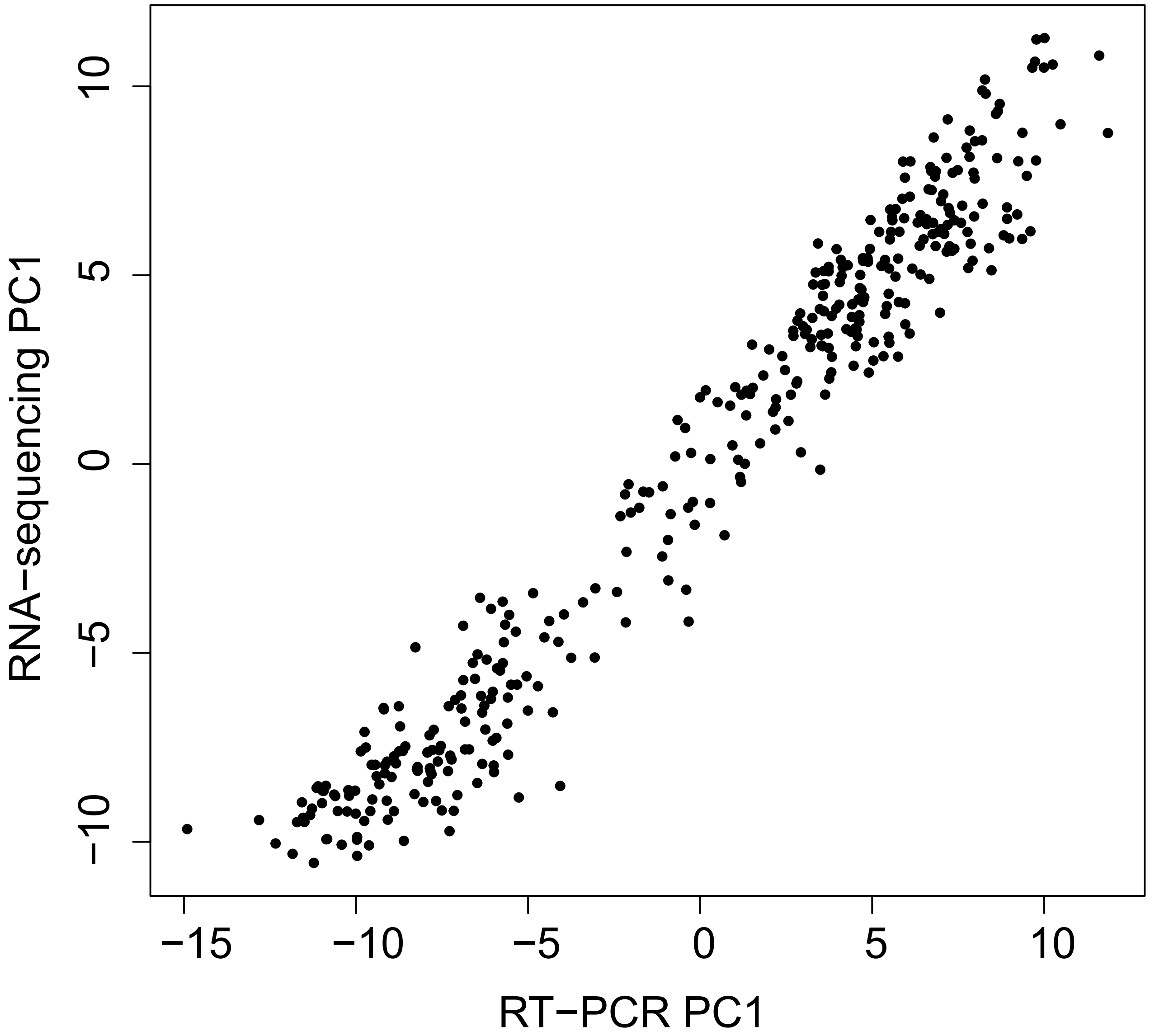


**Figure S27: Correlation of the first principal component of the IFN status gene set between real-time PCR and RNA-seq.** We calculated the first principal component for the IFN status 11 gene-set from real-time PCR and RNA-seq expression. The first principal components are highly correlated ($r_s$=0.96).

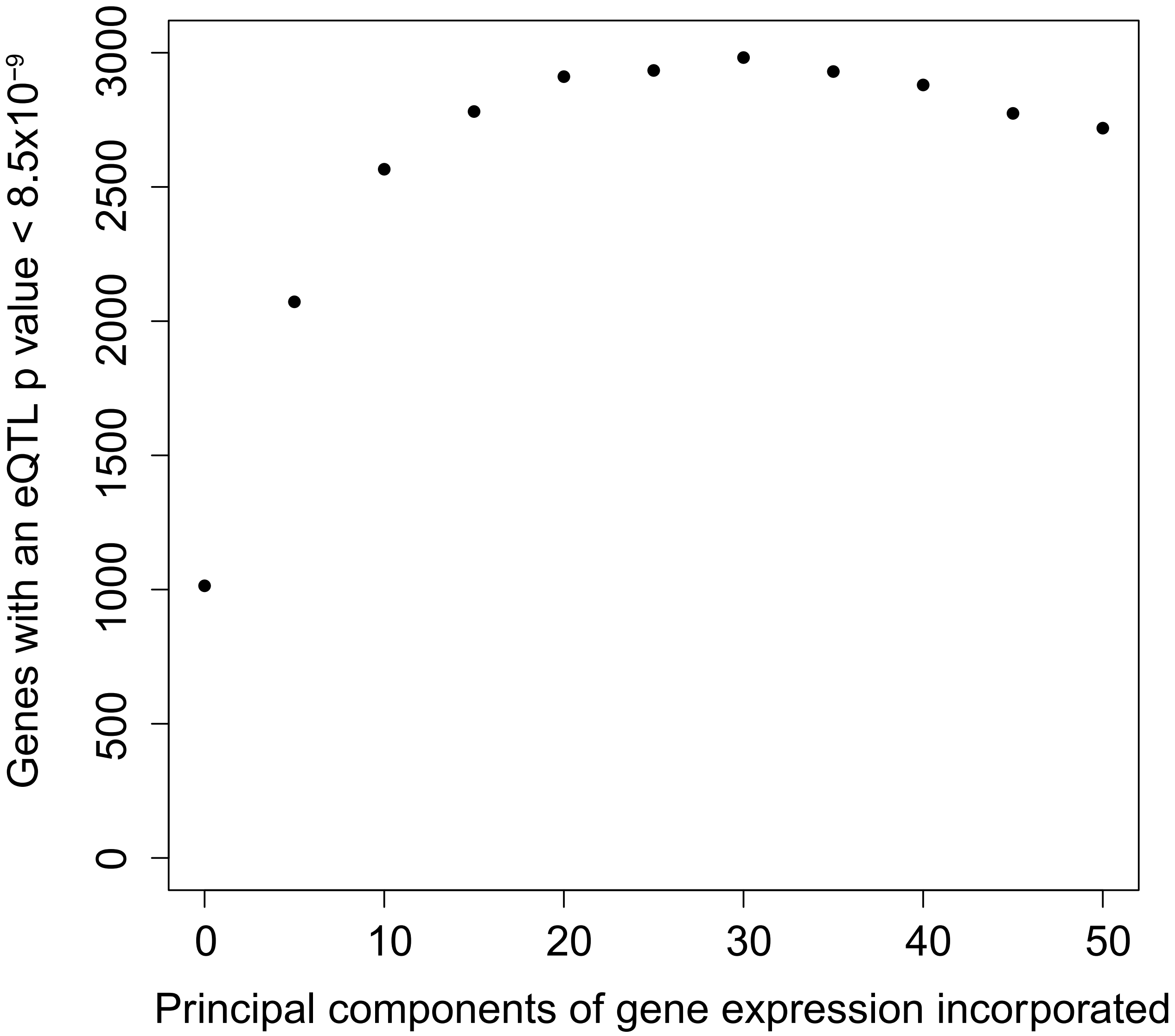


**Figure S28: eQTL genes identified with incremental increase in gene expression principal components incorporated.** Using the first available time point for each individual (n=157) we identified the number of significant eQTL genes after incrementally increasing the number of gene expression principal components accounted for in the linear model. 25 principal components of gene expression maximise the number of eQTL genes identified while minimising the number of covariates included.

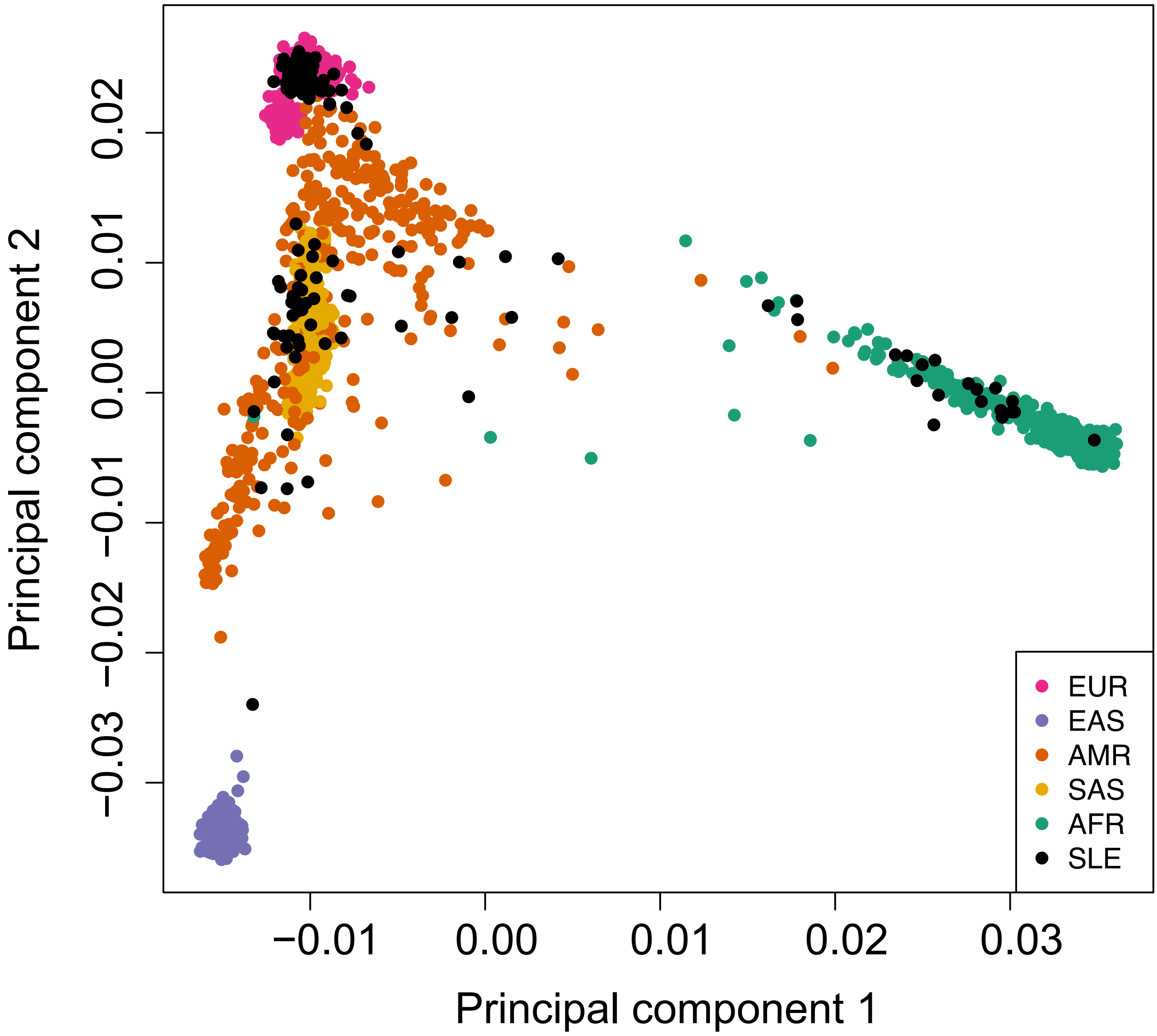


**Figure S29: Principal components of genotyping.** The first two principal components of genotyping for the SLE patients are plotted with the 1000 genomes super populations.

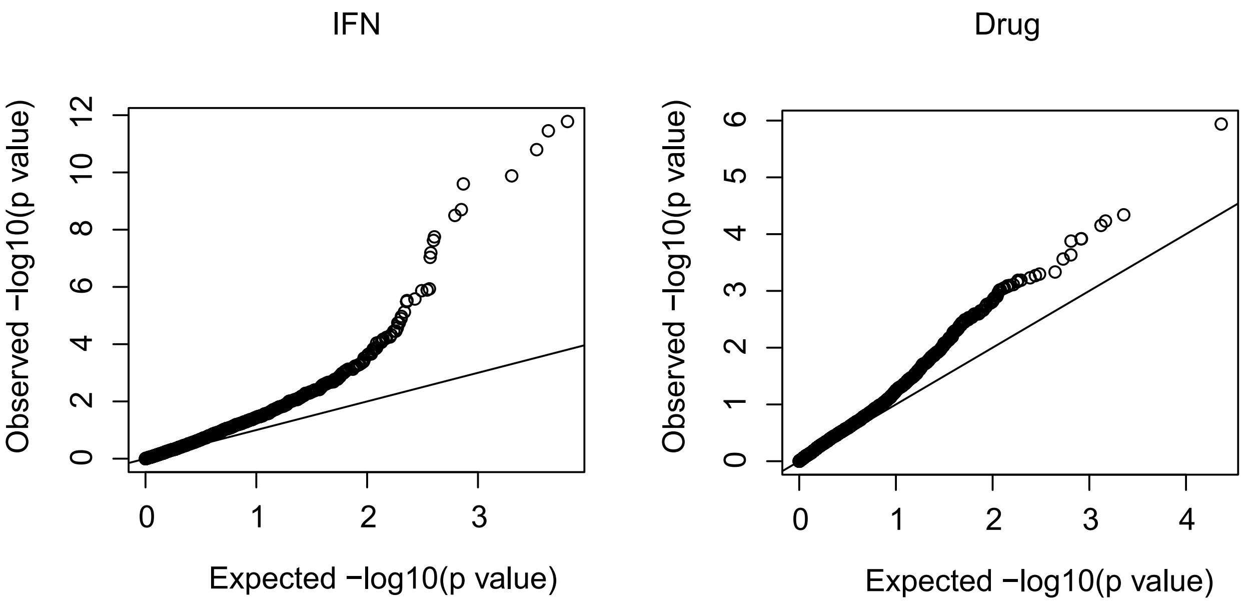


**Figure S30: Observed vs expected p values for each interaction analysis.** The expected p values were calculated from the uniform distribution.

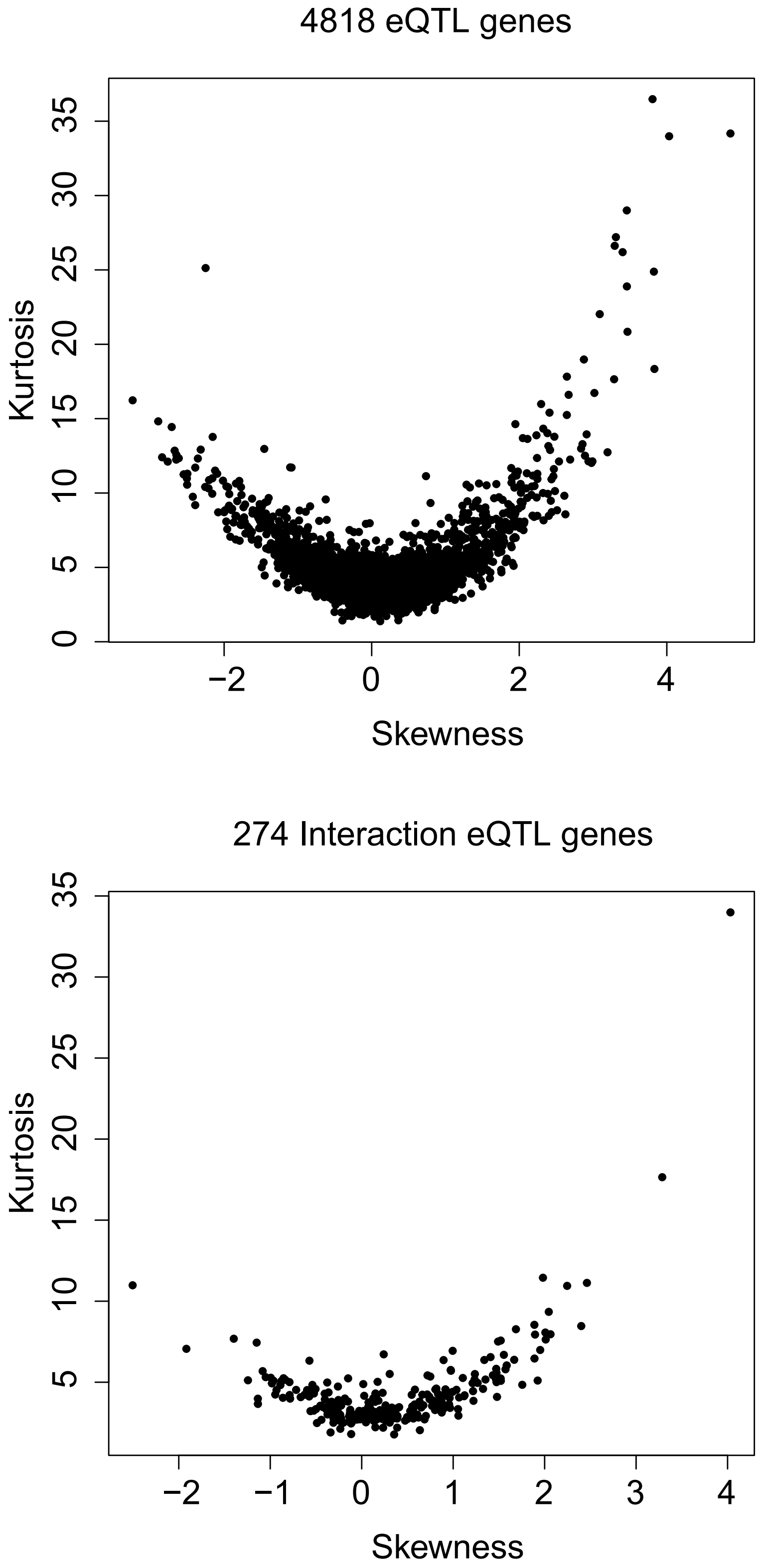


**Figure S31: Normality metrics of gene expression.** Normality metrics for the genes with a significant main eQTL effect ($p<8.5x10^{-9}$) and genes with a significant eQTL interaction effect (FDR<0.2).

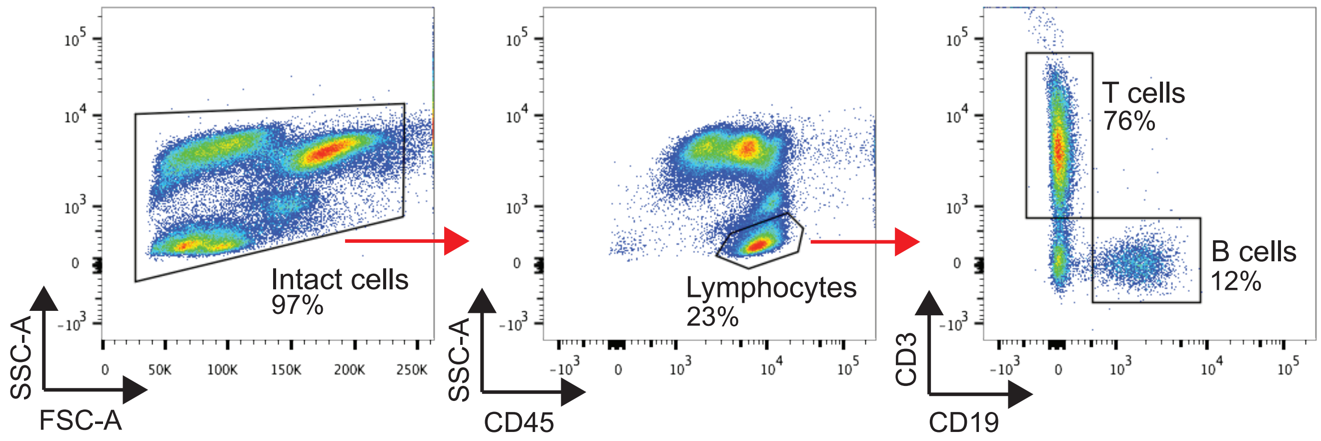


**Figure S32: Flow cytometry gating strategy.** Gating strategy for determining T and B cells as a percentage of lymphocytes.

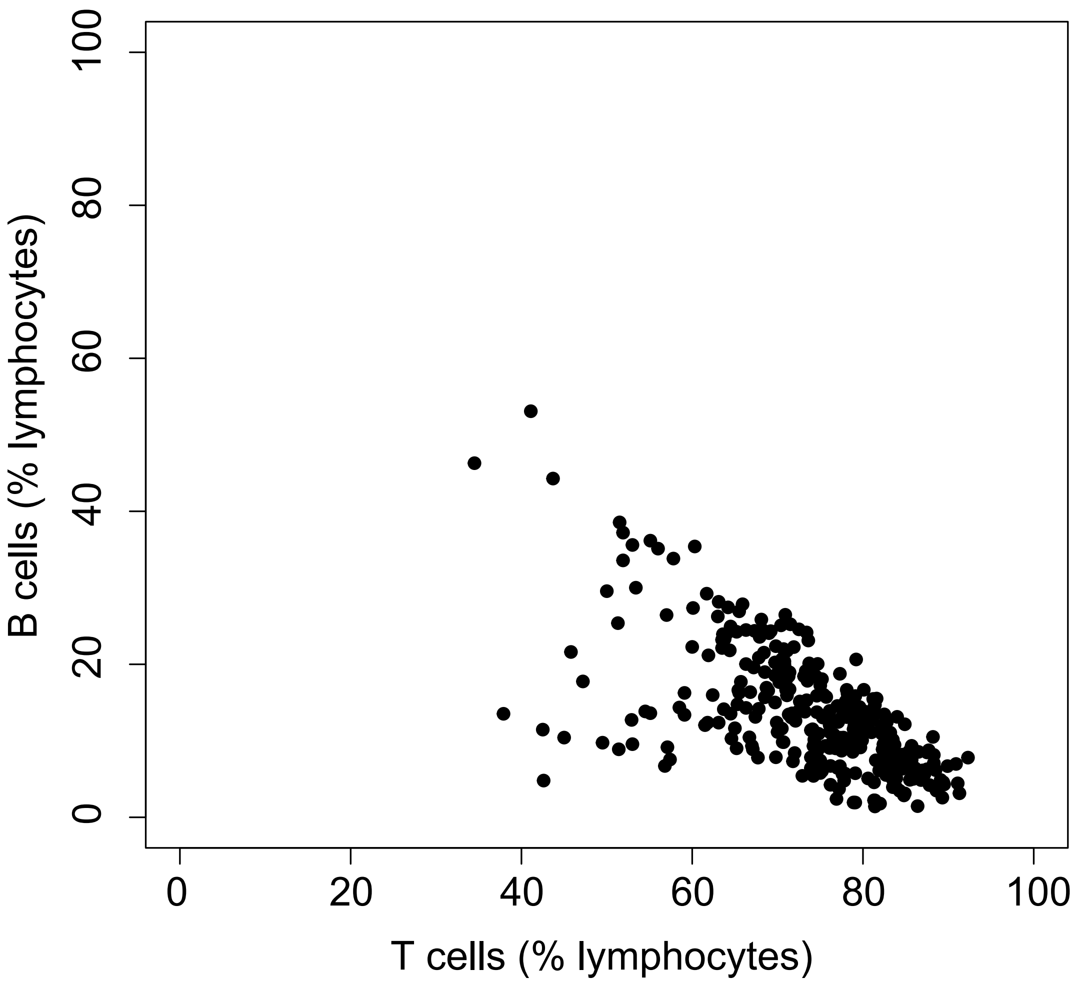


**Figure S33: Cell count correlation.** Correlation between T cell and B cell counts as a percentage of lymphocytes ($r_s$=-0.65).
