# Supplementary material for: Discovering in vivo cytokine-eQTL interactions from a lupus clinical trial
Source: Genome Biol. 2018 Oct 19;19:168. doi: 10.1186/s13059-018-1560-8 (PMC6195724; doi:10.1186/s13059-018-1560-8)
Supplement: Supplementary file 4 — Supplementary Table S7. (PDF 148 kb) [file 13059_2018_1560_MOESM4_ESM.pdf]

LIST OF INDEPENDENT ETHICS COMMITTEE (IEC) OR INSTITUTIONAL REVIEW BOARD (IRB)

ARGENTINA

Study Site Number	Independent Ethics Committee or Institutional Review Board Address(es)
1115	Comité de Ética en Investigación - CEMIC<br>Investigation Department<br>Galvan 4102, 1st Floor<br>C.A.B.A., C1431FWO<br>ARGENTINA
1116	Comite de Etica CAICI - CIAP<br>Comite de Etica CAICI - CIAP, Rodriguez 1198<br>Rosario, SANTA FE S2000PBJ<br>ARGENTINA
1119	Comite de Etica Institucional Framingham Centro Médico<br>Calle 9 N°431 e/40 y 41<br>La Plata, BUENOS AIRES B1902COS<br>ARGENTINA
1123	Comité Independiente de Etica para Ensayos en Farmacología Clínica<br>J.E. Uriburu 774 - Piso 1, Fundación de Estudios Farmacologicos y de Medicamentos (FEFyM)<br>Ciudad Autónoma de Buenos Aires, C1027AAP<br>ARGENTINA
1124	Comite Independiente de Etica para Ensayos en Farmacologia Clinica<br>J.E. Uriburu 774 - Piso 1<br>Ciudad Autónoma de Buenos Aires, C1027AAP<br>ARGENTINA

CHILE

Study Site Number	Independent Ethics Committee or Institutional Review Board Address(es)
1122	Comite de Etica Cientifico del Servicio de Salud Metropolitano Oriente<br>Av. Salvador 364<br>Providencia, SANTIAGO, RM 7600922<br>CHILE
1136	Comite Etico Cientifico del Servicio de Salud Metropolitano Oriente<br>Av. Salvador 364<br>Providencia, SANTIAGO, RM 7500922<br>CHILE
1138	Comite de Etico Cientifico del Servicio de Salud Metropolitano Oriente<br>Av. Salvador 364<br>Providencia, SANTIAGO, RM 7500922<br>CHILE

COLOMBIA

Study Site Number	Independent Ethics Committee or Institutional Review Board Address(es)
1117	Comite de Etica Riesgo de Fractura S.A<br>Carrera 13, No. 97-25<br>Bogota<br>COLOMBIA
1118	Comite de Etica en Investigación en el Area de la Salud de la Universidad del Norte<br>Km 5 Via a Puerto Colombia<br>Barranquilla, ATLANTICO 0<br>COLOMBIA
1121	Comite de Investigaciones y Etica en Investigaciones del Hospital Pablo Tobon Uribe<br>Calle 78 B No. 69-240<br>Medellin, ANTIOQUIA 0000<br>COLOMBIA
1131	Comite de Etica Medica e Investigacion Clinica Las Americas<br>Almacentro Cra. 43A N°34-155, Piso 6, Of. 609<br>Medellin, ANTIOQUIA<br>COLOMBIA
1132	Comite de Etica de la Investigacion Riesgo de Fractura S.A.<br>Carerra 13 #97-25<br>Bogota, Distrito Capital, CUNDINAMARCA<br>COLOMBIA
1133	Comite de ética en investigación Servimed E.U<br>Calle 51 No. 34-17, Cabecera I Etapa, Consultorio 208 C.C.<br>Bucaramanga, SANTANDER<br>COLOMBIA

GERMANY

Study Site Number	Independent Ethics Committee or Institutional Review Board Address(es)
1067	Ethik-Kommission der Medizinischen Fakultaet der Friedrich-Alexander-Universitaet Erlangen-Nuernberg<br>Krankenhausstrasse 12<br>Erlangen, 91054<br>GERMANY
1068	Ethik-Kommission der Medizinischen Fakultaet der Friedrich-Alexander-Universitaet Erlangen-Nuernberg<br>Krankenhausstrasse 12<br>Erlangen, 91054<br>GERMANY
1069	Ethik-Kommission der Medizinischen Fakultaet der Friedrich-Alexander-Universitaet Erlangen-Nuernberg<br>Krankenhausstrasse 12<br>Erlangen, 91054<br>GERMANY
1072	Ethik-Kommission der Medizinischen Fakultaet der Friedrich-Alexander-Universitaet Erlangen-Nuernberg<br>Krankenhausstrasse 12<br>Erlangen, 91054<br>GERMANY
1127	Ethik-Kommission der Medizinischen Fakultaet der Friedrich-Alexander-Universitaet Erlangen-Nuernberg<br>Krankenhausstrasse 12<br>Erlangen, 91054<br>GERMANY
1128	Ethik-Kommission der Medizinischen Fakultaet der Friedrich-Alexander-Universitaet Erlangen-Nuernberg<br>Krankenhausstrasse 12<br>Erlangen, 91054<br>GERMANY
1129	Ethik-Kommission der Medizinischen Fakultaet der Friedrich-Alexander-Universitaet Erlangen-Nuernberg<br>Krankenhausstrasse 12<br>Erlangen, 91054<br>GERMANY

Study Site Number	Independent Ethics Committee or Institutional Review Board Address(es)
1149	Ethik-Kommission der Medizinischen Fakultaet der Friedrich-Alexander-Universitaet Erlangen-Nuernberg<br>Krankenhausstrasse 12<br>Erlangen, 91054<br>GERMANY

HUNGARY

Study Site Number	Independent Ethics Committee or Institutional Review Board Address(es)
1112	Egeszsegugyi Tudomanyos Tanacs Klinikai Farmakologiai Etikai Bizottsaga<br>Arany J. u. 6-8.<br>Budapest, H-1051<br>HUNGARY
1125	Egeszsegugyi Tudomanyos Tanacs Klinikai Farmakologiai Etikai Bizottsaga<br>Arany J. u. 6-8.<br>Budapest, H-1051<br>HUNGARY
1126	Egeszsegugyi Tudomanyos Tanacs Klinikai Farmakologiai Etikai Bizottsaga<br>Arany J. u. 6-8.<br>Budapest, H-1051<br>HUNGARY
1184	Egészségügyi Tudományos Tanács<br>Klinikai Farmakológiai Etikai Bizottsága<br>Arany János u. 6-8<br>Budapest, 1051<br>HUNGARY

KOREA, REPUBLIC OF

Study Site Number	Independent Ethics Committee or Institutional Review Board Address(es)
1090	Yeouido St. Marys Hospital<br>62 Yeouido-dongInstitutional Review Board of the Catholic University of Korea, Yeongdeungpo-gu, Office #134<br>Seoul, 150-713<br>KOREA, REPUBLIC OF
1170	Gachon University Gil Hospital IRB<br>Namdong-guGachon University Gil Hospital, 1198, Guwol-dong<br>Incheon, 405-760<br>KOREA, REPUBLIC OF
1171	Dong-A University Medical Center Genetic Institutional Review Board<br>1, Dongdaesin-dong 3-ga<br>Seo-gu, BUSAN 602-715<br>KOREA, REPUBLIC OF
	Dong-A University Medical Center Institutional Review Board<br>Seo-gu, 1, Dongdaesin-dong 3-ga<br>Busan, 602-715<br>KOREA, REPUBLIC OF

MOLDOVA, REPUBLIC OF

Study Site Number	Independent Ethics Committee or Institutional Review Board Address(es)
1148	National Ethics Committee<br>27 Nicolae Testemiteanu St.<br>Chisinau, MD-2025<br>MOLDOVA, REPUBLIC OF

PERU

Study Site Number	Independent Ethics Committee or Institutional Review Board Address(es)
1108	Comite Institucional de Etica en Investigacion de la Universidad de<br>Av. Alameda del Corregidor 1531 Urb. Los Sirius, San Martin de Porres-Clínica CADAMUJER<br>La Molina-Lima, L-12<br>PERU
1109	Comite Institucional de Etica en Investigacion de la Universidad de<br>Av. Alameda del Corregidor 1531 Urb. Los Sirius, San Martin de Porres-Clínica CADAMUJER<br>La Molina-Lima, L-12<br>PERU
1110	Comite de Etica en Investigacion- Clinica Anglo Americana<br>Alfredo Salazar 350<br>San Isidro, LIMA Lima 27<br>PERU
	Comite Institucional de Etica en Investigacion de la Universidad de<br>Av. Alameda del Corregidor 1531 Urb. Los Sirius, San Martin de Porres-Clínica CADAMUJER<br>La Molina-Lima, L-12<br>PERU
1111	Comite Institucional de Etica de la Universidad Peruana Cayetano Heredia<br>Av. Honorio Delgado 430., Urb. Ingenieria. San Martin de Porres.<br>Lima, LIMA L31<br>PERU

POLAND

Study Site Number	Independent Ethics Committee or Institutional Review Board Address(es)
1063	Komisja Bioetyczna przy Okregowej Izbie Lekarskiej Wielkopolskiej Izby Lekarskiej<br>UL. Nowowiejskiego 51<br>Poznan, 61-734<br>POLAND
1064	Komisja Bioetyczna przy Okregowej Radzie Lekarskiej Wielkopolskiej Izby Lekarskiej<br>UL. Nowowiejskiego 51<br>Poznan, 61-734<br>POLAND
1066	Komisja Bioetyczna przy Okregowej Radzie Lekarskiej Wielkopolskiej Izby Lekarskiej<br>UL. Nowowiejskiego 51<br>Poznan, 61-734<br>POLAND
1141	Komisja Bioetyczna przy Okregowej Radzie Lekarskiej Wielkopolskiej Izby Lekarskiej<br>UL. Nowowiejskiego 51<br>Poznan, 61-734<br>POLAND

PUERTO RICO

Study Site Number	Independent Ethics Committee or Institutional Review Board Address(es)
1037	WESTERN IRB<br>WESTERN IRB<br>3535 7TH AVE SW<br>OLYMPIA, WA 98502<br>UNITED STATES<br><br>University of Puerto Rico<br>Medical Sciences Campus, Alan Preston Ph.D, PO Box 365067<br>San Juan, 00936-5067<br>PUERTO RICO

ROMANIA

Study Site Number	Independent Ethics Committee or Institutional Review Board Address(es)
1085	Comisia Naţională de Bioetică a Medicamentului şi a Dispozitivelor Medicale Ministerul Sanatatii<br>Sos. Stefan cel Mare nr.19-21, sector 2<br>Bucuresti, 020125<br>ROMANIA
1086	MINISTRY OF HEALTH<br>NATIONAL ETHICS COMMITTEE FOR THE CLINICAL STUDY OF MEDICINES<br>48, Av. Sanatescu Street, District 1<br>Bucharest, 011278<br>ROMANIA
1087	Comisia Naţională de Bioetică a Medicamentului şi a Dispozitivelor Medicale<br>Sos. Stefan cel Mare street, nr.19-21, sector 2<br>Bucuresti, 020125<br>ROMANIA
1183	Comisia Naţională de Bioetică a Medicamentului şi a Dispozitivelor Medicale<br>Sos. Stefan cel Mare nr.19-21<br>Bucuresti, SECTOR 2 020125<br>ROMANIA

TAIWAN

Study Site Number	Independent Ethics Committee or Institutional Review Board Address(es)
1081	National Taiwan University Hospital Research Ethics Committee<br>7, Chung-Shan South Road<br>Taipei, 100<br>TAIWAN
1082	China Medical University Hospital<br>No.2, Yude Rd., The Institutional Review Board<br>Taichung, 40447<br>TAIWAN
1083	Buddhist Tzu Chi General Hospital<br>Research Ethics Committee<br>707, Sec. 3, Chung-Yang Rd, Hualien City<br>Hualien, 97002<br>TAIWAN

UNITED STATES

Study Site Number	Independent Ethics Committee or Institutional Review Board Address(es)
1002	Copernicus Group Independent Review Board<br>One Triangle Dr, PO Box 110605, Ste 100<br>Research Triangle Park, NC 27709<br>UNITED STATES
1004	Copernicus Group Independent Review Board<br>One Triangle Dr, PO Box 110605, Ste 100<br>Research Triangle Park, NC 27709<br>UNITED STATES
1005	Cedars-Sinai Medical Center<br>383 Wilshire BoulevardInstitutional Review Board, Ste 742<br>Los Angeles, CA 90211<br>UNITED STATES
1008	Copernicus Group Independent Review Board<br>One Triangle Dr, PO Box 110605, Ste 100<br>Research Triangle Park, NC 27709<br>UNITED STATES
1009	Copernicus Group Independent Review Board<br>One Triangle Dr, PO Box 110605, Ste 100<br>Research Triangle Park, NC 27709<br>UNITED STATES
1010	University of Southern California Health Science Campus Institutional Review Board<br>LAC/USC Medical Center<br>1200 N State St, General Hospital Ste 4700<br>Los Angeles, CA 90033<br>UNITED STATES
1011	UCLA of the Human Research Protection Program<br>11000 Kinross Ave, Ste 211<br>Los Angeles, CA 90095-1694<br>UNITED STATES

Study Site Number	Independent Ethics Committee or Institutional Review Board Address(es)
1015	University of Texas Southwestern Medical Center<br>5323 Harry Hines BoulevardInstitutional Review Board, C1.206<br>Dallas, TX 75390-8843<br>UNITED STATES
1017	Biomedical Research Alliance of New York (BRANY) Institutional Review Board<br>1981 Marcus Ave, Ste 210<br>Lake Success, NY 11042<br>UNITED STATES
1018	Copernicus Group Independent Review Board<br>One Triangle Dr, PO Box 110605, Ste 100<br>Research Triangle Park, NC 27709<br>UNITED STATES
1019	Copernicus Group Independent Review Board<br>One Triangle Dr, PO Box 110605, Ste 100<br>Research Triangle Park, NC 27709<br>UNITED STATES
1020	Oklahoma Medical Research Foundation<br>825 Ne 13th St<br>Oklahoma City, OK 73104<br>UNITED STATES
1021	New York University School of Medicine Institutional Review Board<br>One Park Ave, Sixth Fl<br>New York, NY 10016<br>UNITED STATES
1022	Copernicus Group Independent Review Board<br>One Triangle Dr, PO Box 110605, Ste 100<br>Research Triangle Park, NC 27709<br>UNITED STATES

Study Site Number	Independent Ethics Committee or Institutional Review Board Address(es)
1023	The University of Chicago Institutional Review Board<br>5751 S Woodlawn Ave, McGiffert Hall, 2nd Fl<br>Chicago, IL 60637<br>UNITED STATES
1024	The University of North Carolina at Chapel Hill<br>School of Medicine Bldg 52Office of Human Research Ethics, CB 7097<br>Chapel Hill, NC 27599-7097<br>UNITED STATES
1025	MedStar Health Research Institute<br>6525 Belcrest Rd, Ste 700<br>Hyattsville, MD 20782<br>UNITED STATES
1026	Copernicus Group Independent Review Board<br>One Triangle Dr, PO Box 110605, Ste 100<br>Research Triangle Park, NC 27709<br>UNITED STATES
1027	Copernicus Group Independent Review Board<br>One Triangle Dr, PO Box 110605, Ste 100<br>Research Triangle Park, NC 27709<br>UNITED STATES
1028	Copernicus Group Independent Review Board<br>One Triangle Dr, PO Box 110605, Ste 100<br>Research Triangle Park, NC 27709<br>UNITED STATES
1030	Copernicus Group Independent Review Board<br>One Triangle Dr, PO Box 110605, Ste 100<br>Research Triangle Park, NC 27709<br>UNITED STATES

Study Site Number	Independent Ethics Committee or Institutional Review Board Address(es)
1033	University of Michigan Medical School Institutional Review Board - IRBMED<br>2800 Plymouth Rd, Bldg 520, Ste 3214<br>Ann Arbor, MI 48109-2800<br>UNITED STATES
1034	Johns Hopkins Medicine Institutional Review Board<br>1620 McElderry Streeet, Reed Hall - B130<br>Baltimore, MD 21205<br>UNITED STATES
1036	Western Institutional Review Board<br>1019 39th Ave S E<br>Puyallup, WA 98374<br>UNITED STATES
1040	Copernicus Group<br>PO Box 110605Institutional Review Board, One Triangle Dr, Ste 100<br>Research Triangle Park, NC 27709<br>UNITED STATES
1041	Copernicus Group Independent Review Board<br>P.O 110605, Copernicus Group Independent Review Board<br>Research Triangle Park, NC 27709<br>UNITED STATES
	Copernicus Group Independent Review Board<br>P.O. Box 110605, One Triangle Dr, Ste 100<br>Research Triangle Park, NC 27709<br>UNITED STATES
1043	Emory University Institutional Review Board<br>1599 Clifton Rd, 5th Fl, E<br>Atlanta, GA 30322<br>UNITED STATES

Study Site Number	Independent Ethics Committee or Institutional Review Board Address(es)
1044	Cleveland Clinic Institutional Review Board<br>Cleveland Clinic Foundation<br>9500 Euclid Ave, OS-1<br>Cleveland, OH 44195<br>UNITED STATES
1046	Copernicus Group<br>P.O. Box 110605Institutional Review Board, One Triangle Dr, Ste 100<br>Research Triangle Park, NC 27709<br>UNITED STATES
1047	Tufts MedicaI Center/Tufts University Institutional Review Board<br>800 Washington St, Box 817<br>Boston, MA 02111<br>UNITED STATES
1050	Copernicus Group<br>P.O. Box 110605Institutional Review Board, One Triangle Dr, Ste 100<br>Research Triangle Park, NC 27709<br>UNITED STATES
1052	Copernicus Group Independent Review Board<br>Ste 100, One Triangle Dr, PO Box 110605<br>Durham, NC 27709<br>UNITED STATES
1055	Copernicus Group Independent Review Board<br>P.O. Box 110605, One Triangle Dr, Ste 100<br>Research Triangle Park, NC 27709<br>UNITED STATES
	Copernicus Group Independent Review Board<br>P.O 110605, Copernicus Group Independent Review Board<br>Research Triangle Park, NC 27709<br>UNITED STATES

Study Site Number	Independent Ethics Committee or Institutional Review Board Address(es)
1056	Copernicus Group Independent Review Board<br>P.O. Box 110605, One Triangle Dr, Ste 100<br>Research Triangle Park, NC 27709<br>UNITED STATES
	Copernicus Group Independent Review Board<br>P.O 110605, Copernicus Group Independent Review Board<br>Research Triangle Park, NC 27709<br>UNITED STATES
1062	Copernicus Group Independent Review Board<br>P.O 110605, Copernicus Group Independent Review Board<br>Research Triangle Park, NC 27709<br>UNITED STATES
	Copernicus Group Independent Review Board<br>P.O. Box 110605, One Triangle Dr, Ste 100<br>Research Triangle Park, NC 27709<br>UNITED STATES
1143	Copernicus Group Independent Review Board<br>One Triangle Dr, PO Box 110605, Ste 100<br>Research Triangle Park, NC 27709<br>UNITED STATES
1144	Western Institutional Review Board<br>3535 Seventh AVE SW<br>OLYMPIA, WA 98502<br>UNITED STATES
1152	Copernicus Group Independent Review Board<br>P.O 110605, Copernicus Group Independent Review Board<br>Research Triangle Park, NC 27709<br>UNITED STATES

Study Site Number	Independent Ethics Committee or Institutional Review Board Address(es)
	Copernicus Group Independent Review Board<br>P.O. Box 110605, One Triangle Dr, Ste 100<br>Research Triangle Park, NC 27709<br>UNITED STATES
1155	Henry Ford Health System<br>2799 W Grand BoulevardInstitutional Review Board, CFP Bsmt, Rm 046 Henry Ford Hospital, Research Administration<br>Detroit, MI 48202<br>UNITED STATES
1156	Copernicus Group Independent Review Board<br>P.O 110605, Copernicus Group Independent Review Board<br>Research Triangle Park, NC 27709<br>UNITED STATES
	Copernicus Group Independent Review Board<br>P.O. Box 110605, One Triangle Dr, Ste 100<br>Research Triangle Park, NC 27709<br>UNITED STATES
1159	Copernicus Group<br>P.O. Box 110605Institutional Review Board, One Triangle Dr, Ste 100<br>Research Triangle Park, NC 27709<br>UNITED STATES
1160	Copernicus Group Independent Review Board<br>P.O. Box 110605, One Triangle Dr, Ste 100<br>Research Triangle Park, NC 27709<br>UNITED STATES
1161	Copernicus Group Independent Review Board<br>P.O. Box 110605, One Triangle Dr, Ste 100<br>Research Triangle Park, NC 27709<br>UNITED STATES

Study Site Number	Independent Ethics Committee or Institutional Review Board Address(es)
	Copernicus Group Independent Review Board<br>P.O 110605, Copernicus Group Independent Review Board<br>Research Triangle Park, NC 27709<br>UNITED STATES
1163	Copernicus Group<br>P.O. Box 110605Institutional Review Board, One Triangle Dr, Ste 100<br>Research Triangle Park, NC 27709<br>UNITED STATES
1165	Copernicus Group Independent Review Board<br>P.O 110605, Copernicus Group Independent Review Board<br>Research Triangle Park, NC 27709<br>UNITED STATES
	Copernicus Group Independent Review Board<br>P.O. Box 110605, One Triangle Dr, Ste 100<br>Research Triangle Park, NC 27709<br>UNITED STATES
1169	Copernicus Group Independent Review Board<br>One Triangle Dr, Ste 100<br>Research Triangle Park, NC 27709<br>UNITED STATES
1172	Indiana University Institutional Review Board c/o Research Compliance Administration<br>980 Indiana Ave, Lockefield, 3rd Fl<br>Indianapolis, IN 46202<br>UNITED STATES
1173	Copernicus Group Independent Review Board<br>P.O. Box 110605, One Triangle Dr, Ste 100<br>Research Triangle Park, NC 27709<br>UNITED STATES

Study Site Number	Independent Ethics Committee or Institutional Review Board Address(es)
1174	Copernicus Group Independent Review Board<br>P.O. Box 110605, One Triangle Dr, Ste 100<br>Research Triangle Park, NC 27709<br>UNITED STATES
	Copernicus Group Independent Review Board<br>P.O 110605, Copernicus Group Independent Review Board<br>Research Triangle Park, NC 27709<br>UNITED STATES
1179	Copernicus Group Independent Review Board<br>P.O. Box 110605, One Triangle Dr, Ste 100<br>Research Triangle Park, NC 27709<br>UNITED STATES
	Copernicus Group Independent Review Board<br>P.O 110605, Copernicus Group Independent Review Board<br>Research Triangle Park, NC 27709<br>UNITED STATES
1189	Copernicus Group Independent Review Board<br>P.O 110605, Copernicus Group Independent Review Board<br>Research Triangle Park, NC 27709<br>UNITED STATES
	Copernicus Group Independent Review Board<br>P.O. Box 110605, One Triangle Dr, Ste 100<br>Research Triangle Park, NC 27709<br>UNITED STATES
1191	Copernicus Group Independent Review Board<br>P.O. Box 110605, One Triangle Dr, Ste 100<br>Research Triangle Park, NC 27709<br>UNITED STATES

Study Site Number	Independent Ethics Committee or Institutional Review Board Address(es)
	Copernicus Group Independent Review Board<br>P.O 110605, Copernicus Group Independent Review Board<br>Research Triangle Park, NC 27709<br>UNITED STATES
