# Supplementary material for: Discovering in vivo cytokine-eQTL interactions from a lupus clinical trial
Source: Genome Biol. 2018 Oct 19;19:168. doi: 10.1186/s13059-018-1560-8 (PMC6195724; doi:10.1186/s13059-018-1560-8)
Supplement: Supplementary file 3 — Review history. (DOCX 841 kb) [file 13059_2018_1560_MOESM3_ESM.docx]

**First round of review**

**Reviewer 1**

**Are the methods appropriate to the aims of the study, are they well described, and are necessary controls included?**
Yes

**Are the conclusions adequately supported by the data shown?**

Mostly

**Are sufficient details provided to allow replication and comparison with related analyses that may have been performed?**

Yes

**Does the work represent a significant advance over previously published studies? Please explain.**

The study of eqtls in patient samples and the extent of treatment effects is a topic of interest that has not been explored in depth. this dataset is an important contribution to that literature.

**Is the paper of broad interest to others in the field, or of outstanding interest to a broad audience of biologists?**

Yes: the study of eqtls in patient samples and the extent of treatment effects is a topic of interest that has not been explored in depth. this dataset is an important contribution to that literature.

**Comments to authors**

This manuscript describes an eQTL study in whole blood in SLE patients from an anti-IL-6 clinical trial involving 157 subjects. A mixed model is used appropriately to account for repeated measures data. cis eQTLs are identified, then assessed for interaction between genotype and either IFN status or anti-IL-6 exposure. Identifying eQTLs in patient populations is important, and detecting interactions with non-genetic factors is also of interest, so I think this is a useful and timely paper. It is well written and methods are clearly set out. The concerns I have relate to whether these interactions are real or artifacts of the analytical strategy, or whether the reflect the analysis of a heterogeneous mix of cells.

major comments:
- the design is to adjust expression data for PCs, which will surely adjust away many of the components of the IFN signature. The cis-eQTLs are discovered and each of these are examined for interaction with IFN status. I wonder whether adjusting the expression data in this way in fact means that an eQTL that was truly affected by IFN status but in a strictly additive manner (expr = b1 * genotype + b2 * ifn + error), would appear to have an interaction effect under this strategy. I realise the analysis was repeated with a single PC removed, and fig S9 suggests the exclusion of the PC results in more extreme p values, but I would like to see a more thorough confirmation that these are genuine interactions
- did each interaction show a marginal effect with IFN/anti-IL-6? Marginal effects of either on expression are not reported, which seems odd when searching for interactions, especially given the detailed attention given to marginal effects of SNPs. The effect of beta_drug was certainly fitted (eqn on p 24), why not report it?
- to what extent do these "interactions" relate to cell type specific effects? Does IFN status or anti-IL-6 administration correlate with differential cell counts in blood? Given that blood is such a heterogenous mix of cells each with its own set of eQTL effects, and that the proportion of each cell type varies between individuals, how can the authors state with such confidence that the interactions they see are truly due to IFN or anti-IL-6? If either of these factors correlated with variability in cell type proportions between samples, we might expect a similar set of results.
- I thank the authors for making their complete data available in an interactive form. However, it would be more useful to be able to download the data, and currently the link highlighted in "You can also download the Lupus_eQTL.zip file to your local PC, unzip it and start exploring by opening index.html in Chrome or Firefox even without WIFI. " 404s. Also, these data should be placed in a public repository so their continued access can be guaranteed. Finally, the availability of the raw data underlying the results should be carefully described, currently they are not mentioned.

minor:
- please mention that the study is of whole blood in the abstract
- p 12: I am of the view that the term "suggestive" in relation to interpreting p values close to 0.05 should be banned. The authors should simply state that the ratio is interesting, but not significant.
- p 23: please take more care to define the algebraic terms used. g_j, phi_i and gamma_m are not defined. Also, shouldn't it be kappa_i | j, not kappa_j | i (sample i nested within subject j)?

**Reviewer 2**

**Are the methods appropriate to the aims of the study, are they well described, and are necessary controls included?**

see comments below

**Are the conclusions adequately supported by the data shown?**

see comments below

**Are sufficient details provided to allow replication and comparison with related analyses that may have been performed?**

see comments below

**Does the work represent a significant advance over previously published studies? Please explain.**

see comments below

**Is the paper of broad interest to others in the field, or of outstanding interest to a broad audience of biologists?**

Yes: see comments below

**Comments to authors**

The manuscript by Davenport et al. addresses the important question of the interaction between environment (disease and drug) and eQTLs. Using the framework of a clinical trial for experimental design is beneficial, as individuals can be used as blocks, which makes it easier to establish baseline and to detect interaction. Mixed-effects models are suitable for such a repeated measures design. Incorporating PCs of gene expression and genotypes is helpful to control confounding. The searchable and sortable spreadsheet of interaction eQTLs available on GitHub, along with the visualization for each SNP-gene pair, is helpful. Data analysis needs clarification and some revision.

1. Filtering eQTLs with the linear model in line 536 on p 23 is rather unnecessary. This step makes it harder to identify eQTLs whose effect is dampened by other variables, unless the eQTL effect remains strong after dampening. 

2. It seems that the authors analyzed IFN status and drug exposure separately. This approach implies the assumption that drug exposure does not impact the IFN status. If this is a valid assumption, the authors need to justify it in the paper. Otherwise, it would be more appropriate to have a complete model that adds IFN status, interaction between IFN and eQTL (beta_x_IFN), drug exposure (beta_drug), interaction between drug and eQTL (beta_x_drug), and (if needed) interaction between IFN and drug exposure to the basic model in line 536.

This complete model allows for detection of eQTLs, interactions, and differential gene expression in one step, which is equivalently to testing whether beta_geno, beta_x_IFN, beta_x_drug, and beta_drug are zero, respectively. 

In line 302-303 on p 13, the authors state that 'only 1/72 drug-eQTL interaction genes also shows evidence of differential gene expression'. This result indicates that the coefficients for drug exposure (beta_drug; or equivalently, the main effect of drug exposure) in the linear model may be weak, or even insignificant. What does drug interaction mean when the main effect is almost nonexistent? If the main effect is indeed nonexistent, then the significant interaction estimated from the linear model might be an artifact; the interaction effect may come from other relevant predictors that are not included in the model. The complete model would be helpful for addressing this issue. If beta_drug and beta_x_drug are still significant after IFN status is accounted for, then drug interaction would be providing additional information ('independent information' as the authors suggested) unaccounted for by differential gene expression induced by drug exposure.

By analyzing all the genes with this complete model, one can then more easily account for multiple testing, either with the stringent Bonferroni correction to control the overall type I error, or with the less stringent q value method to control for FDR. 

3. Table S4 looks at correlation between the gene expression PCs in the linear model and potential confounders, such as age, sex and site. None of the PCs have a strong correlation with these confounders, meaning that these confounders contain information not captured by the PCs. Why are they not included in the linear model?

Minor comments:
1. If the formatting requirements allow, it would be helpful to refer in the main text to specific sections in Methods, instead of simply 'Methods'.

2. The authors may want to streamline the presentation of the number of patients and the number of samples in the Methods. 
Study design: 183 patients, 183x3=549 samples;
RNA-sequencing: 180 patients, 468-4=464 samples;
Genotyping: 159 patients;
Interferon status: 157 patients, 376 samples;
Free IL-6 protein levels: 145 patients, 311 samples;
Statistical analysis: 157 patients, 379 samples (numbers used in main text).

3. Line 242-249 on p 10, analysis of TF binding motifs. What do foreground and background mean? Can the authors provide an example to illustrate these concepts?

4. Line 564 on p 24 says that 'we determined the significance of the interaction term with a likelihood ratio test'. Can the authors elaborate on this? Table S1 summarizes the statistics for the eQTLs, but the statistics for interaction seem to be t statistics, which are used for an exact test in small samples, and not for a likelihood ratio test, which is typically an approximation at a large sample size. Which test was used then?

5. Line 512-513 on p 22, 'we first applied a linear model for the first available time point (week 0 samples for n=152, week 12 samples for n=5) to identify each eQTL...' Why is week 12 the first time point for 5 patients? Fig 1A suggests that all 157 patients have data from week 0.

6. Line 253 on p 11, fig 3C should be 2C.

7. What's the rationale behind the definition of the drug exposure score?

8. Lupus_eQTL.zip is unavailable on the GitHub page: https://baohongz.github.io/Lupus_eQTL/ (Mar 26, 2018).

9. The authors provide gene expression data in Table S6, where each row is a gene and each column is a sample. However, there is no information on which samples are from which patients with what treatment and from which time point. Perhaps the zip file on the GitHub page has this information?

**Authors’ response to reviewers**

*This manuscript describes an eQTL study in whole blood in SLE patients from an anti-IL-6 clinical trial involving 157 subjects. A mixed model is used appropriately to account for repeated measures data. cis eQTLs are identified, then assessed for interaction between genotype and either IFN status or anti-IL-6 exposure. Identifying eQTLs in patient populations is important, and detecting interactions with non-genetic factors is also of interest, so I think this is a useful and timely paper. It is well written and methods are clearly set out.*

We thank the reviewer for these encouraging comments. *The concerns I have relate to whether these interactions are real or artifacts of the analytical strategy, or whether the reflect the analysis of a heterogeneous mix of cells.*

major comments:

Comment 1

*- the design is to adjust expression data for PCs, which will surely adjust away many of the components of the IFN signature. The cis-eQTLs are discovered and each of these are examined for interaction with IFN status. I wonder whether adjusting the expression data in this way in fact means that an eQTL that was truly affected by IFN status but in a strictly additive manner (expr = b1 * genotype + b2 * ifn + error), would appear to have an interaction effect under this strategy. I realise the analysis was repeated with a single PC removed, and fig S9 suggests the exclusion of the PC results in more extreme p values, but I would like to see a more thorough confirmation that these are genuine interactions*

Response 1

We thank the reviewer for raising this important point. Our baseline model for comparison does indeed include an additive term and therefore we test the interaction term in the presence of that additive term. We wanted to clarify this in this text to avoid confusion.

To more extensively assess whether adjusting for gene expression PCs might inflate interaction terms, we have conducted an additional analysis to identify eQTL interactions with IFN status without correcting for any gene expression PCs. The IFN eQTL interaction betas were highly correlated (rs=0.88) between analyses where PCs were excluded and included. Furthermore, including principal components on average nominally reduces the magnitude of the interaction terms (-7%) rather than inflates them. We have added Figure S11 and the following text to the results.

Results: To further explore this, we also repeated the IFN interaction analysis without correcting for any expression principal components. We find the betas for the interaction term are highly correlated (rs=0.88, **Figure S11**).

Comment 2

*- did each interaction show a marginal effect with IFN/anti-IL-6? Marginal effects of either on expression are not reported, which seems odd when searching for interactions, especially given the detailed attention given to marginal effects of SNPs. The effect of beta_drug was certainly fitted (eqn on p 24), why not report it?*

Response 2

We thank the reviewer for their suggestion and have included a more extensive summary of differential expression in the manuscript. As further detailed in Response 2 to Reviewer 2, we have reported the differential expression for both IFN and anti-IL-6 without PC correction as the presence of PC correction has the effect of obscuring differential expression values. We have summarized the differential expression results in Tables S6 and S7 and have included the following additions to the manuscript text.

Results: For IFN status, we identified 1,850 differentially expressed genes (FDR<0.05 and fold

change>1.2, **Figure S16, Table S6**). Only 42/210 IFN-eQTL interaction genes also show

evidence of differential expression. For differential expression following anti-IL-6 treatment, we

identified 394 genes (FDR<0.05 and fold change>1.2, **Figure S16, Table S7**). Intriguingly, none

of the 72 drug-eQTL interaction genes show evidence of differential gene expression.

Comment 3

*- to what extent do these "interactions" relate to cell type specific effects? Does IFN status or anti-IL-6 administration correlate with differential cell counts in blood? Given that blood is such a heterogenous mix of cells each with its own set of eQTL effects, and that the proportion of each cell type varies between individuals, how can the authors state with such confidence that*

*the interactions they see are truly due to IFN or anti-IL-6? If either of these factors correlated with variability in cell type proportions between samples, we might expect a similar set of results.*

Response 3

We agree with the reviewer that the cell type specific effects of interactions is an important pointhowever, we lack the complete blood counts that would allow us to answer this question most thoroughly. We did have FACS data for B and T cells. We have updated the manuscript with these additional data and show that IFN and anti-IL6 are not correlated with either B or T cell counts.

Discussion: We note that as we have conducted our study on whole blood, some of our observed interactions could be driven by variability in cell type proportions between individuals. While we lack the complete blood counts to explore this thoroughly, we determined B and T cell abundance from FACS data (**Methods: Cell counts**). We find that IFN and exposure to anti-IL-6 are not correlated with either B or T cell abundance (**Figure S20**). While further studies will be required to determine if certain interactions are being driven by specific cell types, it is unlikely that B or T cell proportions are driving the interactions we observe here.

Methods: We collected 4ml whole blood in sodium heparin vacutainers for cytometry analysis at weeks 0, 12 and 24. Samples were subjected to flow cytometry for T cell and B cell immunophenotyping (**Figure S30**). We counted T (CD3+) and B (CD19+) cells as a percentage of lymphocytes (CD45+, SSC-small) because of the abnormal distribution of lymphocytes observed in SLE[61]. These counts are therefore inversely correlated (rs=-0.65, **Figure S31**). FACS data were available for 320 samples from 152 subjects.

Comment 4

*- I thank the authors for making their complete data available in an interactive form. However, it would be more useful to be able to download the data, and currently the link highlighted in "You can also download the Lupus_eQTL.zip file to your local PC, unzip it and start exploring by opening index.html in Chrome or Firefox even without WIFI. " 404s. Also, these data should be placed in a public repository so their continued access can be guaranteed. Finally, the availability of the raw data underlying the results should be carefully described, currently they are not mentioned.*

Response 4

We thank the reviewer for noticing this error. We have corrected the link so the Lupus_eQTL.zip file can now be downloaded. Additionally, as described in Response 12 to Reviewer 2, we have updated the expression data in Table S8 to indicate which sample is from which individual, treatment and time point.

We agree with the reviewer that we should make the raw data available in public repositories.

Consequently, we are now in the process of making RNA-seq and genotyping data available in NCBI’s GEO and dbGaP respectively, and anticipate that by publication of this manuscript the data should be available to the general public.

Availability of Data: "We are in the process of making raw RNA-seq and genotyping data

available in NCBI’s GEO and dbGAP respectively."

minor:

Comment 5

*- please mention that the study is of whole blood in the abstract*

Response 5

We have added this to the abstract

Abstract: In a clinical trial for anti-IL-6 in patients with systemic lupus erythematosus we measured interferon (IFN) status, anti-IL-6 drug exposure and whole blood genome-wide gene expression at three time points (379 samples from 157 individuals).

Comment 6

*- p 12: I am of the view that the term "suggestive" in relation to interpreting p values close to 0.05 should be banned. The authors should simply state that the ratio is interesting, but not significant.*

Response 6

We have amended the text

Results: We permuted the magnifying and dampening genes and found this ratio for enrichment is interesting at the gene level but not significant (p=0.058) and therefore additional eQTL interactions will be necessary to confirm.

Comment 7

*- p 23: please take more care to define the algebraic terms used. g_j, phi_i and gamma_m are not defined. Also, shouldn't it be kappa_i | j, not kappa_j | i (sample i nested within subject j)?*

Response 7

We thank the reviewer for highlighting this omission. We have added to the text to define all the algebraic terms and have corrected kappa_i|j.

Methods: Where Ei,j is gene expression for the ith sample from the jth subject, θ is the intercept, βgeno is the effect (eQTL) of the genotype for individual j (gj), (κi|j) is the random effect for the ith sample from the jth subject, ϕl is the effect of principal component l of gene expression for sample i (pci,l) and γm is the effect of principal component m of genotyping for subject j (pcj,m).

**Reviewer #2:**

*The manuscript by Davenport et al. addresses the important question of the interaction between environment (disease and drug) and eQTLs. Using the framework of a clinical trial for experimental design is beneficial, as individuals can be used as blocks, which makes it easier to establish baseline and to detect interaction. Mixed-effects models are suitable for such a repeated measures design. Incorporating PCs of gene expression and genotypes is helpful to control confounding. The searchable and sortable spreadsheet of interaction eQTLs available on GitHub, along with the visualization for each SNP-gene pair, is helpful. Data analysis needs clarification and some revision.*

Comment 1

*Filtering eQTLs with the linear model in line 536 on p 23 is rather unnecessary. This step makes it harder to identify eQTLs whose effect is dampened by other variables, unless the eQTL effect remains strong after dampening.*

Response 1

We agree with the reviewer that we are indeed taking a conservative approach and that this might reduce the number of interactions we are detecting. However, given the challenge of identifying interactions, we wanted to ensure that we were confident in the eQTL main effect before testing that effect for an interaction. The *SLFN5* interaction with IFN (Figure 2B) shows we are still able to detect interactions even when the eQTL effect is very modest in one group.

We have added the following text to the discussion to address this point.

Discussion: We acknowledge that our approach for eQTL discovery using a stringent Bonferroni corrected p value threshold is conservative and could reduce our ability to detect eQTLs with a modest effect in one group and therefore reduce the number of interactions we observe. However, given the challenge of identifying interactions, we wanted to ensure that we were confident in the eQTL effect before testing that effect for an interaction. Furthermore, as demonstrated by the SLFN5 IFN-eQTL interaction, we still observe interaction examples where an eQTL effect is very modest in one group, in this case, samples designated as IFN low (Figure 2B).

Comment 2

*It seems that the authors analyzed IFN status and drug exposure separately. This approach implies the assumption that drug exposure does not impact the IFN status. If this is a valid assumption, the authors need to justify it in the paper. Otherwise, it would be more appropriate to have a complete model that adds IFN status, interaction between IFN and eQTL (beta_x_IFN), drug exposure (beta_drug), interaction between drug and eQTL (beta_x_drug), and (if needed) interaction between IFN and drug exposure to the basic model in line 536.*

*This complete model allows for detection of eQTLs, interactions, and differential gene*

*expression in one step, which is equivalently to testing whether beta_geno, beta_x_IFN, beta_x_drug, and beta_drug are zero, respectively.*

*In line 302-303 on p 13, the authors state that 'only 1/72 drug-eQTL interaction genes also shows evidence of differential gene expression'. This result indicates that the coefficients for drug exposure (beta_drug; or equivalently, the main effect of drug exposure) in the linear model may be weak, or even insignificant. What does drug interaction mean when the main effect is almost nonexistent? If the main effect is indeed nonexistent, then the significant interaction estimated from the linear model might be an artifact; the interaction effect may come from other relevant predictors that are not included in the model. The complete model would be helpful for addressing this issue. If beta_drug and beta_x_drug are still significant after IFN status is accounted for, then drug interaction would be providing additional information ('independent information' as the authors suggested) unaccounted for by differential gene expression induced by drug exposure.*

*By analyzing all the genes with this complete model, one can then more easily account for multiple testing, either with the stringent Bonferroni correction to control the overall type I error, or with the less stringent q value method to control for FDR.*

Response 2

We thank the reviewer for raising these important points. To address the first point, we do not find any association between anti-IL-6 exposure and IFN status (Fishers exact p=0.6). To further confirm the validity of our assumption that anti-IL-6 exposure and IFN status can be treated independently to identify eQTL interactions, we have generated a full model as suggested by the reviewer which includes IFN status, IFN interaction, drug status and drug interaction. We find that including the additional terms has very little effect on the interaction beta compared to the original analysis where anti-IL-6 and IFN were analysed separately, suggesting that anti-IL-6 exposure does not affect IFN eQTL interactions or vice versa.

Results: We analysed drug and IFN-eQTL interactions independently because anti-IL-6 exposure and IFN status are not associated (Fishers exact p = 0.6). However, to further ensure that these variables are independent, we repeated the interaction analysis with a full model including the drug, drug interaction, IFN and IFN interaction terms. We find that the interaction betas are highly concordant (rs=0.99) with the original analysis (**Figure S13**) for both IFN and drug-eQTL interactions providing further evidence that IFN status and drug exposure are independent.

We do note that the lack of association between IFN and anti-IL-6 is intriguing. Arguably an anticytokine therapeutic that is effective in SLE might be expected to result in altered IFN levels, given how central these cytokines are to SLE pathogeneis. We have added some text to the discussion to this effect:

Discussion: We do not find an association between anti-IL-6 exposure and IFN status and only eight of the cytokine eQTL interactions overlap. Arguably an anti-cytokine therapeutic that is truly effective in SLE might be expected to reduce IFN levels, given how central IFN is to SLE pathogenesis[46].

We agree with the reviewer that the ability to detect interactions with a full model could be an attractive approach and indeed we had considered it in our original implementation. We ultimately chose not to use this approach since we had a number of concerns in terms of the interpretability of such a model. Firstly, we note that interactions have much smaller effect sizes.

For the genes that we test for eQTL interactions, on average the eQTL term explains 12% of the variance, the differential expression term explains 2% and the interaction term explains 0.2%.

Therefore we recognize that significance of a model fit could be driven by the much larger eQTL or differential expression terms. Secondly, it is challenging to interpret the betas of the main effects in the full model. When an interaction is included, the coefficient of one main effect is conditional on the category of the other. For example, the differential expression term will represent the effect when the genotype is fixed (either as 0, 1 or 2) or vice versa.

To further address the point on differential expression, we have re-analyzed the differential expression without accounting for principal components. This is a more common strategy for assessing differential expression. Similar to most differential expression approaches, we have thresholded differential expression effects on a fold change >1.2. We still find that the minority of genes with an interaction also show evidence of differential expression (42/210 IFN, 0/72 drug). We would argue that these are still interesting interactions as they are the result of a change in variance of the gene rather than a change in mean as a result of the environmental perturbation.

Results: For IFN status, we identified 1,850 differentially expressed genes (FDR<0.05 and fold change>1.2, **Figure S16, Table S6**). Only 42/210 IFN-eQTL interaction genes also show evidence of differential expression. For differential expression following anti-IL-6 treatment, we identified 394 genes (FDR<0.05 and fold change>1.2, **Figure S16, Table S7**).

Discussion: We note that the majority of these results are independent of differentially expressed genes, which are more likely to represent second order effects, rather than primary genetic effects.

Comment 3

*Table S4 looks at correlation between the gene expression PCs in the linear model and potential confounders, such as age, sex and site. None of the PCs have a strong correlation with these confounders, meaning that these confounders contain information not captured by the PCs. Why are they not included in the linear model?*

Response 3

We agree with the reviewer on the importance of including potential confounders in the model. We favour PCs as they do not limit us to the confounders that have been measured in the study, but also capture confounding effects that are not explicitly captured but have global effects on expression.

However we recognise the importance of assessing that the PCs are capturing the effects of known confounders too. We did not initially include confounders in the model explicitly, since we felt that any confounders worth considering would affect a large number of genes and induce a principal component effect. Considering the reviewer’s comments, we have re-run the interaction analyses including age, sex and site in addition to the PCs and found they have very little effect on the interaction betas (IFN rs=0.991, drug rs=0.996) suggesting that the PCs are capturing these known confounders. We have included the following text in the manuscript to address this point.

Results: We included principal components as covariates in our model to account for confounding sources of gene expression variation that are not limited to those that have been measured in the study. **Table S4** summarizes the correlation between the principal components and potential known confounders such as age, sex and site of recruitment. As no single principal component strongly correlates with these known confounders, we re-ran the interaction analysis including age and sex as fixed effects and site as a random effect. The interaction betas are very highly correlated (rs=0.99) with the original effects suggesting the principal components are capturing these known confounders (**Figure S9**).

Minor comments:

Comment 4

*If the formatting requirements allow, it would be helpful to refer in the main text to specific sections in Methods, instead of simply 'Methods'.*

Response 4

We thank the reviewer for this excellent suggestion and have made the relevant updates to the text.

Comment 5

*The authors may want to streamline the presentation of the number of patients and the number of samples in the Methods.*

*Study design: 183 patients, 183x3=549 samples;*

*RNA-sequencing: 180 patients, 468-4=464 samples;*

*Genotyping: 159 patients;*

*Interferon status: 157 patients, 376 samples;*

*Free IL-6 protein levels: 145 patients, 311 samples;*

*Statistical analysis: 157 patients, 379 samples (numbers used in main text).*

Response 5

We have included Table 1 in the methods to summarize the patients and samples available for each data type.

Comment 6

*Line 242-249 on p 10, analysis of TF binding motifs. What do foreground and background*

*mean? Can the authors provide an example to illustrate these concepts?*

Response 6

We have modified text in the results and methods to clarify the description of the TF binding motif analysis.

Results: To determine enrichment, we compared the transcription factor motifs found in a set of sequences (containing the interaction SNPs) from one category of interactions relative to the other. We conducted two separate analyses: the proportion of magnifying eQTL interaction sequences with a motif compared to the proportion of dampening interaction sequences with a motif and vice versa.

Methods: For each environmental factor, we divided the eQTL interactions into magnifiers or dampeners and conducted two separate HOMER analyses: the proportion of magnifying eQTL interaction sequences with a motif compared to the proportion of dampening interaction sequences with a motif and vice versa. HOMER reported the transcription factor motifs that were significantly enriched in one category of interactions relative to the other.

Comment 7

*Line 564 on p 24 says that 'we determined the significance of the interaction term with a likelihood ratio test'. Can the authors elaborate on this? Table S1 summarizes the statistics for the eQTLs, but the statistics for interaction seem to be t statistics, which are used for an exact test in small samples, and not for a likelihood ratio test, which is typically an approximation at a large sample size. Which test was used then?*

Response 7

The t value corresponds to the coefficients from the model. To clarify, we have renamed this to “beta/SE” and have included the chi squared test statistic from the likelihood ratio test and renamed the p value to “chi_pval”.

Comment 8

*Line 512-513 on p 22, 'we first applied a linear model for the first available time point (week 0 samples for n=152, week 12 samples for n=5) to identify each eQTL...' Why is week 12 the first time point for 5 patients? Fig 1A suggests that all 157 patients have data from week 0.*

Response 8

Not all individuals have all time points available for RNA-sequencing. We have added the following to the figure legend to clarify that the figure represents the individuals rather than the samples.

Figure 1: (A) Clinical trial structure and sampling strategy for the individuals used for eQTL analysis. The available samples are summarized in Table 1.

Comment 9

*Line 253 on p 11, fig 3C should be 2C.*

Response 9

We have corrected this in the manuscript.

Comment 10

*What's the rationale behind the definition of the drug exposure score?*

Response 10

We have added the following text to the discussion to clarify the rationale of the drug exposure score.

Discussion: The rationale for this drug exposure score is that the expression of a drug-eQTL interaction gene will reflect the effectiveness of the drug in the individual but will be dependent on the genotype of the eQTL interaction SNP.

Comment 11

*Lupus_eQTL.zip is unavailable on the GitHub page:* [*https://baohongz.github.io/Lupus_eQTL/*](https://baohongz.github.io/Lupus_eQTL/) *(Mar 26, 2018).*

Response 11

We have corrected this link and the Lupus_eQTL.zip file can now be downloaded.

Comment 12

*The authors provide gene expression data in Table S6, where each row is a gene and each column is a sample. However, there is no information on which samples are from which patients with what treatment and from which time point. Perhaps the zip file on the GitHub page has this information?*

Response 12

We have added additional rows to Table S8 (formally Table S6) to indicate which sample is from which individual, treatment and time point.

**Second round of review**

**Reviewer 2**

The authors have carefully addressed most of the comments from the previous review. A minor suggestion is to write `Fishers exact" as \Fisher's exact" or \Fisher's exact test", as the test is named after R. A. Fisher.

The only outstanding question that remains is the differential expression analysis, and in particular, the DE analysis for drug-eQTL interacting genes. The related statement in the manuscript now reads: \none of the 72 drug-eQTL interaction genes show evidence of differential gene expression". During the previous review I found a similar statement surprising because I interpreted \differential expression" as referring to the main effect (beta drug), and it does not happen very often that the interaction effect is significant but the main effect is not. I initially thought that there was something wrong with the model. Now, with the newly added Table S7, I realize that it is due to different definitions of DE. The authors seem to define it as the mean expression difference between the exposed and unexposed groups, ignoring the eQTL. Both definitions are valid, but which one is more useful here?

The drug-eQTL interaction is detected through the following linear mixed effects model:

The differential gene expression with drug exposure is detected possibly through the following linear mixed effects model (when accounting for PCs):

For simplicity, we can group the random effect and the effects of the PCs together with the overall mean effect and have

When differential expression is defined ignoring the QTL and interaction effects, the drug exposure effect β*drug absorbs those two effects. In other words, in the exposed group, the mean drug exposure effect is the average over patients of different genotypes with or without the interaction effect.

Therefore, β*drug contains not only the true drug exposure effect, but also the effects due to the eQTL (βgeno and βx). In the unexposed group, the overall mean (the baseline) is also an average of patients with different genotypes.

Equations (3)-(5) imply three possible relationships between differential gene expression (ignoring the eQTL) and the two eQTL-related effects (when significantly different from 0):

1. βgeno and βx have the same sign. In this case, the two effects make contributions to gene expression in the same direction. Differential expression between exposed and unexposed is likely significant (i.e., β*drug is significantly nonzero).

2. βgeno and βx have opposite signs, but do not completely cancel each other out. Then differential expression may be detected between two treatment groups.

3. βgeno and βx have opposite signs, and cancel each other out (similar effect sizes). Then differential expression cannot be detected.

Using Tables S1 and S7, we can extract examples in different scenarios. All the gene-eQTL pairs below are among the 72 significant drug-eQTL interactions reported, ranked in an ascending order of the drug interaction p values. Drug DE qval is the q value for the main drug effect (βdrug) from model (1). We can further extract their interaction plots from the github tool provided by the authors, although it is worth pointing out that the range on the vertical axis can be very different in different plots.

We can extract examples of βgeno and βx having the same sign, with the corresponding plots in Fig 1. Considering the small q value for the main effect of ADORA1 and CHST13, it is surprising that they are not detected to be DE by model (2). Whether it is due to unequal sample sizes for different genotypes or additional confounding not accounted for, it seems informative to know that the main effects are significant for these two genes.

We can also extract examples of opposite signs with the plots in Fig 2. Note that the top two plots in this figure demonstrate a clear \crossover" pattern: one line changes from high to low, and another low to high. This means that again, comparing expression averaged over genotypes may not be helpful, as the main effect and the interaction effect cancel each other out.

Among the 72 drug-eQTL interactions, 57 genes have a main effect due to drug exposure with q value less than 20%, and 52 less than 10%. Therefore, most main effects are indeed significant, which is consistent with what we normally expect from a linear model with interactions.

It is fine for the authors to state that none of the drug-eQTL interaction genes show differential expression, but it would be beneficial for the authors and readers to be aware that this statement may not be informative and that the conclusion of no DE, defined this way, can mask the various types of impact of the eQTL on drug exposure.

**Reviewer 3**

I've had a chance to read through and the author response almost but do not fully address Reviewer 1's concerns. To be specific:

- Response 1: The comparison of the beta's is informative but not definitive as it doesn't account for changes in standard error between the 2 models. The authors should color on the plot and quantify the change in number of significant interactions with and without adjustment for gene expression PCs.

- Response 3: This is an important point which may impose a fundamental change to the paper's interpretation as to the true source of the interaction. Without suitable cell composition data to adjust for, it would be necessary to use appropriate language throughout the manuscript noting this quite reasonable alternate interpretation and clearly noting it as a major limitation in the Discussion (which as far as I can tell hasn't been done). FACS on B and T cell counts is useful but doesn't cover all the relevant cell types (monocytes, DCs etc). Furthermore, the authors have not done all that they could do with what they have, which is directly adjusting for B and T cell counts/proportions in their interaction model (and then comparing the 2 models like in Response 1, or ideally via a likelihood ratio test)

**Authors’ response to reviewers**

**Reviewer #2**

*Minor Comment 1: A minor suggestion is to write ‘Fishers exact” as “Fisher’s exact” or “Fisher’s exact test”, as the test is named after R. A. Fisher.*

We thank the reviewer for noting this error. We have made the following correction to the manuscript.

**Results:** Fisher’s exact test p = 0.6

*Minor Comment 2: It is fine for the authors to state that none of the drug-eQTL interaction genes show differential expression, but it would be beneficial for the authors and readers to be aware that this statement may not be informative and that the conclusion of no DE, defined this way, can mask the various types of impact of the eQTL on drug exposure.*

We thank the reviewer for their detailed discussion on the presentation of differential expression. We found this discussion very informative and enlightening. In particular, it highlighted that 2 drug-eQTL genes are indeed also differentially expressed using our described criteria. As suggested by the reviewer, we have included the following addition to the discussion of the manuscript to clarify that we are presenting the traditional approach for determining differential expression where genotype is not taken into account.

**Results:** Only 2/72 drug-eQTL interaction genes show evidence of differential gene expression.

**DIscussion:** While we find that the majority of the eQTL interactions that we identify are independent of differentially expressed genes, we have used the common strategy for identifying differential expression, which does not take into account the genotype of the individuals. The differential expression presented here therefore represents the average change in expression across all genotypes, regardless of any eQTL or interaction effect. Furthermore, like most differential expression approaches, we have employed a fold change cut-off. Using statistical evidence alone, 121/210 IFN-eQTL interaction genes show evidence of differential expression (FDR<0.05) and 45/72 drug-eQTL interaction genes. This approach highlights that many interactions are being driven by changes in variance of gene expression across the environmental variables rather than necessarily changes in mean expression and therefore eQTL interactions can offer additional information to what is identified through traditional differential expression analysis.

**Reviewer #3**

*Reviewer #3: I've had a chance to read through and the author response almost but do not fully address Reviewer 1's concerns.*

We thank the reviewer for their efforts in checking our responses. We have revisited the highlighted issues bought up by the reviewer and have tried to diligently address them.

*Reviewer Response 1: The comparison of the beta's is informative but not definitive as it doesn't account for changes in standard error between the 2 models. The authors should color on the plot and quantify the change in number of significant interactions with and without adjustment for gene expression PCs.*

We thank the reviewer for their comment and as suggested have colored the plot to

display the significance of the interactions without adjustment for gene expression PCs (see below).

The use of PCs to increase power to detect eQTL main effects has been recognized in the community widely (Pickrell et al. 2010, Liang et al. 2013, Westra et al. 2013). Indeed, we observed in our own data that correcting for the gene expression PCs increases our power to detect the main eQTL effect. When we do not correct for PCs, 107/210 IFNeQTL interactions no longer have a main eQTL effect that passes our Bonferroni corrected p value, due to an increase in standard error. We believe that these 107 eQTL main effects are real effects as 85% are also reported in the independent BIOS (Zhernakova et al. 2017) dataset (consistent with the same proportion that we observer across our full eQTL set). In terms of the eQTL interactions, we observe a similar phenomenon. When we do not correct for gene expression PCs, only 23/210 IFN-eQTL interactions retain an FDR<0.2 (with a further 52 retaining nominal significance with FDR between 0.2 and 0.5). This result is not surprising since as the reviewer notes, the standard error can differ between the two models. Indeed, as expected, correcting for the principal components reduces the standard error of the interaction estimates (see figure above). So, while our interaction betas remain highly correlated, the power to statistically identify an interaction is reduced when PCs are not corrected for. As noted above, in 107 of these instances we lose the power to significantly detect the main eQTL effect as well, and that almost certainly reduces our power to detect eQTL interactions. Importantly, despite this loss of power, the beta effects are largely unchanged, arguing against the idea that including PCs are systematically altering the interaction effects.

**Results:** While we find the betas for the interaction term are highly correlated (rs=0.88, **Figure S11a**), only 23/210 of our IFN eQTL interactions remain significant with an FDR<0.2 without correcting for any expression principal components. This reduction in significant interactions is likely due to the larger standard errors of the interaction estimate that are observed when principal components are not corrected for (**Figure S11b**). Furthermore, 107/210 of these interactions no longer have a main eQTL effect (passing our Bonferroni corrected p value threshold) without principal component correction, further reducing our power to detect significant interactions.

*Response 3: This is an important point, which may impose a fundamental change to the paper's interpretation as to the true source of the interaction. Without suitable cell composition data to adjust for, it would be necessary to use appropriate language throughout the manuscript noting this quite reasonable alternate interpretation and clearly noting it as a major limitation in the Discussion (which as far as I can tell hasn't been done). FACS on B and T cell counts is useful but doesn't cover all the relevant cell types (monocytes, DCs etc). Furthermore, the authors have not done all that they could do with what they have, which is directly adjusting for B and T cell counts/proportions in their interaction model (and then comparing the 2 models like in Response 1, or ideally via a likelihood ratio test)*

We thank the reviewer for their comments and agree with the importance of understanding how cell counts may be contributing to the observed interactions. We appreciate the suggestions from the reviewer and as such have conducted analysis where we include the B and T cell proportions from FACS data in our interaction models and compared the models, like in Response 1. We also agree with the reviewer that these data do not cover all the relevant cell types. We have therefore conducted additional analysis to explore cell proportion deconvolution from the RNA-seq data using the CIBERSORT software. We used this to deconvolute the relative proportions of nine hematopoietic populations (eosinophils, neutrophils, B cells, T cells, natural killer cells, macrophages, dendritic cells, mast cells and monocytes). Finally, recognizing the limitations of these analyses, we have also highlighted the possibility that cytokine shifts could be altering cellular populations not detected by these actual or inferred cell counts or the principal components included in our analysis, and there is the possibility that cytokine shifts are specifically altering individual cellular populations, thereby producing apparent interactions.

We started by determining the contribution of cell proportion to the IFN-eQTL interactions as this was where we had the greatest power to detect interactions. We included the cell proportions from either FACS or CIBERSORT as additional covariates in the model and then tested the significance of the interaction term using a likelihood ratio test. After correcting for either B and T cell proportions from the FACS data or the hematopoietic populations from CIBERSORT, we found the interaction betas to be highly correlated (rs=0.99 and rs=0.998 after correcting for 2 FACS and 9 CIBERSORT proportions respectively, see figures below). Furthermore, 162/210 and 189/210 IFNeQTL interactions remain significant (FDR<0.2) following these corrections suggesting that cell proportions are potentially driving very few IFN-eQTL interactions.

We observe a similar pattern for the drug-eQTL interactions (see figures below) but since these interactions are weaker, their effects are more sensitive to inclusion of covariates. The interaction betas are again highly correlated with and without correction (rs=0.98 and rs=0.993 after correction for 2 FACS and 9 CIBERSORT proportions respectively). However, only 15/72 and 54/72 drug-eQTL interactions remain significant (FDR<0.2) after these corrections. After FACS proportion correction, a further 53 interactions have a nominal significance with FDR between 0.2 and 0.5. After CIBERSORT proportion correction, all the interactions have nominal significance with FDR <0.5. This reduction in significant interactions suggests that some of these interactions may be driven by changes cell proportions however, given the relatively small effect size of the drug interactions, it could also be the result of reduced power to detect interactions following the inclusion of additional covariates in the model.

In light of this additional analysis with cell proportions from both FACS data and CIBERSORT deconvolution, we have added a section to the results in the manuscript. We have also made the following revisions throughout the manuscript to note this alternate interpretation and have stated the lack of cell counts as a limitation of the study.

**Background:** Therefore, observing a set of eQTL interactions due to a perturbagen, such as a cytokine, can identify shared upstream regulatory mechanisms, such as transcription factors and key pathways. Alternatively, a set of shared eQTL interactions may be the consequence of a cellular subpopulation whose frequency is being altered by the perturbagen.

**Results:**

*Contribution of cell proportions to eQTL interactions*

Given that we have conducted our study on whole blood, the observed eQTL interactions could be the consequence of a cellular subpopulation whose frequency is being altered by the environmental perturbagen or variability in cell type proportions between individuals. To explore this, we first determined B and T cell abundance from FACS data (Methods: Cell counts). We find that IFN and exposure to anti-IL-6 are not correlated with either B or T cell abundance (Figure **S17**) suggesting that the proportions of these particular cell types are not being altered by the shifts in cytokine levels.

We then went on to explore the effect of correcting for cell proportions on our eQTL interaction effects. As our FACS data do not cover all relevant cell types, we also inferred the relative proportions of nine hematopoietic populations from the RNA-seq data using CIBERSORT[38]. For IFN, the interaction betas remain highly correlated (rs= 0.99, rs=0.998, **Figure S18**) after

correcting for either B and T cell proportions from the FACS data or the 9 hematopoietic proportions from CIBERSORT. Furthermore, the majority of the interactions (162/210 and 189/210) remain significant (FDR<0.2) after these respective corrections. We observe a similar pattern for the drug-eQTL interactions (**Figure S19**) where the interaction betas are again highly correlated (rs=0.98 and rs=0.993 for FACS and CIBERSORT proportion correction respectively).

However only 15/72 and 54/72 drug-eQTL interactions remain significant after these corrections. This reduction in significant interactions suggests that some of these interactions may be related to changes in cell proportions. However, given that the interaction betas are so highly correlated and the relatively small effect size of the drug interactions, this could also be the result of reduced power to detect interactions following the inclusion of additional covariates in the model.

**Discussion:** We note that as we have conducted our study on whole blood, some of our observed interactions could be driven by variability in cell type proportions between individuals or as a consequence of cellular subpopulation frequencies being altered by the environmental perturbagen. A limitation of this study is that we lack the complete blood counts to explore this thoroughly. However, we determined B and T cell abundance from FACS data and used CIBERSORT[38] to deconvolute the relative proportions of nine hematopoietic populations from the RNA-seq data to explore this (**Methods: Cell counts**). While the number of significant eQTL interactions is reduced after correcting for cell populations, particularly for drug-eQTL interactions after correcting for the FACS proportions, the interaction effects remain very highly correlated suggesting that the majority of these effects are not being altered by these cell compositions.

However, further studies will be required to determine if cytokine shifts are altering cellular populations that were not detected by these actual or inferred cell counts, or the principal components that we included in our analyses. For future studies, it will be informative to quantify a broader range of relative cell types and data from single-cell technologies may be particularly powerful for determining cell type specific eQTLs[45] and their interactions.

**Methods:** We used CIBERSORT[38] to deconvolute proportions of cell types from the RNA-seq data. We used the LM22 database from CIBERSORT which contains cell signatures for 22 cell types and grouped these into 9 representative cell types (eosinophils, neutrophils, B cells, T cells, natural killer cells, macrophages, dendritic cells, mast cells and monocytes).
